# Investigation of the Basic Steps in the Chromosome Conformation Capture Procedure

**DOI:** 10.3389/fgene.2021.733937

**Published:** 2021-09-20

**Authors:** Oleg V. Bylino, Airat N. Ibragimov, Anna E. Pravednikova, Yulii V. Shidlovskii

**Affiliations:** ^1^Department of Gene Expression Regulation in Development, Institute of Gene Biology, Russian Academy of Sciences, Moscow, Russia; ^2^Center for Precision Genome Editing and Genetic Technologies for Biomedicine, Institute of Gene Biology, Russian Academy of Sciences, Moscow, Russia; ^3^Department of Biology and General Genetics, I.M. Sechenov First Moscow State Medical University, Moscow, Russia

**Keywords:** chromatin conformation capture, chromosome conformation capture, chromatin, distal interaction, DNA, *Drosophila*

## Abstract

A constellation of chromosome conformation capture methods (С-methods) are an important tool for biochemical analysis of the spatial interactions between DNA regions that are separated in the primary sequence. All these methods are based on the long sequence of basic steps of treating cells, nuclei, chromatin, and finally DNA, thus representing a significant technical challenge. Here, we present an in-depth study of the basic steps in the chromatin conformation capture procedure (3С), which was performed using Drosophila Schneider 2 cells as a model. We investigated the steps of cell lysis, nuclei washing, nucleoplasm extraction, chromatin treatment with SDS/Triton X-100, restriction enzyme digestion, chromatin ligation, reversion of cross-links, DNA extraction, treatment of a 3C library with RNases, and purification of the 3C library. Several options were studied, and optimal conditions were found. Our work contributes to the understanding of the 3C basic steps and provides a useful guide to the 3C procedure.

## Introduction

A chromatin conformation capture (3C) method is probably one of the most complex protocols in molecular biology, mainly due to its multistep nature (Table 1). The steps should be done in proper order and require careful execution. Incorrect implementation of the steps leads to poor restriction enzyme digestion, ineffective ligation, degradation, and/or loss of DNA. Importantly, a mistake made at any of the stages becomes known only at the very end of the procedure. Numerous controls therefore used, which are selected at different time points of protocol execution (Dekker, 2006).

**Table 1:** Basic steps of the 3C procedure.

Step	Characteristic of the step
1	Cell fixation, formaldehyde (FA) inactivation, and storage of nuclei
2	Cell lysis
3	Nucleoplasm release and chromatin treatment with heat (nuclei heating in the presence of SDS and Triton X-100/SDS sequestration with Triton X-100)
4	Digestion of DNA in nuclei
5	Ligation of DNA in nuclei
6	Reversion of cross-links and isolation of a 3C library
7	Treatment of the 3C library with RNases
8	Purification of the 3C library on magnetic beads

The experimental literature on chromosome conformation capture methods (C-methods) is quite extensive. However, the literature lacks systematic analysis of how exactly the basic steps of the protocol work. At the same time, there are a lot of studies where individual selected steps were investigated (Gavrilov and Razin, 2009; Comet et al., 2011; Gavrilov et al., 2013b, 2015; Nagano et al., 2015b; Oksuz et al., 2020; Gridina et al., 2021). In this article, we try to fill this gap and explore in detail all the basic steps of the 3C protocol at once. A methodology for determining whether remote DNA regions can interact with each other in nuclei was first proposed by Cullen et al. (1993) and was called the nuclear ligation assay (NLA). In NLA, ligation of restriction endonuclease (RE)-digested chromatin was carried out in mammalian isolated intact nuclei using T4 DNA ligase and the frequency of ligation between regulatory elements was estimated by PCR (Cullen et al., 1993). In 2002, the methodology was finalized by Dekker et al. who supplemented the structure of the method with a fundamental step of cell fixation with formaldehyde (FA) and its subsequent quenching with glycine before chromatin digestion and ligation. The cross-linking of chromatin preserved the nuclear structure intact throughout the procedure without affecting its flexibility (Dekker et al., 2002). Data processing into genome-wide chromatin interaction maps was also proposed, suggesting that the 3C approach implemented on a yeast model can be applied to determine the spatial organization of whole genomes from bacteria to humans (Dekker et al., 2002). In 2009, a genome-wide 3C method was proposed and termed Hi-C. The method allows measuring the contact frequencies of all chromatin interactions that occur in the nucleus in a single experiment (Lieberman-Aiden et al., 2009). This area of research, called 3D genomics (Razin et al., 2019; Zhang and Li, 2020), is currently developing rapidly.

More recently, the basic principles of the method have been reassessed and an alternative strategy has been proposed, wherein a combination of two fixing agent (formaldehyde (FA) and disuccinimidyl glutarate (DSG)) and of two frequently cutting REs is used. This strategy, entitled Hi-C 3.0, should, in future, complement or even substitute the standard approach that is based on fixation with FA only and utilizes one frequently cutting RE (Oksuz et al., 2020).

**Figure 1:**
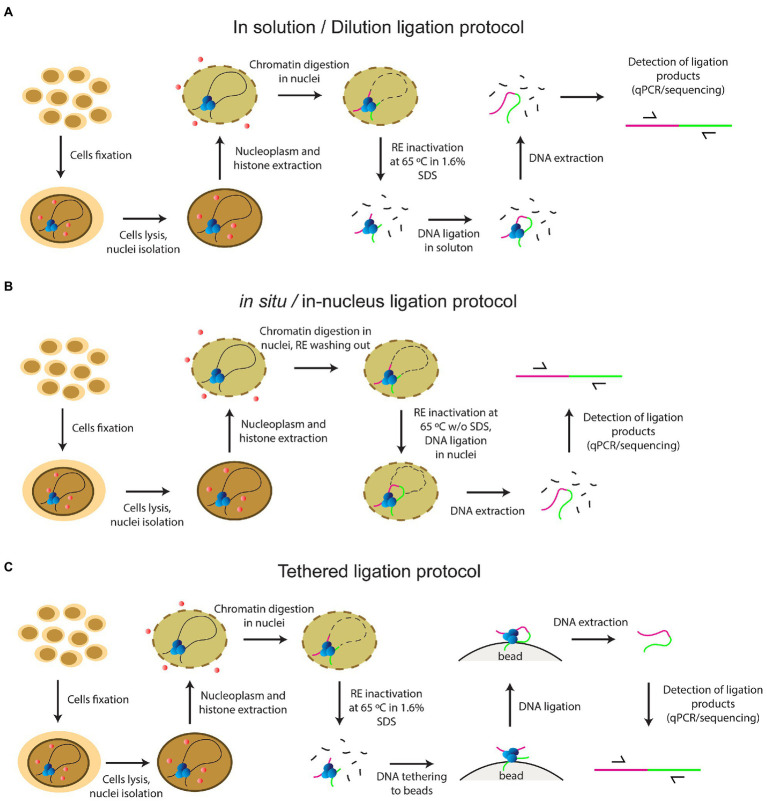
The principles of current protocols of C-methods **(A)** in solution (dilution) ligation protocol **(B)** *in situ*/in-nucleus ligation protocol **(C)** tethered ligation protocol.

At the same time, there remained a considerable lack of clarity regarding the steps of the classical version of the procedure. Since 2002, several add-ons and variations have been introduced into the basic 3С protocol by different working groups. Adding extra steps yielded a whole panel of the so-called C-methods, i.e., 4C, 5C, Hi-C, and so on, all making it possible to determine different aspects of the 3D genome organization in the cell nucleus (Zhang and Li, 2020). Various C-techniques have been standardized in the framework of the international 4D nucleome program (Dekker et al., 2017). Despite the phenomenal variety of existing C-methods, they all utilize the same basic steps, including fixation of cells with FA, cell lysis, nucleoplasm extraction, chromatin endonuclease digestion, ligation of the resulting DNA fragments, reversion of DNA-DNA and DNA-protein links by heating, and subsequent isolation and analysis of contact frequencies between all or specific fragments. Initial stages of the development of the method since 2002 have employed an in-solution (dilution) ligation protocol; i.e., solubilized chromatin fragments were ligated in a highly diluted solution (Dekker et al., 2002; Tolhuis et al., 2002; Figure 1A). In 2012, a tethered Hi-C protocol was proposed (Figure 1C). The protocol also involved solubilization of chromatin fragments, like the dilution protocol. The difference was that cross-linked DNA fragments were ligated not in a large volume of solution, but on the surface of the solid phase of streptavidin-coated beads. Bound chromatin fragments were spatially separated from each other on the solid phase, which enhanced the signal-to-noise ratio as needed for detecting chromatin loops, and helped to avoid trans-ligation events, ensuring that ligations in these libraries are between DNA fragments that are cross-linked to each other (Kalhor et al., 2012; Gabdank et al., 2016). A Dounce homogenizer was used to isolate nuclei in this protocol even with cultured cells, and the library was processed with bovine RNase A (Kalhor et al., 2012; Gabdank et al., 2016).

In 2011, a key work in terms of the development of the C-methodology was carried out with a *Drosophila* model. Comet et al. (2011) showed for the first time that the same 3C profile was obtained with a complete non-fractionated sample and an insoluble chromatin fraction, while a 3C signal of a sufficient intensity was not detected with the supernatant fraction (Comet et al., 2011). The same result was demonstrated a couple of years later with mammalian cells, confirming the universality of this observation (Gavrilov et al., 2013b). The focus has changed since that time, and an *in situ* protocol came into use in 2014, involving cross-ligation of fragments within the nucleus (Rao et al., 2014; Figure 1B). This approach prevents random ligation between chromatin fragments released in solution by utilizing ligation *in situ*, i.e., in intact nuclei. The *in situ* protocol made it possible to obtain maps of very high quality and resolution under a sub-kilobase scale due to several distinctive features: a Dounce homogenizer was not used to obtain cell nuclei from cultured cells; a four-base pair RE was used instead of a six-base pair cutter; RE was inactivated by heating at 65°C for 20min without adding 1.6% SDS, unlike in the dilution protocol; and ligation was performed with the nuclear fraction without large sample dilution (in ~1ml). These circumstances reduced the frequency of non-specific contacts due to random ligation in dilute solution, – as was evidenced by a lower frequency of junctions between mitochondrial and nuclear DNAs and a higher frequency of random ligations observed when the supernatant fraction was sequenced (Rao et al., 2014). In addition, Rao’s protocol did not include a processing of the library with RNase A.

In fact, proposals to avoid SDS treatment by inactivating the RE by heating at 65°C for 20min and to use SDS only to inactivate heat insensitive enzymes have been expressed earlier (Splinter et al., 2012). However, the idea that ligation occurs mainly in the nuclear fraction and the structure of the nucleus should therefore be kept as intact as possible came only after the works of (Comet et al. (2011) and Gavrilov et al. (2013b) and crystallized in Rao et al. (2014) as an *in situ* protocol.

In 2013, single-cell Hi-C was done for the first time (Nagano et al., 2013). In 2015, an in-nucleus ligation protocol was proposed in the development of this approach (Figure 1B). The in-nucleus ligation protocol is similar to the *in situ* protocol but utilizes even milder chromatin processing procedures. The step of RE inactivation by heating at 65°C in the presence of SDS was completely eliminated from the protocol and ligation was thus performed in nuclei preserved to a maximal possible extent. It was found that the in-nucleus ligation protocol results in consistently lower levels of spurious ligation events manifested in trans-chromosomal contacts, reduces the experimental noise, and eliminates restriction fragment length bias found with the in-solution ligation protocol (Nagano et al., 2015a,b, 2017). Also, this protocol does not employ a Dounce homogenizer to prepare a suspension of isolated nuclei.

In 2012, the Hi-C method was first applied to *Drosophila* cell culture (Hou et al., 2012), a model that we used in this study, and late embryos (Sexton et al., 2012). The two studies showed the principles of *Drosophila* genome organization into well-demarcated physical domains. In 2015, a work was published to demonstrate for the first time that a reconfiguration of topologically associated domains (TADs) occurs in response to cell stress was observed (Li et al., 2015). These early works all used the dilution protocol. The *in situ* protocol was employed in later works, which investigated the role of architectural proteins in enhancer-promoter interactions and TADs structure (Cubeñas-Potts et al., 2017; Rowley et al., 2017; Chathoth and Zabet, 2019). The *in situ* protocol was also used in single-nucleus Hi-C with cultured *Drosophila* cells (Ulianov et al., 2021). In 2017, a variant of the in-nucleus ligation protocol was applied to study the long-range interactions in *Drosophila* embryos (Stadler et al., 2017). During the experimental procedure, the RE was inactivated without using SDS or higher temperature by washing out from the nuclei, but embryos were homogenized using a Dounce homogenizer to prepare the nuclei (Stadler et al., 2017). Despite the general shift to the *in situ*/in-nucleus ligation protocol, the dilution protocol is still used in studies of the *Drosophila* genome organization (Ulianov et al., 2016, 2019; El-Sharnouby et al., 2017; Lo Sardo, 2021). The tethered ligation protocol was also successfully applied in two works with *Drosophila* (Eagen et al., 2015, 2017).

Thus, several protocols based on different principles of ligation are used in parallel today. The protocols differ in the conditions of basic steps: FA fixation and FA inactivation, cell lysis, the buffers used, the procedure of isolation and washing of nuclei, the severity of chromatin treatment, the conditions of chromatin restriction, RE inactivation, DNA ligation, and isolation and processing of the library with RNases.

Here we describe our study of the basic steps of the 3C procedure; offer our own options, which were found to be optimal in our hands; and provide the protocol suitable for S2 cells. We validate our results by showing with a model locus that efficient chromatin digestion and ligation occurs at an acceptable level, making it possible to distinguish the products of target ligation from the background of uncleaved DNA. Our procedure demonstrated its efficiency not only with cultured cells, but also with living objects, namely, − *Drosophila* larvae (Shidlovskii et al., 2021). Our work contributes to the understanding of the basic steps of the 3C procedure.

## Cells Fixation, FA Inactivation, and Storage of Nuclei

Several modes of cells fixation with FA and FA inactivation with glycine have been proposed in the literature for the 3C procedure. The amount of FA used for fixation varies from 1 to 3%, while the amount of glycine used for inactivation of FA can be classically 0.125M (Dekker et al., 2002; Tolhuis et al., 2002), equimolar or in excess to FA (Comet et al., 2011; Sexton et al., 2012). In turn, the FA concentration is influenced by the composition of the buffer in which fixation takes place. For example, the buffer used to isolate and fix the nuclei should not include Tris because Tris contains reactive amines, which cross-link FA to Tris, leaving less FA to fix DNA and proteins (Louwers et al., 2009), although original Dekker’s protocol for yeast cells utilizes Tris. In the case of mammalian cells, it was originally proposed to add FA directly into DMEM supplemented with 10% FCS (Tolhuis et al., 2002), and DMEM contains many amino acids with reactive amines that bind FA, as Tris does. Therefore, cells were fixed in 1X PBS at room temperature (RT) in our experiments. For fixation, a 2X formulation of PBS was mixed in equal proportions with an aqueous solution of FA of a necessary concentration. Alternative fixation buffers (15mM HEPES-KOH pH 7.6, 60mM KCl, 15mM NaCl, 4mM MgCl_2_, 0.1% Triton X-100, 0.5mM DTT, and protease inhibitor cocktail) can be used as well (Comet et al., 2011; Sexton et al., 2012).

The first thing we observed was that DNA from cells fixed with 2% FA for 10 min was poorly isolated by phenol/chloroform (Ph/Chl) extraction immediately after fixation (FA was inactivated with equimolar glycine concentration; e.g., Supplementary Figure S4D, lane 16). Attempts at cross-links reversion of such DNA (overnight incubation at 50°C; Supplementary Figure S4B, lane 8) or at keeping such cells after fixation at −20°C overnight (Supplementary Figure S4H) unexpectedly aggravated the situation: DNA was not isolated by Ph/Chl extraction at all. Moreover, after keeping such cells at −20°C for 2weeks, the DNA seemed to be fixed very firmly and was not isolated, even in much more severe isolation conditions using a protocol for FA fixed tissues (Supplementary Figure S4I; Campos and Gilbert, 2012). It was concluded that cells treated for 10min with 2% FA were almost over-fixed and their DNA could not be isolated after further incubation or storage at −20°C. After fixation at other concentrations of FA (0.5–1.5% FA) for 10min, DNA could be successfully isolated (FA was inactivated with equimolar glycine concentration; Supplementary Figure S4B, lanes 5–7; Supplementary Figure S4F, lanes 5, 6). Thus, we have established 1.5% as an upper limit for the concentration of FA at which DNA is not fixed too firmly in 10min.

These results suggest that the storage of cells fixed with FA may be detrimental. We assume that cell fixation continues until the cells are completely frozen at −20°C, especially when glycine is used in a strong molar deficiency (0.125 M). We recommend to avoid storing the fixed material (or flash freezing using liquid nitrogen) and to immediately proceed to cell lysis after washing fixed cells.

The 0.125M glycine concentration was taken for 3C protocols from ChIP protocols (Orlando et al., 1997) in which this concentration is the standard (Kim and Dekker, 2018a,b). As it was previously pointed out, quenching is not likely to be complete in the presence of 0.125M glycine because glycine is not in excess over FA (Splinter et al., 2012). Over-fixation of the material, in this case, may therefore occur during freezing at −20°C or even thawing of cells. If inactivation was done with 0.125M glycine to favor reproducibility between experiments, it was recommend immediately to proceed to the next step after quenching (Splinter et al., 2012).

Thus, we also do not recommend inactivating FA with glycine in a strong molar deficiency (0.125M) and storing nuclei inactivated in this way at −20°C. Instead, we recommend using glycine in an equimolar amount or in slight excess to FA (Comet et al., 2011; Sexton et al., 2012), keeping in mind two reactive groups of FA vs. one group of glycine. Thus, we used glycine at 666 (equimolar) and 800 (slight excess) mm for 1% of FA (Supplementary Table S1).

Empirically, we chose 10min to fix cells properly. Shorter incubation times will result in lower detection signals of chromatin interactions, whereas longer incubation times will cause too many DNA-protein cross-links, resulting in a reduced digestion efficiency (van Berkum and Dekker, 2009). We observed that 25-min fixation leads to cell over-fixation. Over-fixed cells withstand digestion with Proteinase K (PrK) in the presence of 1% SDS, and DNA from such cells is not extracted with Ph/Chl.

We found that glycine used in a slight (about 20%) excess to FA does not change the PCR signal. A larger excess of glycine (for example, 800mM glycine vs. 333mM FA) leads to the formation of a sticky pellet that does not go down along the wall of the tube. The signal intensity of such samples in PCR is usually 3–4 times lower than in the case of equimolar inactivation of FA with glycine.

It was proposed to incubate cells on ice for 15min after 5-min quenching with glycine at RT in order to stop the cross-linking completely (van Berkum and Dekker, 2009). The same processing step was included in the *in situ* protocol (Rao et al., 2014). We combined the stage of complete cessation of cross-links on ice with the stage of cell lysis in our procedure (see “Cell Lysis”).

## Cell Lysis

The composition of the cell lysis buffer was the next important issue that we investigated. The 3С literature describes several fundamentally different compositions of lysis buffers. The two main buffers that are currently in use are a hypotonic buffer with a low concentration of a non-ionic detergent (10mM Tris-HCl pH 8.0, 10mM NaCl, and 0.2% NP-40), which is a classical buffer and was first introduced in the work (Tolhuis et al., 2002), and an isotonic buffer with a higher concentration of non-ionic detergents (50mM Tris-HCl, pH 7.5, 150mM NaCl, 0.5% NP-40, 1% Triton X-100, and 5mM EDTA), which was first introduced in the works (Splinter et al., 2012; van de Werken et al., 2012). The first buffer inherited the detergent content from the pioneering work by Cullen et al. (1993). The second buffer was proposed as an alternative to the first one, contains an increased amount of non-ionic detergents, and is designed to ensure effective cell lysis and easy release of nuclei without the use of a Dounce homogenizer (Splinter et al., 2012). Several alternative lysis buffers have also been proposed in the literature. Examples include a hypotonic buffer that contains Mg^2+^, is completely devoid of detergents (10mM Tris-HCl, pH 7.5, 10mM NaCl, 5mM MgCl2, and 0.1mM EGTA), and requires the use of a Dounce homogenizer (Hagège et al., 2007), and a medium salt buffer that contains Mg^2+^ and has a low content of non-ionic detergent (15mM HEPES-KOH pH 7.6, 60mM KCl, 15mM NaCl, 4mM MgCl_2_, 0.1% Triton X-100, and 0.5mM DTT; Comet et al., 2011), in which the Na^+^ and K^+^ concentrations were taken from the work (Cullen et al., 1993).

After reviewing the variety of existing lysis buffers, we decided to test the contributions of individual components of the lysis buffer and composed 11 buffers, which differed in major component composition (Table 2).

**Table 2:** The lysis buffers studied.

Buffer	50mM Tris-HCl, pH 8.0	10mM Tris-HCl, pH 8.0	150mM NaCl	10mM NaCl	0.5% NP-40	0.2% NP-40	1% Triton X-100	5mM MgCl_2_	0.1mM EGTA
1	+		+				+		
2	+		+			+	+		
3	+		+		+		+		
4	+		+				+	+	
5		+		+		+			
6		+		+					
7		+		+				+	
8		+		+				+	+
9	+			+			+		
10	+			+		+	+		
11	+			+	+		+		

**Figure 2:**
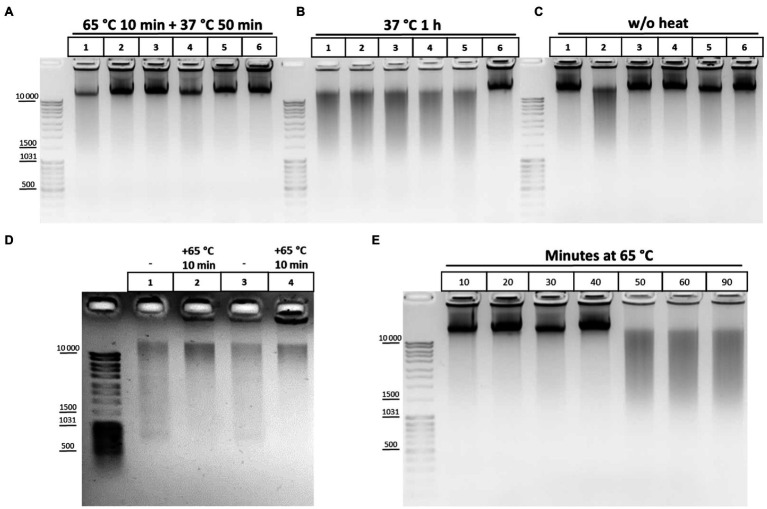
Cell lysis buffer composition and temperature regimens of chromatin treatment affect DNA integrity. **(A-C)** Effect of the lysis buffer composition on the integrity of the resulting DNA. 10mg (~12 mln of cells) of S2 cells was fixed with 1% FA in 1X PBS at RT for 10min, quenched equimolar with glycine for 5min at RT, washed twice with 1X ice-cold PBS, and then resuspended in ice-cold lysis buffers #1, 2, 3, 9, 10, and 11 according to Table 2. Lane 1 – Buffer #1; Lane 2, #2; Lane 3, #3; Lane 4, #9; Lane 5, #10; and Lane 6, #11. Cells were incubated in different lysis buffers on ice for 15min and were then washed once with ice-cold 1X PBS and resuspended in 1X RB for DpnII (50mM Bis-Tris-HCl, 100mM NaCl, 10mM MgCl_2_, 1mM DTT, and pH 6.0 at 25°C). Then, SDS was added up to 0.1% **(A,B)** and the nuclei were treated with heat at 65°C for 10min+37°C for 50min **(A)** or at 37°C for 1h **(B)** and then Triton X-100 was added up to 1.8% **(A,B)** and the nuclei were incubated at 37°C for 1h **(A,B)**. Thereafter, 500μl of EB (50mM Tris-HCl pH 8.0, 50mM NaCl, and 1% SDS; Hug et al., 2017) containing 30mM EDTA and 0.2mg/ml PrK was added. The cross-links were reversed in EB at 65°C overnight (O/N) and the DNA was extracted with Ph/Chl and subsequently with Chl only, precipitated with 3V of 96% ethanol, 0.3M NaOAc pH 5.2, and 100μg of glycogen, washed with 70% ethanol three times, dissolved in 25μl of 10mM Tris-HCl pH 8.0, and treated with 0.2–0.4mg/ml of bovine RNase A for 30min at RT. The amount of DNA subjected to electrophoresis was 1μl. For **(C)**, the cells were treated in the same way as for **A** and **B**, but the step of chromatin heat treatment in the presence of SDS and Triton X-100 was omitted and instead, immediately after resuspension of nuclei in 1X RB, 500μl of EB was added, supplemented with the same amount of SDS and Triton X-100 as for **A** and **B**. The reversion of cross-links and DNA extraction was performed in the same way. One of the three replicate experiments is shown. **(D)** Chromatin heat treatment at 65°C results in some DNA preservation during subsequent isolation. Сells were fixed, quenched, washed after quenching with 1X PBS as in **A**-**C,** and lysed in isotonic lysis buffer #3 (see Table 2; Tolhuis et al., 2002; lanes 1, 2) or in hypotonic lysis buffer #10 (Splinter et al., 2012; van de Werken et al., 2012; lanes 3, 4). Cells were incubated in lysis buffers on ice for 15min and then nuclei were centrifuged, supernatant was discarded, nuclei were resuspended in 1X RB, SDS was added up to 0.3%, and nuclei were incubated at 65°C for 10min (lanes 2, 4) or left untreated (lanes 1, 3). Thereafter, 500μl of EB was added, the cross-links were reversed as in **A**-**C**, and the DNA was extracted using the GeneJET Genomic DNA purification kit (Thermo), according to the manufacturer’s instructions. **(E)** DNA degradation upon chromatin treatment at 65°C. Сells were fixed, quenched, washed after quenching with 1X PBS as in **A**-**C**, and then lysed in isotonic lysis buffer #3 (see Table 2). Cells were incubated in lysis buffer on ice for 15min and then nuclei were washed once with 1X ice-cold PBS, resuspended in 1X RB for DpnII, and then SDS was added up to 0.1%. Then, nuclei were treated with heat at 65°C for 10, 20, 30, 40, 50, 60, and 90min in the presence of 0.1% SDS. After that, the nuclei were incubated for up to 1h at 37°C for 50, 40, 30, 20, and 10min (lanes 1–6, respectively). Then, Triton X-100 was added up to 1.8% and the nuclei were incubated at 37°C for 1h. In the case of lane 7, after incubation in 0.1% SDS at 65°C for 90min, Triton X-100 was added immediately up to 1.8% and nuclei were incubated at 37°C for 1h. After incubation with SDS/Triton X-100500μl of EB was added to all samples, the cross-links were reversed and the DNA was extracted, precipitated, dissolved, and treated with bovine RNase A and subjected to electrophoresis as in **A**-**C**. One of the three replicate experiments is shown.

Of these, buffers 1, 2, 3, 9, 10, and 11 appeared to provide the best results in terms of maintaining DNA integrity and were further tested under more variable chromatin treatment conditions. The results of a representative experiment are shown in Figure 2. The composition of the lysis buffer affects the integrity of the resulting DNA under different regimens of chromatin treatment (Figures 2A–C). When nuclei were subsequently treated with SDS at 65°C, lysis buffers with NP-40 were more preferable. DNA obtained after lysis in hypo- or isotonic conditions without NP-40 was poorer quality than after lysis in the same buffers, but with NP-40 (Figure 2A). When nuclei were subsequently processed with SDS at 37°C, a hypotonic lysis buffer with a maximum content of NP-40 was the only lysis buffer that stabilized DNA during lysis and ensured the absence of its degradation (Figure 2B).

Thus, a combination of hypotonic conditions of the classical buffer of Tolhuis et al. (2002) (10mM Tris-HCl pH 8.0, 10mM NaCl, and 0.2% NP-40) and the amount of detergents proposed by Splinter et al. (2012), van de Werken et al. (2012) (50mM Tris-HCl pH 7.5, 150mM NaCl, 5mM EDTA, 0.5% NP-40, and 1% Triton X-100) makes it possible to preserve DNA integrity under different regimens of chromatin treatment. We additionally removed EDTA from the buffer since the presence of EDTA in the lysis buffer is incompatible with the presence of Mg^2+^ ions, while Mg^2+^ is required for maintaining the RNA structure and stabilizing chromatin (Louwers et al., 2009) and RNA is an integral architectural component of the nucleus, nuclear organelles, and heterochromatin (Caudron-Herger and Rippe, 2012; Hall and Lawrence, 2016; Ding et al., 2019; Michieletto and Gilbert, 2019; Thakur and Henikoff, 2020). The pH of the buffer was slightly shifted from 7.5 to 8.0 because pH 8.0 is most often used for DNA buffers. Thus, our lysis buffer has the following composition: 50mM Tris-HCl, pH 8.0, 10mM NaCl, 0.5% NP-40, and 1% Triton X-100. A similar buffer was used in a very delicate procedure to process mouse oocytes and zygotes in single-nucleus Hi-C (Flyamer et al., 2017). We believe that nuclei are more complete released from the cytoplasm remnants in the presence of a higher content of non-ionic detergents in the buffer. Also the buffer makes it possible to avoid the harsh impact of a Dounce homogenizer, ensuring the intactness of nuclei, while a low ionic strength creates hypotonic conditions in which extraction of the nucleoplasm is most efficient (Méndez and Stillman, 2000; Golov et al., 2015).

It has been reported that a cell lysate should not be viscous since its higher viscosity indicates insufficient cross-linking due to use of FA that is too old (van Berkum and Dekker, 2009). However, we never observed viscous lysates, even when cells were fixed with 1% FA that was stored at +4°C for several months before fixation. According to our observations, viscous lysates rather indicate destruction of nuclei and a release of DNA from them, and DNA always appeared to be degraded in such samples (not shown).

## Nucleoplasm Release and Chromatin Treatment with Heat

SDS and Triton X-100 treatment of nuclei fixed with FA were found to be necessary for any digestion of chromatin to occur (Splinter et al., 2004). The treatment removes the proteins that have not been cross-linked from DNA after fixation and partly denatures cross-linked proteins (Naumova et al., 2012). Accordingly, two fundamentally different regimens of chromatin processing have been proposed for the 3C procedure in the literature. The first regimen, at 37°C, was proposed in the original protocol by Dekker et al. (2002) and was developed in the work by Tolhuis et al. (2002), who increased the durations of consecutive chromatin treatments with SDS and then with Triton X-100 from 10min up to 1h each. Subsequently, as the dilution protocol in the work by Miele et al. (2006), the step of prolonged incubation of nuclei with SDS at 37°C was replaced with a step of short incubation with SDS at 65°C. This step was found to be essential for template generation (Miele et al., 2006) and dramatically increased DNA accessibility, by opening chromatin (Naumova et al., 2012), but, in fact, exactly how the effect of the step is achieved was not demonstrated in either of these two works. Actually, the step was properly studied in a field of plant biology and incubation at 65°C in the presence of SDS was shown to be necessary for inactivating endogenous nucleases (Louwers et al., 2009). The authors found that a nucleosome pattern was obtained when maize nuclei were permeabilized at 37°C with SDS regardless whether RE was added or not, indicating DNA degradation. However, DNA was still intact when isolated from an aliquot taken before the SDS incubation. The authors concluded that degradation occurred during incubation of nuclei with SDS at 37°C. Degradation was completely prevented when plant nuclei were first incubated at 65°C for 60min before adding SDS (Louwers et al., 2009).

We also studied the effect of this step and found that heating chromatin at 65°C results in a some extent of DNA preservation during subsequent isolation (Figure 2D). This is supported by predominant DNA degradation that was observed after processing chromatin with SDS at 37°C (Figure 2B)and eradicated by chromatin treatment at 65°C (Figure 2A). The results indicate that DNA degradation in S2 cells can also be due to the presence of endogenous nucleases, as it was previously described for plants (Louwers et al., 2009), while the enzymes are at least partly inactivated at 65°C.

As follows from Figure 2A, lysis under hypotonic conditions appears to be more efficient in terms of maintaining DNA integrity since the nucleoplasm, containing different nucleases with DNase I activity (Yang, 2011), is efficiently released in these conditions (Méndez and Stillman, 2000; Golov et al., 2015). We assume that a structural disruption of fixed nuclei may precede DNA degradation and is due to long-term treatment at 65°C in the presence of SDS. Therefore, after performing lysis in isotonic conditions, we titrated the duration of exposing nuclei to heat at 65°C and found that incubation of fixed nuclei for more than 40min results in DNA degradation, presumably indicating disintegration of the nuclei and a release of nucleases (Figure 2E). We concluded that heating nuclei at 65°C should not exceed 40min in case of S2 cells.

Thus, these data suggest that, on the one hand, heat treatment of fixed nuclei in the presence of SDS at 65°C partly reverses the cross-links and inactivates the remaining nucleases of a cytoplasmic origin and, in part, nuclear nucleases. On the other hand, the treatment leads to disintegration of the fixed nuclei and the release of residual nuclear nucleases, causing subsequent DNA degradation (see also the section Reversion of Cross-links and Isolation of the 3C Library section). From this point on, we used a hypotonic lysis buffer.

**Figure 3:**
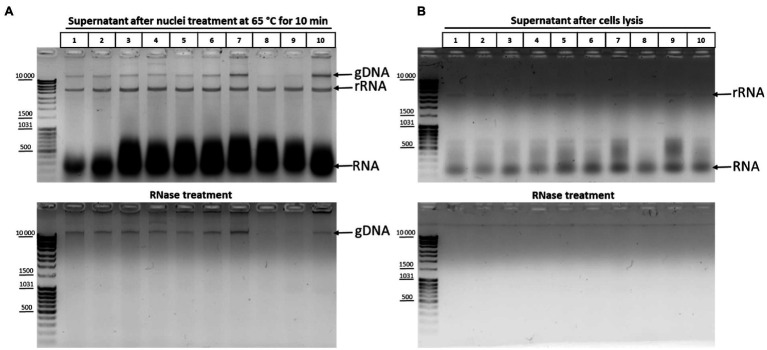
DNA and RNA release from nuclei upon their treatment with heat in the presence of SDS. **(A)** Chromatin treatment at 65°C causes DNA release from nuclei. Сells were fixed, quenched, and washed after quenching with 1X PBS as in Figures 2A–C and then lysed in hypotonic lysis buffer #11 (see Table 1), incubated on ice for 15min, washed once with ice-cold 1X PBS, and resuspended in 1X RB for DpnII. Then, SDS was added up to 0.3% and the nuclei were treated with heat at 65°C for 10min+37°C for 50min. After that, Triton X-100 was added up to 1.8% and the nuclei were incubated at 37°C for 1h. Thereafter, the samples were separated into the supernatant (100μl) and the pellet and 500μl of EB was added to the supernatant fractions (lanes 1–10, 10 replicates of the experiment). Then, the cross-links were reversed and the DNA was extracted, precipitated, dissolved, and treated with bovine RNase A (lower panel) as in Figures 2A–C or left untreated (upper panel). The amount of the DNA subjected to electrophoresis was 2μl. gDNA – genomic DNA, rRNA – processed high-molecular-weight RNA, and RNA – low-molecular-weight RNA (tRNA + degraded mRNA). **(B)** The content of nucleic acids in the supernatant after the stage of cell lysis. Сells were lysed on ice as in **A** and the samples were separated into the supernatant containing cytoplasm (100μl) and the pellet fractions. EB was added to the supernatant fractions as in **A**. Then, the cross-links were reversed and the DNA was extracted, precipitated, dissolved, and treated with bovine RNase A (lower panel) or left untreated (upper panel) and subjected to electrophoresis as in Figures 2A–C. All other designations are as in **A**.

The effect of DNA degradation depending on the time of incubation at 65°C (Figure 2E) was unexpected but fits into the standard 3C protocol schemes. The time of chromatin processing at 65°C usually does not exceed 5–10min in different types of 3C protocols (Miele et al., 2006; Kalhor et al., 2012; Rao et al., 2014). The results of the experiment (Figure 2E) imply that a brief chromatin treatment at 65°C already damage nuclei. This damage might partly release genomic DNA into solution. To study this issue, we analyzed 10 independent samples wherein chromatin was heated for 10min at 65°C in the presence of 0.3% SDS. After heating, the samples were divided into the supernatant and pellet fractions and the supernatant fractions were analyzed. Release of the genomic DNA from nuclei into the supernatant (as well as a release of processed ribosomal RNA) was clearly detectable in 8 out of 10 samples after nuclei were heated at 65°C for 10min, apparently indicating damage to the fixed nuclei (Figure 3A).

To exclude that the nuclei were damaged at the previous stage of the procedure, we carried out the same experiment at the cell lysis stage. Analysis of the supernatant after cell lysis showed that only rRNA and low-molecular-weight RNA came out into solution during cell lysis and that there was no release of genomic DNA into solution (Figure 3B).

Thus, even a short incubation at 65°C apparently leads to damage to nuclear structures and a partial release of genomic DNA from nuclei into solution.

**Figure 4:**
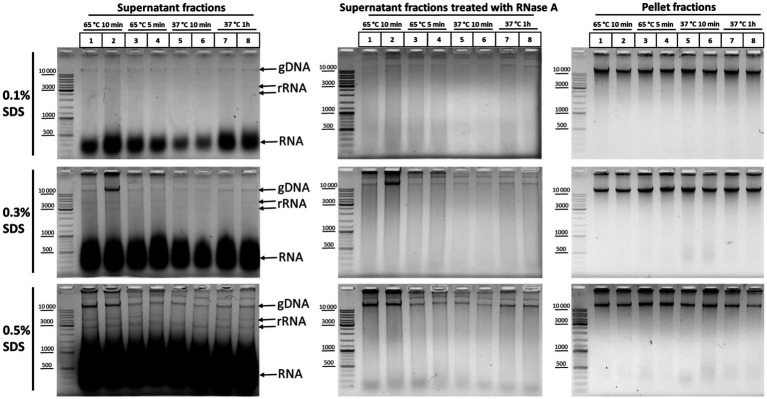
DNA and RNA release from nuclei at different SDS concentrations and under different nuclei treatment regimens. Сells were fixed, quenched, washed after quenching with 1X PBS as in Figures 2A–C, and then lysed in hypotonic lysis buffer #11 (see Table 2). Cells were incubated on ice for 15min and nuclei were washed once with the lysis buffer and then resuspended in water supplemented with the appropriate amount of SDS (0.1, 0.3, or 0.5%). Then, the nuclei were treated with heat in the following conditions: 65°C for 10min (lanes 1, 2), 65°C for 5min (lanes 3, 4), 37°C for 10min (lanes 5, 6), and 37°C for 1h (lanes 7, 8). Triton X-100 was added up to 1.8% and nuclei were incubated at 37°C for 15min. Thereafter, the samples were separated into the supernatant (250μl) and the pellet and 500μl of EB was added to the supernatant fractions. Then, the cross-links were reversed at 56°C O/N and DNA was extracted, precipitated, dissolved, and treated with bovine RNase A (middle row of panels from top to bottom) as in Figures 2A–C or left untreated (left row of panels from top to bottom). The pellet fractions (right row of panels from top to bottom) were processed further as follows: Nuclei were washed with 1X RB for DpnII buffer and incubated in 1X RB at 37°C for 1h with agitation (restriction reaction imitation). After that, nuclei were washed with 1X T4 DNA ligase buffer (50mM Tris-HCl pH рН7.5, 10mM MgCl_2_, 1mM ATP, and 10mM DTT) three times and incubated at 16°C for 30min and at 22°C for 30min in 1X T4 DNA ligase buffer (ligation reaction imitation). Then, 500μl of EB was added and the pellet fractions were processed as for the supernatant fractions. The amount of the DNA subjected to electrophoresis was 2μl for supernatant fractions and 1μl for pellet fractions. Upper row of pictures from left to right – treatment with 0.1% SDS; middle row of pictures – 0.3% SDS; and the bottom row of pictures – 0.5% SDS. All other designations were as in Figure 3A. Two replicates of each experimental condition were carried out. The exposure time for all gels is the same.

In order to understand which particular processing regimens of nuclei are the most harmful for nuclear integrity, we studied the release of DNA and rRNA into solution at different concentrations of SDS upon treatment of nuclei at 65°C for 10 and 5min, as well as at 37°C for 10min and 1h. We found that, at 0.1% SDS, all processing regimens were apparently benign for nuclei and the release of DNA and RNA into solution was minimal (Figure 4, upper row of panels, from left to right). At 0.3% SDS, regimens with 65°C for 5min, 37°C for 10min, and 37°C for 1h appeared to be equivalent in terms of maintaining nuclear integrity and provided minimal DNA release, but treatment at 65°C for 10min provoked a noticeable DNA release in one replicate (Figure 4, middle row of panels, from left to right). Besides, a more pronounced release of low-molecular-weight RNA from nuclei was observed at 0.3% SDS compared with 0.1% SDS. At 0.5% SDS, treatment at 65°C for 10min provoked a noticeable DNA release in both replicates and a more pronounced DNA release was observed with all other regimens (Figure 4, bottom row of panels, from left to right). Besides, a huge amount of low-molecular-weight RNA and an appreciable amount high-molecular-weight RNA were released from nuclei into solution in the presence of 0.5% SDS.

We concluded that 0.3% is the maximum possible SDS concentration that does not cause nuclear damage. At this SDS concentration, it is possible to process nuclei at 65°C for 5min to inactivate the residual nuclease activity without significantly compromising their integrity. Pronounced solubilization of histones from the nuclear fraction into the solution was also observed at this concentration (Gavrilov, 2016). However, the 0.1% SDS concentration appears to be the most sparing and makes it possible to preserve the maximum amount of nuclear RNA.

A scenario is also likely that the treatment of nuclei with heat and SDS does not cause partial damage to all nuclei, but rather a complete disintegration of some of the nuclei occurs. However, our observations testify against this scenario; i.e., we did not observe any decrease in the mass of the nuclear pellet even when nuclei were treated at 65°C for 10min in the presence of 0.5% SDS. We noticed that the higher SDS concentration, the more transparent was the pellet of nuclear. When nuclei were treated with 0.1% SDS, the pellet of S2 nuclei was light gray. When nuclei were treated with 0.5% SDS, the pellet became “glassy” and difficult to work with at subsequent stages. A nuclear pellet obtained after treatment with 0.3% SDS had an intermediate transparency. We also noticed that cell nuclei acquire the same “glassy” appearance when the RE is inactivated after the restriction reaction in the presence of 1.3–1.6% SDS in the dilution protocol.

In addition, we found that not only high-molecular-weight DNA was detectable in solution after RNase treatment of the supernatant fractions, but also a significant amount of DNA molecules of different lengths (a DNA smear from more than 10,000 to 100bp; Figure 4, middle row of panels, from top to bottom). The severity of chromatin treatment correlated with the intensity of the smear, which was minimal in the case of the regimen of 37°C for 10min. The finding, together with the intactness of the high-molecular-weight DNA band, indicates that this fraction is a result of the processing of nuclei with SDS, rather than DNA degradation in the process of DNA isolation/cross-links reversion. We do not know about the nature of this DNA fraction, but it is possible that this DNA may contribute to the elevated frequency of spurious contacts due to random ligation in dilute solution and might represent a source of experimental noise as described in Rao et al. (2014), Nagano et al. (2015b), Downes et al. (2021). Our data agree with the observations by Downes et al. (2021), who showed by separating the *in situ* 3C sample into intact nuclei and soluble DNA that ~25% of *in situ* 3C libraries come from disrupted nuclei.

Thus, taken together, the results suggest that the longer the incubation of nuclei at a higher temperature and the greater the SDS concentration used to extract nuclear proteins, the more genomic DNA and RNA passed into the supernatant fraction, indicating progressive damage to the nuclear structure with the increase in temperature and SDS concentration. Incubation at 37°C provides milder conditions of chromatin treatment. On the other hand, heat treatment of nuclei at 65°C is necessary to inactivate nucleases and may be useful for better DNA preservation at subsequent stages. However, the duration of chromatin treatment with heat, as well as the SDS concentration, must be kept as low as possible to preserve the integrity of nuclei.

## Restriction of DNA in Nuclei

In this part, we studied RE digestion of chromatin in nuclei. Protein complexes cross-linked to DNA may block restriction sites and reduce the efficiency of restriction digestion (Naumova et al., 2012). In turn, the efficiency of protein complex cross-linking can be influenced by the amount of FA used for fixation. Besides, the SDS/Triton X-100 ratio in the restriction reaction mixture, the duration of digestion and concentration of RE, and the conditions of chromatin treatment before chromatin digestion may exert an effect on the digestion efficiency. It was proposed to optimize the digestion efficiency by varying the SDS/Triton X-100 amounts before digestion, increasing Triton X-100 upon restriction digestion, and lowering the FA concentration (Hagège et al., 2007; Stadhouders et al., 2013). Since we selected the conditions for washing of nuclei from SDS/Triton X-100 (Supplementary Figure S1C), we therefore focused on the influence of chromatin treatment conditions and FA concentration on the restriction digestion efficiency.

**Figure 5:**
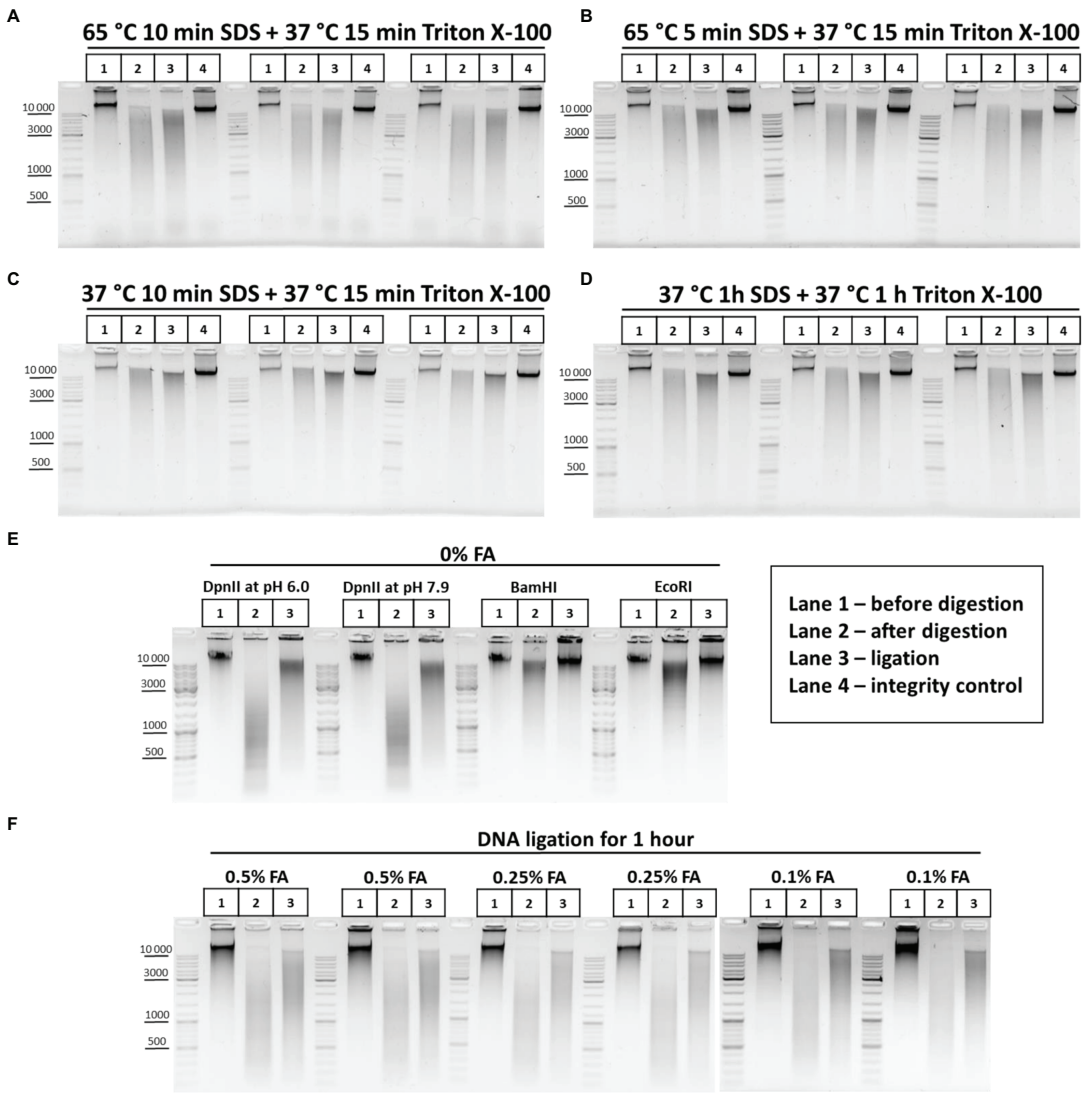
Restriction digestion of DNA in nuclei and in solution. **(A**–**D)** Endonuclease digestion of the chromatin in nuclei after different regimens of chromatin treatment. Сells were fixed, quenched, washed after quenching with 1X PBS as in Figures 2A–C and then lysed, incubated in lysis buffer, and washed as in Supplementary Figure S1A. Then, nuclei were resuspended in autoclaved water supplemented with 0.3% SDS and were treated with the SDS and subsequently with Triton X-100 (1.8% final) in the following conditions, as indicated in the figure: (A) 65°C for 10min with SDS+37°C for 15min with SDS/Triton X-100; (B) 65°C for 5min with SDS+37°C for 15min with SDS/Triton X-100; (C) 37°C for 10min with SDS+37°C for 15min with SDS/Triton X-100; and (D) 37°C for 1h with SDS+37°C for 1h with SDS/Triton X-100. Then, nuclei were centrifuged, supernatant containing SDS/Triton-X100 was removed and nuclei were washed three times with 1X RB (DpnII buffer), and then resuspended in 1X RB and incubated with 2U/μl of DpnII for 3h at 37°C with agitation. Then, nuclei were washed three times with 1X T4 DNA ligase buffer, resuspended in 1X T4 DNA ligase buffer, and incubated with 0.25U/μl of T4 DNA ligase (Sibenzyme) for 30min at 16°C and for 30min at 22°C with agitation. Aliquots of 1/10 of the sample volume were taken after chromatin treatment with SDS/Triton X-100, followed by washing of the nuclei with 1X RB (Lane 1 – undigested chromatin, chromatin integrity control before RE digestion, control #1) and, after restriction reaction, followed by washing with 1X T4 DNA ligase buffer (Lane 2 – digested chromatin, chromatin restriction control after RE digestion, control #2). The volume of samples was adjusted, with 1X T4 DNA ligase buffer, to 250μl and PrK, SDS and EDTA were added, as in Supplementary Figure S1A, to the control #1, control #2, and ligation (lane 3) and control #3 (lane 4 – a control of DNA integrity throughout all stages of the procedure – sample that went through the entire procedure without addition of RE and T4 DNA ligase). Cross-links were reversed as in Supplementary Figure S1A and the DNA was extracted, precipitated as in Figures 2A–C, dissolved in 25μl for control #1 and #2, in 20μl for ligation and in 25μl for control #3, and then treated with bovine RNase A as in Figures 2A–C. 10 μl of dissolved DNA was subjected to electrophoresis for control #1 and #2, as was 1μl for ligation and control #3. Three replicates of each experimental condition were carried out. **(E)** Patterns of the genomic DNA digestion with 4-bp and 6-bp cutters and ligated in solution. 100mg of S2 cells was harvested, washed with 1X PBS, and resuspended in 1ml of EB. Cells were incubated at 56°C for 30min and the DNA was extracted, precipitated (w/o glycogen), washed as in Figures 2A–C, dissolved in 150μl of Tris-HCl pH 7.9, treated with 50U of RNase I (Thermo) at RT for 30min, purified using 1.5X AMPure XP beads (see purification of 3C library on magnetic beads), and eluted with 150μl of 10mM Tris-HCl pH 7.9. 1μl of the DNA was digested with 10U of Res, indicated above the picture, in the following buffers: DpnII pH 6.0, NEB3 pH 7.9, and BamHI and EcoRI, respectively. After digestion, the REs were heat inactivated and DNA was precipitated, washed with ethanol as in Figures 2A–C and subjected to electrophoresis or ligated in solution with 0.05U/μl of T4 DNA ligase (Sybenzyme) for 30min at 16°C and 30min at 22°C with agitation, purified using AMPure XP beads (the elution was implemented using 10mM Tris-HCl pH 7.9 at RT), and then subjected to electrophoresis. Lane 1 – non-digested DNA; Lane 2 – digested in solution DNA; and Lane 3 – DNA ligated in solution after digestion. One of the three replicated experiments is shown. **(F)** Concentration of the fixing agent below 0.5% improves chromatin digestion. Сells were fixed with different concentrations of FA (indicated above the pictures) in 1X PBS at RT for 10min, quenched, washed after quenching with 1X PBS as in Figures 2A–C and then lysed, incubated in lysis buffer, and washed as in Supplementary Figure S1A. Then, nuclei were resuspended in autoclaved water supplemented with 0.3% SDS, incubated at 65°C for 5min and then Triton X-100 was added up to 1.8% and nuclei were incubated at 37°C for 15min. Then, nuclei were washed with 1X RB three times and the RE digestion of the chromatin, washing of nuclei and DNA ligation in nuclei were carried out as in **A**–**D**. Control aliquots were taken as in **A**–**D**. The volume of samples was adjusted with 1X T4 DNA ligase buffer to 250μl and PrK, SDS, and EDTA were added. The cross-links were reversed as in Supplementary Figure S1A and the DNA was extracted, precipitated as in Figures 2A-C, dissolved as in **A**–**D**, treated with bovine RNase A as in Figures 2A–C, and subjected to electrophoresis as in **A**–**D**. The designation of lanes is as in **A**–**D**. Two replicates of each experimental condition were carried out.

First, to understand how the chromatin processing conditions affect the efficiency of DNA digestion in nuclei, we processed nuclei with SDS in two regimens, at 65 and 37°C, and then sequestrated SDS with Triton X-100. The DNA restriction pattern was not found to change depending on the temperature and duration of chromatin treatment (Figures 5A–D). Instead, unexpectedly, we observed more efficient ligation (denser and sharper at the top of the smear after DNA ligation) when chromatin was processed with SDS at 37°C (Figure 5, compare panels A and B with C and D).

Thus, more stringent conditions of chromatin treatment do not result in more efficient digestion of chromatin with RE but may instead influence the ligation efficiency.

Next, we investigated how the FA concentration affects the efficiency of chromatin digestion in nuclei. The formation of DNA fragments of higher molecular weights (MWs) is indicative of incomplete fragmentation of chromatin after its restriction digestion with a 4-bp cutter in nuclei (Figures 5A–D). This pattern corresponds to the pattern of DNA digested in solution with a 6-bp cutter (Figure 5E) rather than of DNA digested in solution with a 4-bp cutter (Figure 5E). Hence, we concluded that chromatin was not fully digested in nuclei. There are indications that the FA concentration may be directly related to the efficiency of chromatin digestion (Splinter et al., 2004; Dekker, 2007; Comet et al., 2011; van de Werken et al., 2012). The 1% FA concentration, which we used in experiments illustrated in Figures 5A–D, might be too high for efficient digestion. Therefore, we lowered the FA concentration to 0.5% and observed much more efficient digestion of cross-linked chromatin with DpnII (Figure 5F). The restriction pattern obtained at 0.5% FA was more similar to that observed after restriction in solution (Figure 5E) and did not shift down with a decrease in the FA concentration to 0.25% or even to 0.1% (Figure 5F).

Moreover, we noticed that the results related to chromatin accessibility to a RE may depend on the FA source. For example, a 1% FA solution prepared from PFA powder provides a higher fixation strength than a similar solution prepared from a 37% ready-to-use commercial solution. We estimate that 0.5% FA made from PFA and 1% FA made from a 37% commercial solution show comparable fixation efficiencies.

Other important issues are the duration of digestion and the RE concentration in the restriction reaction. The most common incubation time with a RE is 12–16h (overnight incubation; Louwers et al., 2009; Nagano et al., 2013, 2015b, 2017; Flyamer et al., 2017). Digestion for 2–4h was also suggested (Rao et al., 2014; Golov et al., 2020; Vermeulen et al., 2020; Ulianov et al., 2021). We determined that desired result is achieved within 3h, although overnight incubation is convenient (Figure 5F). Regarding the RE concentration in the restriction reaction, a concentration of 2U/μl is sufficient for efficient digestion of chromatin, in the case of using 10mg of starting material as described in Figures 2A–C. The DpnII concentration we used was slightly higher than in recent works (0.66–1.66U/μl; Golov et al., 2020; Vermeulen et al., 2020; Ulianov et al., 2021) and more similar to that used to digest yeast chromatin (2.07U/μl; Schalbetter et al., 2019).

Thus, our data suggest that the efficiency of chromatin digestion depends mostly on the cross-linking agent concentration and is independent of the conditions of SDS/Triton X-100 chromatin treatment before digestion or the digestion time. Cells fixation with 0.5% FA for 10min is sufficient for efficient digestion of chromatin with DpnII for 3h at a concentration of RE of 2U/μl.

## Ligation of DNA in Nuclei

**Figure 6:**
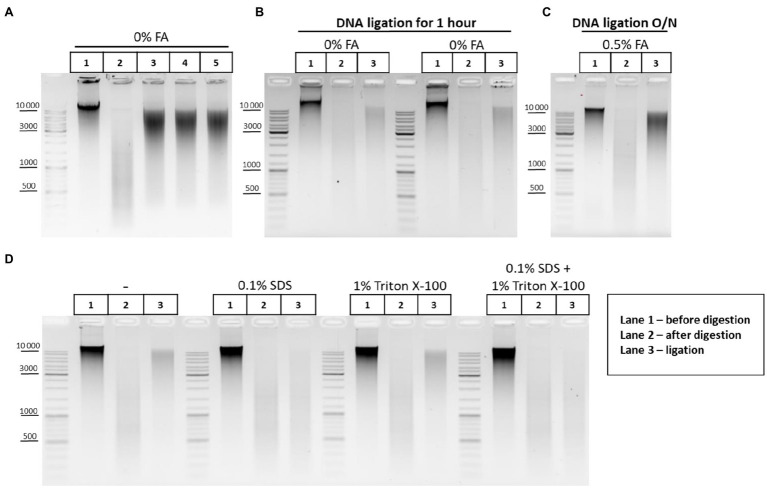
DNA ligation in nuclei and in solution. **(A)** Patterns of the genomic DNA digested with DpnII and ligated in solution with different concentrations of T4 DNA ligase. Genomic DNA was isolated (lane 1) and cut with DpnII as in Figure 5E. Then, RE was heat inactivated and DNA was purified on AMPure XP beads and subjected to electrophoresis (lane 2) or ligated for 1h as in Figure 5E in a reaction volume of 20μl using 1μl of commercially available preparation of T4 DNA ligases in the following concentrations: lane 3–5 Weiss U/μl (Thermo, #EL0014), lane 4–1 Weiss U/μl (Sibenzyme, #E320), and lane 5 – high concentrated ligase of 10 Weiss U/μl (Sibenzyme, #E330). After ligation, the DNA was again purified using AMPure XP beads and subjected to electrophoresis. One of the three replicate experiments is shown. **(B)** Pattern of ligation of uncross-linked chromatin in 1h. Сells were left unfixed (0% FA), were washed with 1X PBS at RT, and were lysed, incubated in lysis buffer, and washed as in Supplementary Figure S1A. Then, nuclei were treated with SDS and Triton X-100 as in Figure 5F. Nuclei were then washed with 1X RB and after that RE digestion of the chromatin and DNA ligation in the nuclei were implemented as in Figures 5A–D. Control aliquots were taken as in Figures 5A–D. The volume of samples was adjusted, with 1X T4 DNA ligase buffer, to 250μl and then PrK, SDS, and EDTA were added to the controls and to the ligation and the cross-links were reversed as in Supplementary Figure S1A. After this, DNA was extracted, precipitated as in Figures 2A–C, dissolved as in Figures 5A–D, treated with bovine RNase A as in Figures 2A–C, and subjected to electrophoresis as in Figures 5A–D. The designations of lanes are as in **A** two replicates of the experiment were carried out. **(C)** Pattern of ligation of cross-linked chromatin overnight. The experiment was carried out as in **B**, except that cells were fixed, quenched, washed after quenching with 1X PBS as in Figures 2A–C, and ligation was implemented as in Figures 5A–D (with the exception of heat treatment being performed O/N at 16°C). The designations of lanes are as in **A**. One of the three replicate experiments is shown. **(D)** Influence of the SDS and Triton X-100, and their combined influence, on ligation efficiency. The experiment was carried out as in **B**, except that the cells were fixed with 0.5% FA in 1X PBS at RT for 10min, quenched, and washed after quenching with 1X PBS as in Figures 2A–C. The ligation was implemented as in Figures 6A–D, except that DNA ligation was performed O/N at 16°C under the following conditions: without SDS and without Triton X-100 (first panel); in the presence of 0.1% SDS (second panel); in the presence of 1% Triton X-100 (third panel); and in the presence of both 0.1% SDS and 1% Triton X-100 (fourth panel). Then, control aliquots were taken as in Figures 6A–D and samples were processed further, as in **B**. The designation of lanes is as in **A**. One of the three replicate experiments is shown.

Before studying the peculiarities of ligation in nuclei, we checked how exactly T4 DNA ligase concentration affects the ligation efficiency. Activities of T4 DNA ligases were investigated in solution using genomic DNA cut with DpnII. The DNA ligation pattern in solution was not found to vary when T4 DNA ligase was used at 1, 5, or 10U per reaction (Figure 6A). However, highly concentrated T4 DNA ligase (10U/μl) is convenient to use since it prevents large amounts of glycerol from entering the reaction.

The pattern obtained after DNA digestion and ligation in solution (Figure 5E) differed from ligation patterns obtained after chromatin ligation in nuclei (Figures 5A–D,F). We assumed that DNA incubation at 16°C for 30min followed by 22°C for 30min (Figures 5A–D,F) may be insufficient for efficient ligation of cross-linked chromatin in nuclei. We therefore performed the experiments where non-cross-linked chromatin was ligated using the same incubation time (1h) or cross-linked chromatin was ligated for incubation time extended from 1h to overnight. It was observed that uncross-linked chromatin was more readily ligated in nuclei than cross-linked chromatin within 1h (compare lane 3 in Figures 5A–D,F and lane 3 in Figure 6B). The prolongation of the ligation time for cross-linked chromatin from 1h to overnight had a positive effect on the ligation pattern (made the shmear to shift up; compare lane 3 in Figures 5A–D,F and lane 3 in Figure 6C).

Thus, cross-linking apparently imposes certain spatial restrictions on the rate of chromatin ligation in nuclei and these can be overcome by a longer ligation duration.

Since SDS dramatically reduces the ligation efficiency (Louwers et al., 2009), chromatin ligation is usually performed in the 3C procedure after strong dilution of the restriction reaction mixture containing a high amount of SDS sequestered with Triton X-100. In this case, T4 DNA ligase is added to a buffer containing SDS diluted to 0.1% and sequestered with 1% of Triton X-100 (Dekker et al., 2002; Tolhuis et al., 2002; Lieberman-Aiden et al., 2009; Comet et al., 2011; Stadhouders et al., 2013; Vermeulen et al., 2020). However, as in the case of restriction digestion, the ligation reaction in the presence of 0.1% SDS and 1% Triton X-100 may be far from optimal. Therefore, we investigated the issue of whether the presence of 0.1% SDS and 1% Triton X-100 in the ligation reaction affects the efficiency of ligation in nuclei. We found that 0.1% SDS present in the reaction mixture alone, or surprisingly, in combination with 1% Triton X-100 (fresh stock and fresh working solution were prepared) negatively affects the ligation efficiency, whereas 1% Triton X-100 alone does not affect the ligation pattern (Figure 6D).

Thus, the washing of nuclei with the 1X T4 DNA ligase buffer after restriction digestion as proposed by Flyamer et al. (2017), Golov et al. (2020) helps to ensure efficient ligation by washing out SDS.

When the ligation reaction is carried out in the presence of 0.1% SDS and 1% Triton X-100, then complete sequestration of SDS by Triton X-100 is an important factor, since trace amounts of SDS will inhibit T4 DNA ligase. It was proposed to prepare a new Triton X-100 working solution every 1–2months, since an old Triton X-100 solution has a notable negative effect on the digestion efficiency, probably due to inefficient sequestration of SDS with Triton X-100 decayed by light (Louwers et al., 2009). We did not observe any difference between a freshly prepared stock solution (20%) and an old one, which was kept protected from light at +4°C for at least 1year (not shown). T4 DNA ligase worked inefficiently in nuclei in the presence of SDS regardless of whether the new (Figure 6D) or old (not shown) Triton X-100 solution was used for sequestration.

It was shown that 0.1% SDS in combination with 1% Triton X-100 does not reduce the efficiency of DNA ligation in solution (plasmid DNA digested with a 6-bp cutter; Gavrilov et al., 2013a). However, 0.1% SDS in combination with 1% Triton X-100 does affect nuclear ligation (chromatin digested with a 4-bp cutter) according to our results. This effect may be due to the different times periods required for completing the reactions. Ligation of plasmid DNA fragments in solution takes place within just 10min (Gavrilov et al., 2013a), whereas efficient ligation of fixed chromatin in nuclei requires at least several hours of incubation according to our results (compare Figure 5F lane 3 and Figure 6C lane 3). During this long incubation time, T4 DNA ligase is possibly inactivated by 0.1% SDS. Besides, 4-bp protruding ends are generally less efficiently ligated even in solution as it follows from the Figure 6E.

The efficiency of restriction digestion and ligation can be determined by performing a PCR spanning a specific genomic restriction site (Gavrilov, 2016). In order to quantitatively estimate the efficiency of ligation (regeneration of the DpnII site) in our conditions, the amount of the intact site (uncut and religated) was measured before and after ligation in the *RpII* locus by PCR-stop analysis (Comet et al., 2011). Loss of the amplicon signal after RE treatment is indicative of digestion efficiency (Belton et al., 2012). An increase in amplicon signal after ligation above the level of the uncut site indicates a ligation event, suggestion regeneration of the intact original restriction site (Gavrilov, 2016).

The experimental design of the system used to estimate the digestion and ligation efficiency in the *RpII* locus is shown in Supplementary Figures S2A,B. Before experiments, we validated the presence of a DpnII restriction site at the required position of the *RpII* locus by sequencing (Supplementary Figure S2C) and then optimized the Taq-man PCR conditions to achieve maximum sensitivity (Supplementary Figure S2D; Supplementary Table S2).

To estimate the digestion efficiency, one-third of the sample after overnight digestion (10–12 mln of starting nuclei) was recommended to take (Louwers et al., 2009), but our experience showed that up to half of the digested sample is required to use to reliably and conveniently measure the efficiency of digestion for 12mln of starting cells as described in Figures 2A–C.

**Figure 7:**
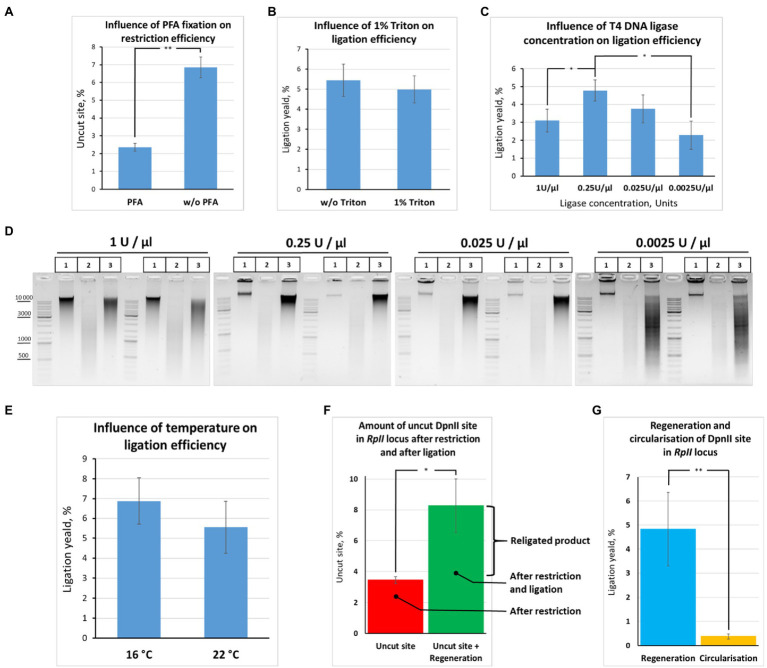
Quantification of restriction digestion and ligation efficiency depending on reaction conditions. **(A)** Cell fixation enables efficient chromatin digestion. The experiment with unfixed cells was performed as in Figure 6B, except that ligation O/N was at 16°C. The experiment with fixed cells was carried out as in Figure 6B, except that cells were fixed with 0.5% FA, quenched, washed after quenching with 1X PBS as in Figures 2A–C, and ligation O/N was at 16°C. Control aliquots were taken as in Figures 5A–D and samples were processed further as in Figure 6B. The graph shows the frequency of intact (uncut) restriction site in the samples fixed with FA or non-fixed. The proportion of the uncut site was determined using PCR-stop analysis as described in Figures 8A,B. Error bars indicate SDs of four technical PCR measurements from at least three independent biological replicates of 3C library. Asterisks indicate significance levels: ^**^p<0.001, *n*=22. **(B)** Triton X-100 does not influence ligation efficiency. The experiment was carried out as in **(A)**. The ligation yield was calculated as described in Supplementary Figures S2A,B. No statistically significant difference between the groups was found (*p*<0.5, *n*=20). **(C,D)** High T4 DNA ligase concentration decreases ligation efficiency. The experiments were carried out as in **A**, except that ligation was performed at different T4 DNA ligase concentrations, as indicated, O/N at 16°C. The ligation yield was calculated as described in Supplementary Figures S2A,B. Error bars indicate SDs of four technical PCR measurements from at least three independent biological replicates of 3C library. Statistically significant differences between groups are indicated with asterisks: ^*^p<0.05. The representative experiment presented under **D** was carried out in two replicates. The designation of lanes in **D** are as in Figures 5A–D. **(E)** Ligation efficiency does not significantly differ at 16°С and 22°С. The experiment was carried out as in **A**. The ligation yield was calculated as described in Supplementary Figures S2A,B. No statistically significant difference between the groups was found (*p*<0.25, *n*=8). **(F,G)** Quantitative values reflecting the effectiveness of the 3C procedure at the *RpII* site. The experiments were implemented as in **A**. The amount of intact (uncut or religated) DpnII site, the ligation yield, and the amount of circularized product were estimated as described in Supplementary Figures S2A,B. Asterisks indicate significance levels: for **F** ^*^p<0.05, *n*=24; for **G** ^**^p<0.001, *n*=20.

The first thing that we found was that fixed chromatin treated with SDS/Triton X-100 is much more readily digested than non-fixed chromatin treated with SDS/Triton X-100 in the same way (Figure 7A). We assume that cell fixation preserves the nuclear architecture and nuclear pores in particular and thereby contributes to a more complete release of histones and other nuclear proteins from the nuclei upon their SDS/Triton X-100 treatment, as it generally anticipated for the 3C procedure (Dekker et al., 2002) and was demonstrated in Gavrilov (2016). A possible alternative explanation is that, without cross-linking, cells/nuclei are broken during SDS treatment (either at 37°C or 65°C), which leads to aggregation of the nuclei (DNA in aggregated nuclei is not digestible by REs). Thus, fixed chromatin appears to be more permissive to restriction digestion than non-fixed chromatin.

Second, we did not find any ligation yield after digestion and ligation of non-fixed chromatin; no regeneration of the DpnII site occurred in non-fixed chromatin against the background of uncut product (compare Figures 7A,F,G). The data are consistent with early observations by Dekker et al. (2002) and Gavrilov and Razin (2008), who noted that the ligation yield is very low in non-fixed yeast and mammalian cells, respectively, not exceeding 5% of the amount of ligation in fixed cells. The data agree with later observations by Belaghzal et al. (2017) that leaving out the cross-linking step leads to dramatic loss of detected contacts and the inability to reconstruct the chromatin conformation beyond a few kb.

Third, we observed that the presence of 1% Triton X-100 in the ligation reaction did not exert a significant effect on the ligation efficiency (Figure 7B). The finding was consistent with electrophoresis data (Figure 6D). Thus, Triton Х-100 added up to 1% does not inhibit ligation in nuclei. Moreover, we observed that the ligation efficiency may be improved in the presence of Triton X-100 in the ligation reaction since some amount of Triton X-100 prevents adhesion of nuclei to the tube walls. To prevent adhesion of nuclei, 0.1% Triton X-100 is sufficient.

Further, we quantified how the T4 DNA ligase concentration and the temperature affect the efficiency of DNA ligation in nuclei. We found that ligation was most efficient at T4 DNA ligase concentrations ranging from 0.25 to 0.025U/μl. We did not find significant differences in ligation efficiency between the two concentrations, while lowering the ligase concentration to 0.0025U/μl led to a significant decrease in ligation efficiency (Figures 7C,D). Unexpectedly, increasing the T4 DNA ligase concentration in the reaction from 0.25 to 1U/μl decreased the ligation efficiency (Figures 7C,D).

We assume that an increase in T4 DNA ligase concentration leads to rapid consumption of the ATP pool and that this might be critical during the long incubation time of the reaction. Thus, the reaction of chromatin ligation in nuclei should be neither overloaded with T4 DNA ligase nor lacking it. Our results are in good agreement with the data of other authors. For example, the final concentrations of T4 DNA ligase were 0.0012, 0.001, and 0.006 Weiss U/μl in the articles (Lieberman-Aiden et al., 2009; Naumova et al., 2013; Falk et al., 2019), respectively, where the Dekker team’s protocol was used. The concentration of T4 DNA ligase was also not high in other classical works and protocols. For example, the concentrations were 0.006, 0.008, and 0.014 Weiss U/μl in (Stadhouders et al., 2013; Rao et al., 2014; Nagano et al., 2015b), the concentrations were 0.006, 0.008 and 0.014 Weiss U/μl, respectively, and 0.013 Weiss U/μl in Splinter et al. (2012), van de Werken et al. (2012) and 0.02 and 0.024 in Comet et al. (2011) for 3C and H3C, respectively (Cavalli team’s protocol). TThe range of the most efficient T4 DNA ligase concentrations (0.25-0.025 U/μl) that we found for S2 cells is more consistent with the T4 DNA ligase concentration described for *Drosophila* tissues (from adults, pupae, or embryos; Comet et al., 2011). As for the temperature, we found no significant differences in ligation efficiency between 16 and (Figure 7E).

Next, to quantify the ligation efficiency, we measured the amounts of the uncut site and ligated product (target ligation, regeneration of the original restriction site, or ligation yield). The amounts of the uncut site and religated product were estimated at 3.4±0.2 and 8.3±1.7%, respectively (Figure 7F). The average range of variation between technical replicates was only ±0.4% after determining the amount of the uncut site, while it was ±1.5% after determining the ligation yield, the two values differing significantly (*p*<0.05, *N*=20). Moreover, there was no significant correlation between the amount of the uncut site and the ligation yield either in the presence of 1% Triton X-100 (*r*=0.49, *p*<0.15, *N*=10) or in the absence of Triton X-100 (*r*=0.333, *p*<0.35, *N*=10).

We concluded that our experimental conditions make it possible to achieve efficient chromatin digestion with DpnII and to detect the ligation products at an acceptable level, above the background of non-cleaved DNA. At the same time, a larger data variation observed after ligation than after digestion is possibly a reflection of the fact that ligation of target DNA ends is a rare event in the 3C procedure (Gavrilov et al., 2013a; Gavrilov, 2016), thus, requiring prolong incubation in the case of fixed nuclei (Figures 5A–D,F, Figures 6B,C) and being therefore statistically more variable according to our results.

The efficiency of DNA digestion should be as high as possible, preferably higher than 80% (Louwers et al., 2009; Naumova et al., 2012; van de Werken et al., 2012) and may vary between samples and cell types used (Splinter et al., 2012).

Several works were performed to measure the frequencies of restriction site regeneration (ligation yield) and the percentage of uncut site copies (van de Werken et al., 2012; Gavrilov, 2016). We compared our results with the results from these works (Table 3).

**Table 3:** Amounts of uncut site and the ligation yield in different type of protocols.

	Protocol used	Amount of uncut site, %	Ligation yield, %	Material	FA conc., %	RE	Conditions of chromatin treatment, °C
This study	In-nucleus ligation protocol	3.5	5	Drosophila cells	0.5	DpnII	65
Gavrilov, 2016	Dilution protocol	20	9	Mouse cells	2	MboI	37
van de Werken et al., 2012	*In situ* ligation protocol	20	ND^*^	Human cells	2	DpnII	37

* ND, Not determined

The percent of cut site in our conditions (96.6%) was much higher than achieved with the dilution (Gavrilov, 2016) and with *in situ* (van de Werken et al., 2012) protocols. This might be explained by the lower FA concentration used, the different source of experimental material, and different conditions of chromatin treatment in our study compared with the above works.

At the same time, the ligation yield in our study (~5%) was lower than with the dilution protocol (Gavrilov, 2016; Table 3; Figure 7G). This can be partly explained by using of different RE (DpnII instead of MboI) or is more likely be a consequence of different conditions of chromatin treatment [65°С in our study vs. 37°С in Gavrilov (2016)] since it seems that chromatin treatment at 37°С might give a higher ligation yield, as the Figures 5A–D imply.

In summary, the ratios between the amounts of non-cleaved and religated restriction site in our study were approximately the same as previously shown for mouse embryonic liver cells (Gavrilov, 2016), emphasizing the universality of our observations.

We additionally estimated the amount of a circular ligation product (284bp; Figure 7A). The amount was found to be more than 10 times lower than the amount of the regenerated ligation product (0.37%±0.10% vs. 5%; Figure 7G). Thus, ligation of adjacent restriction fragments is predominantly observed with our in-nucleus ligation protocol, whereas circularization and ligation of adjacent restriction fragments are equally possible for solubilized chromatin in the dilution protocol (Gavrilov, 2016). Our observations coincide with the observations of other authors (Arkadiy Golov and Maxim Imakaev, personal communications).

Thus, the absence of solubilization contributes to directional ligation of adjacent chromatin fragments.

**Figure 8:**
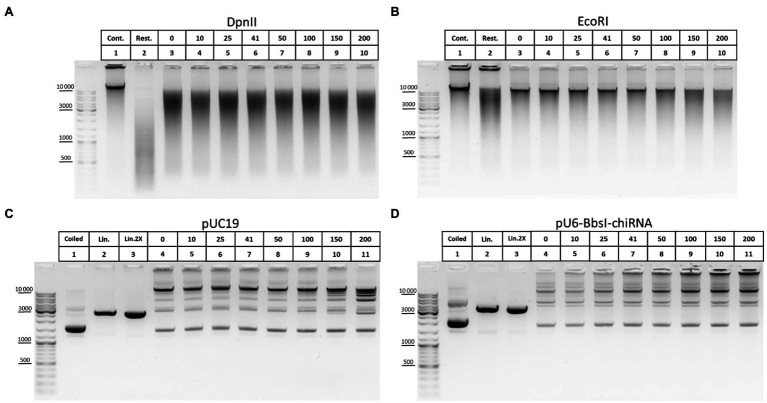
Inhibition of T4 DNA ligase activity with NaCl. **(A,B)** The ability of T4 DNA ligase to ligate DNA in the presence of NaCl using genomic DNA. Genomic DNA was isolated, dissolved, treated with RNase I, purified on AMPure XP beads (lane 1), and digested with DpnII in DpnII buffer pH 6.0 **(A)** or with EcoRI in EcoRI buffer **(B)** as in Figure 5E. After digestion, REs were heat inactivated and DNA was purified on AMPure XP beads and subjected to electrophoresis (lane 2) or ligated in solution as in Figure 6E in the presence of different concentrations of NaCl (indicated by the upper raw of figures presented above the picture). Then, DNA was desalted by purification on AMPure XP magnetic beads and subjected to electrophoresis. Lane 1, non-digested DNA; lane 2, digested DNA; and lanes 3–10, DNA ligated at different concentrations of NaCl (0–200mM). 41mM is a concentration on NaCl in ligation reaction from Comet et al. (2011). One of the three replicate experiments is shown. **(C,D)** The ability of T4 DNA ligase to ligate DNA in the presence of NaCl using plasmid DNA. Plasmid DNA of pUC19 **(C)** or of pU6-BbsI-chiRNA **(D)** was linearised using a unique EcoRI site in the backbone. Restriction digestion was performed using 10U of EcoRI in EcoRI buffer in a volume of 20μl. Then, RE was heat inactivated and DNA was precipitated and washed with ethanol as in Figures 2A–C, dissolved in 10mM Tris-HCl pH 8.0, ligated in solution as in Figure 5E, and subjected to electrophoresis. Lane 1, non-digested plasmid (400ng); lane 2, linearised plasmid (400ng); lane 3, a double amount of linearised plasmid was taken after precipitation with ethanol; and lanes 4–11, DNA ligated in the presence of different concentrations of NaCl (0–200mM). 41mM is a concentration on NaCl in ligation reaction proposed from Comet et al. (2011). One of the three replicate experiments is shown.

The ability of T4 DNA ligase to ligate DNA in the presence of NaCl was another important issue that we investigated concerning ligation. In some 3C protocols, a salt-free 1X T4 DNA ligase buffer is used to dilute the restriction reaction mixture after RE inactivation at 65°C (Comet et al., 2011; Rao et al., 2014; Vermeulen et al., 2020). However, 1X restriction buffer (RB) for DpnII contains 100mM NaCl, which has a potential inhibitory effect because T4 DNA ligase is salt-sensitive and is most active in a salt-free buffer (Raae et al., 1975; Hayashi et al., 1985). The effect of T4 DNA ligase inhibition with NaCl is manifested differently in ligation of DNA cut with different REs (Hayashi et al., 1985) and was not studied for DpnII. Unexpectedly, no significant inhibition of T4 DNA ligase was observed with up to 150–200mM NaCl (Figure 8A). However, the activity of T4 DNA ligase was inhibited at a much lower NaCl concentration in the above studies.

We assumed that the effect of salt inhibition of T4 DNA ligase activity would be more clearly detected if genomic DNA is digested with a 6-bp cutter since after ligation DNA assembles in solution into fragments of higher MWs, larger than 10–12kb (Figure 5E). Again, noticeable inhibition of ligation was observed sonly at NaCl concentrations of 150–200mM (Figure 8B). With plasmid DNA, such inhibition was not observed at all or was very slight at 150–200mM NaCl (Figures 8C,D).

We concluded that T4 DNA ligase can function efficiently in the presence of NaCl used at up to 100mM and that even 150–200mM NaCl is possible to use in the ligation reaction without significantly affecting the ligase activity.

The following questions are also discussed in the supplement: SDS sequestration with Triton X-100; washings of nuclei; optimization of Taq-man PCR conditions; reversion of cross-links and isolation of the 3C library (discussion of the role of EDTA, ionic strength, and dilution in maintaining DNA integrity in the 3C procedure and the role of the temperature and composition of the extraction buffer (EB) in maintaining DNA integrity in the 3C procedure); treatment of the 3C library with RNases; and purification of the 3C library on magnetic beads.

A detailed protocol of 3C library preparation consisting of the optimized steps is given in the Supplement.

## Discussion

In general, the 3C procedure requires optimization for each specific cell type. We provide a useful framework for optimization of the protocol and carry it out for *Drosophila* S2 cells.

A sequence of steps and features was combined into the 3C protocol to allow keeping the nuclei as intact as possible. The features include a lysis buffer that ensures hypotonic conditions (Tolhuis et al., 2002) and has the detergent concentrations as proposed by Splinter et al. (2012), van de Werken et al. (2012), a washing of the nuclei with a hypotonic lysis buffer after lysis in hypotonic conditions (Rao et al., 2014; the washing can also be done with 1X PBS, 1X RB, or even with water), a washing of the nuclei with 1X RB to remove SDS/Triton X-100 (Flyamer et al., 2017), and a washing of the nuclei with a 1X T4 DNA ligase buffer to remove the RE instead of heating the nuclei at 65°C to inactivate RE (Flyamer et al., 2017; Golov et al., 2020). The efficiency of these steps is evidenced, for example, from the absence of DNA release from nuclei upon their treatment at 37°C and a more distinct ligation pattern (Figures 5C–D).

We studied in detail the basic stages of the 3C protocol in this work, paying special attention to the preservation of DNA integrity throughout the procedure. The absence of DNA degradation at all stages makes the method as reliable as possible and results in reproducible profiles at an output.

The causes of DNA degradation are the most mysterious aspect of the method. We and others noticed that DNA degradation occurs early in the 3C protocol, often at the cell lysis step, and is commonly attributed to contaminating nucleases (Louwers et al., 2009; Naumova et al., 2012). Thus, initial stages of the protocol appear to be absolutely critical for maintaining DNA integrity throughout the procedure. According to our results, cell lysis and subsequent chromatin treatment with SDS/Triton X-100 are the most crucial steps in this regard. There are a lot of enzymes with DNase activity in eukaryotic cells, and some of them function in the nucleus, cytosol, and lysosomes (Yang, 2011; Kawane et al., 2014; Fujiwara et al., 2017). The isolated cytosol of eukaryotic cells exhibits divalent cation-dependent DNase activity (Lechardeur et al., 1999). We assumed that residual DNase activity may exert a detrimental effect on the integrity of a 3C DNA library during cell lysis and treatment of nuclei.

We found that treatment of cells with a hypotonic lysis buffer which provides for a more efficient release of nucleoplasmic proteins in comparison with an isotonic buffer (Méndez and Stillman, 2000; Golov et al., 2015) and a subsequent thorough washing of the nuclei ensure DNA integrity at subsequent stages. Nucleases may be released from fixed nuclei *via* diffusion through the disrupted nuclear envelope as fixed nuclei swell in hypotonic conditions in the 3C procedure (Gavrilov et al., 2013b).

It is conceivable that two groups of nucleases, cytoplasmic and nuclear, may be responsible for DNA degradation in the 3C procedure. Predominantly, cytoplasmic nucleases are likely to be inactivated and washed off during cell lysis, a washing of nuclei, and chromatin treatment at 65°C in isotonic conditions. The assumption is supported by the observations that a prolonged heating of nuclei at 65°С after isotonic lysis and a washing of nuclei were accompanied by DNA degradation, indicating nuclear damage and, probably, a nucleoplasm release. A transition to hypotonic conditions during reversion of cross-links in the 1X T4 DNA ligase buffer induces a release of nucleoplasmic nucleases, and high EDTA concentrations in EB are required for preventing DNA degradation. However, it is unclear how these nuclear nucleases avoid solubilization with SDS at the step of SDS/Triton X-100 treatment of nuclei at 65°C/37°C.

At the same time, some amount of genomic DNA was released into the supernatant fraction after hypotonic lysis, a washing of nuclei, and short-term heating at 65°C. The more severe the processing conditions, the greater amount of DNA was found in the supernatant. However, this release was not accompanied by DNA degradation in the supernatant and nuclear pellet fractions. The finding proved that hypotonic lysis is more efficient.

If nucleases are suspected to occur in the sample, as in the case of isotonic lysis or an improper washing of nuclei after lysis, dilution of the restriction reaction mixture with a large volume of a hypotonic 1X T4 DNA ligation buffer prior to DNA ligation may provide a means to preserve the integrity of the 3C library upon ligation and subsequent reversion of cross-links. This approach is unconsciously used in the dilution ligation protocol (Dekker et al., 2002; Tolhuis et al., 2002; Lieberman-Aiden et al., 2009; Comet et al., 2011; Stadhouders et al., 2013; Ulianov et al., 2016; Vermeulen et al., 2020). In other cases, the addition of EDTA at up to 30mM can help to preserve DNA integrity upon reversion of cross-links.

In experiments with *Drosophila* S2 cells, we found that the addition of EDTA above a certain threshold concentration (>22mM) prevents DNA degradation and maintains DNA integrity. The finding further confirms that DNA degradation is directed by nucleases depending on divalent cations and, primarily, Mg^2+^ as the most abundant divalent cation in the cell. Our results from experiments on preserving EDTA concentrations correlate well with the literature data on physiological concentrations of Mg^2+^ in cells and hemolymph of *Drosophila* larvae (Begg and Cruickshank, 1962; Larrivee, 1979; van der Meer and Jaffe, 1983; Stewart et al., 1994; Echalier et al., 2018), as well as in the extracellular and perivitelline fluids (~20mM; van der Meer and Jaffe, 1983).

Several other important findings made in our experiments are listed below.

Quality of the resulting 3C libraries may depend on how and with what RNase the library is treated. In our experiments, *E. coli* RNase I performed best. The enzyme did not degrade DNA at room temperature and 37°C even when used at high concentrations. In contrast, bovine RNase A, which is most frequently used in such experiments, often degraded the library at 37°C, but not at room temperature. An increase in bovine RNase A concentration led to complete DNA degradation at both room temperature and 37°C.

Quality of the resulting libraries may depend on the conditions of elution from magnetic beads at the step of additional purification of the library. When DNA was eluted with water at an elevated temperature of 55°C (heating facilitates elution), DNA degradation occurred, while DNA was stabilized in a Tris buffer or in the presence of NaCl.

An increase in SDS concentration during extraction of uncross-linked proteins from nuclei increases the amount of uncross-linked RNA released from the nuclei. The observation is important for studying chromatin-associated RNAs using the Hi-C method (Li et al., 2017; Sridhar et al., 2017; Bell et al., 2018; Yan et al., 2019; Bonetti et al., 2020; Gavrilov et al., 2020). In this method, SDS is routinely used to remove the cytoplasm and to extract the proteins and RNAs that have not been cross-linked to DNA before ligation of chromatin-associated RNAs to DNA. Our data suggest that, when RNA molecules are not cross-linked to DNA, but can participate in the formation of ligation products with DNA, harsh treatment with a high SDS concentration will decrease RNA-DNA cross-linking and cause a loss of signal.

We observed that non-fixed chromatin ligates much faster than fixed chromatin and that ligation in nuclei is strongly inhibited with 0.1% SDS even after its sequestration with 1% Triton X-100. Thus, it is necessary for efficient ligation to wash off SDS/Triton X-100 as suggested by Flyamer et al. (2017). We also noticed that DNA ligation in nuclei is slightly more efficient at 16°C in the presence of T4 DNA ligase at a concentration not exceeding 0.25U/μl.

We additionally showed that the circularization of a restriction fragment is a very rare event under the conditions of the *in situ*/in-nucleus protocol compared to direct ligation of adjacent DNA restriction fragments. We measured the ligation frequencies for only one site; however, it was previously shown that values measured for several sites do not fundamentally differ (Gavrilov and Razin, 2008).

No ligation yield was observed after digestion and ligation of non-fixed chromatin (compare absolute values in Figures 7A,F,G). At the same time, we showed that non-fixed chromatin is ligated much faster than fixed chromatin (compare Figure 5F and Figures 6B,C). This discrepancy can be explained by the fact that mass ligation of DNA fragments still proceeds in non-fixed nuclei, although specific interactions of restriction fragments are lost in non-fixed chromatin (Dekker et al., 2002; Gavrilov and Razin, 2008; Belaghzal et al., 2017). In addition, non-fixed chromatin is less frequently cleaved (Figure 7A), thus producing DNA fragments of higher MWs. The fragments are ligated in a pattern that mimics efficient ligation (compare the patterns obtained with 6-bp and 4-bp cutters in Figure 5E). As a result, the library from non-fixed cells looks as having been efficiently ligated but contains very few specific ligation products (Dekker et al., 2002; Gavrilov and Razin, 2008; Belaghzal et al., 2017). Thus, non-fixed chromatin is cut worse and ligated faster, but this ligation turns out to be meaningless, while fixed chromatin is cut more efficiently and ligated slower, but this ligation makes sense.

The conditions that we selected for S2 cells are also suitable for *Drosophila* larvae [(Shidlovskii et al., 2021); Bylino et al., manuscript in preparation]. We suppose that the same conditions might be suitable for working with more complex models than *Drosophila* tissues, including mammalian and human cells and mammalian tissues. However, additional steps of tissue processing with collagenase, elastase, or trypsin should be introduced into the procedure to obtain a cell suspension in this case. After isolation, cells can be treated using the steps shown and discussed here. We hope that the sequence of steps proposed here may be useful for single-cell methods since several steps were taken from the work by Flyamer et al. (2017), which focused on single cells. We summarized our results in Table 4.

**Table 4:** Conditions found to be optimal for stages of the 3С protocol.

#	Stage of the protocol	Conditions studied	Optimal
1	Inactivation of FA with glycine	Inactivation with 0.125M glycine Inactivation with equimolar or slightly excessive glycine	Inactivation with equimolar or slightly excessive glycine
	Storage of fixed nuclei	After using 0.125M glycine After using equimolar or slightly excessive glycine	Move immediately to the lysis stage without storage of nuclei
2	Lysis	Contributions of various components of the lysis buffer	Hypotonic conditions (10mM NaCl) with high amounts of non-ionic detergents (1% Triton X-100+0.5% NP-40)
3	Chromatin heating in the presence of SDS (nucleoplasm release)	(1) 65°C for 10min, (2) 65°C for 5min, (3) 37°C for 10min, (4) 37°C for 1h at different SDS concentrations (0.1, 0.3, 0.5%)	65°C for 5min or 37°C for 10min with 0.1–0.3% SDS
4	Sequestration of SDS with Triton X-100	The following chromatin treatment regimens were studied (SDS/Triton X-100 concentrations were 0.1 and 1.8%, respectively): (1) 65°C for 10min with SDS in 1X RB+37°C for 1h with Triton X-100 in 1X RB, (2) 65°C for10 min with SDS in 1X RB+addition of Triton X-100 without any incubation, (3) 65°C for 10min with SDS in water +37°C for 15min with Triton X-100 in water, (4) 65°C for10 min with SDS in 1X RB+37°C for 15min with Triton X-100 in 1X RB	65°С, 10min, SDS in water +37°C, 15min, Triton X-100 in water
5	Washing of nuclei after cell lysis	(1) With hypotonic lysis buffer containing 1% Triton X-100 and 0.5% NP-40, (2) With 1X RB, (3) With 1X PBS, (4) With water	With hypotonic lysis buffer (1X RB, 1X PBS and water can also be used)
	Washing of nuclei after treatment with SDS/Triton X-100	(1) Without washing (addition of 10X RB into water containing SDS/Triton X-100), (2) Without washing (centrifugation, removing the supernatant, and resuspension of nuclei in 1X RB), (3) Washing three times with 1X RB, (4) Washing once with 1X PBS and resuspension in 1X RB	Washing three times with 1X RB
	Washing of nuclei after restriction digestion with or without prior RE inactivation at 65°C for 20min	(1) Without washing [dilution of restriction mixture with 1X T4 DNA ligase buffer (1X T4 LB)], (2) Washing once with 1X LB, (3) Without washing (centrifugation, removing the supernatant, and resuspension of nuclei in 1X T4 LB), (4) Washing three times with 1X T4 LB	Without RE inactivation at 65°C for 20min+washing three times with 1X T4 LB
6	Chromatin digestion in nuclei	The following regimens after different heat treatment were studied (treatment with SDS/Triton X-100 was done in water; SDS and Triton X-100 concentration was 0.3 and 1.8%, respectively; cell fixation was carried out at 1% FA): (1) 65°C for10 min with SDS+37°C for 15min with Triton X-100, (2) 65°C for 5min with SDS+37°C for 15min with Triton X-100, (3) 37°C for 10min with SDS+37°C for 15min with Triton X-100, (4) 37°C for 1h with SDS+37°C for 1h with Triton X-100. Was studied after cell fixation with different FA concentrations (chromatin treatment was done at 65°C for 5min with 0.3% SDS in water+37°C for 15min with 1.8% Triton X-100 in water): 0.5, 0.25, 0.1, 0%	37°C for 1h with SDS+37°C for 1h with Triton X-100 or 65°C for 5min with SDS+37°C for 15min with Triton X-100 0.5% FA
7	DNA ligation in nuclei	Duration of ligation: 1h, overnight Presence of 0.1% SDS and 1% Triton X-100 Effect of 1% Triton X-100 Effect of T4 DNA ligase concentration: 1, 0.25, 0.025, 0.0025U/μl Effect of the reaction temperature: 16°C, RT (22°C) Effect of NaCl concentration: 10–200mM	Overnight Exclude 0.1% SDS sequestered with 1% Triton X-100 from ligation reaction Does not affect the ligation process; at 0.1% prevents nuclei from sticking to tube walls upon shaking Range 0.25–0.025U/μl. 16°C Ligation is most effective at up to 100mM NaCl and can be carried out without significant loss of T4 DNA ligase activity at up to 150–200mM NaCl
8	Reversion of cross-links and isolation of 3C library	Effect of EDTA concentration in EB on DNA integrity Effect of diluting restriction mixture with 1X T4 LB on DNA integrity Influence of temperature on efficiency of cross-link reversion Effect of ionic strength and EB composition on cross-link reversion and the success of DNA extraction with Ph/Chl	30mM EDTA is required to maintain DNA integrity after cell lysis in isotonic conditions, but not after cell lysis in hypotonic conditions Dilution helps to maintain DNA integrity after lysis in isotonic conditions, but is not required after lysis in hypotonic conditions Reversion of cross-links is efficient at 56°C for 13.5h or 50°C for 19h Hypertonic conditions are not recommended. In isotonic conditions, adding 1% SDS or 30mM EDTA is optional. In hypotonic conditions, adding 1% SDS or 30mM EDTA is required
9	Treatment of 3C library with RNases	Bovine RNase A, Recombinant RNase A, RNase If, RNase T1, RNase I	RNase I
10	Purification of 3C library on magnetic beads (SPRI)	Elution from beads with: Water at 55°C and at RT 10 or 50mM Tris-HCl pH 8.0, 10 or 25mM NaCl	Elution with water at RT Elution with 10–50mM Tris-HCl pH 8.0 or 10–25mM NaCl at RT or at 55°C
11	DNA storage	On magnetic beads: overnight at RT and at −20°C under 75% ethanol On ice: over a weekend, for a week, for 2weeks	Any condition is suitable

## Conclusion

We characterized the critical points of the 3C procedure and offer options to bypass these bottlenecks. Improvements introduced to the procedure make it possible to carry out the 3C method with the maximum yield, to preserve DNA integrity at all stages, and to increase the stability and reproducibility of the method.

## Data Availability Statement

The datasets presented in this study can be found in online repositories. The names of the repository/repositories and accession number(s) can be found at European Nucleotide Archive, accession no: ERR6474674.

## Author Contributions

YS and OB: conceptualization and methodology. OB: formal analysis and investigation. OB, AP, and YS: writing. OB and AI: visualization. YS: supervision, project administration, and funding acquisition. All authors contributed to manuscript revision, read, and approved the submitted version.

## Funding

This study was supported by the Russian Science Foundation (project 20–14-00201).

## Conflict of Interest

The authors declare that the research was conducted in the absence of any commercial or financial relationships that could be construed as a potential conflict of interest.

## Publisher’s Note

All claims expressed in this article are solely those of the authors and do not necessarily represent those of their affiliated organizations, or those of the publisher, the editors and the reviewers. Any product that may be evaluated in this article, or claim that may be made by its manufacturer, is not guaranteed or endorsed by the publisher.

## References

[ref1] Begg M. Cruickshank W. J. (1962). XVI—A partial analysis of drosophila larval Hæmolymph. Proc. Royal Soc. Edin. Sec. B. Biol. Sci. 68, 215–236. doi: 10.1017/S0080455X00001053

[ref2] Belaghzal H. Dekker J. Gibcus J. H. (2017). Hi-C 2.0: An optimized hi-C procedure for high-resolution genome-wide mapping of chromosome conformation. Methods 123, 56–65. doi: 10.1016/j.ymeth.2017.04.004, PMID: 28435001 PMC5522765

[ref3] Bell J. C. Jukam D. Teran N. A. Risca V. I. Smith O. K. Johnson W. L.. (2018). Chromatin-associated RNA sequencing (ChAR-seq) maps genome-wide RNA-to-DNA contacts. Elife 7:e27024. doi: 10.7554/eLife.27024, PMID: 29648534 PMC5962340

[ref4] Belton J.-M. McCord R. P. Gibcus J. Naumova N. Zhan Y. Dekker J. (2012). Hi-C: A comprehensive technique to capture the conformation of genomes. Methods 58, 268–276. doi: 10.1016/j.ymeth.2012.05.001, PMID: 22652625 PMC3874846

[ref5] Bonetti A. Agostini F. Suzuki A. M. Hashimoto K. Pascarella G. Gimenez J.. (2020). RADICL-seq identifies general and cell type-specific principles of genome-wide RNA-chromatin interactions. Nat. Commun. 11:1018. doi: 10.1038/s41467-020-14337-6 32094342 PMC7039879

[ref6] Campos P. F. Gilbert T. M. P. (2012). DNA extraction from formalin-fixed material. Methods Mol. Biol. 840, 81–85. doi: 10.1007/978-1-61779-516-9_11 22237525

[ref7] Caudron-Herger M. Rippe K. (2012). Nuclear architecture by RNA. Curr. Opin. Genet. Dev. 22, 179–187. doi: 10.1016/j.gde.2011.12.005, PMID: 22281031

[ref8] Chathoth K. T. Zabet N. R. (2019). Chromatin architecture reorganization during neuronal cell differentiation in drosophila genome. Genome Res. 29, 613–625. doi: 10.1101/gr.246710.118, PMID: 30709849 PMC6442379

[ref9] Comet I. Schuettengruber B. Sexton T. Cavalli G. (2011). A chromatin insulator driving three-dimensional Polycomb response element (PRE) contacts and Polycomb association with the chromatin fiber. PNAS 108, 2294–2299. doi: 10.1073/pnas.1002059108 21262819 PMC3038747

[ref10] Cubeñas-Potts C. Rowley M. J. Lyu X. Li G. Lei E. P. Corces V. G. (2017). Different enhancer classes in drosophila bind distinct architectural proteins and mediate unique chromatin interactions and 3D architecture. Nucleic Acids Res. 45, 1714–1730. doi: 10.1093/nar/gkw1114, PMID: 27899590 PMC5389536

[ref11] Cullen K. E. Kladde M. P. Seyfred M. A. (1993). Interaction between transcription regulatory regions of prolactin chromatin. Science 261, 203–206. doi: 10.1126/science.8327891, PMID: 8327891

[ref12] Dekker J. (2006). The three “C” s of chromosome conformation capture: controls, controls, controls. Nat. Methods 3, 17–21. doi: 10.1038/nmeth823, PMID: 16369547

[ref13] Dekker J. (2007). GC- and AT-rich chromatin domains differ in conformation and histone modification status and are differentially modulated by Rpd3p. Genome Biol. 8:R116. doi: 10.1186/gb-2007-8-6-r116, PMID: 17577398 PMC2394764

[ref14] Dekker J. Belmont A. S. Guttman M. Leshyk V. O. Lis J. T. Lomvardas S.. (2017). The 4D nucleome project. Nature 549, 219–226. doi: 10.1038/nature23884, PMID: 28905911 PMC5617335

[ref15] Dekker J. Rippe K. Dekker M. Kleckner N. (2002). Capturing chromosome conformation. Science 295, 1306–1311. doi: 10.1126/science.1067799, PMID: 11847345

[ref16] Ding D.-Q. Okamasa K. Katou Y. Oya E. Nakayama J.-I. Chikashige Y.. (2019). Chromosome-associated RNA-protein complexes promote pairing of homologous chromosomes during meiosis in Schizosaccharomyces pombe. Nat. Commun. 10:5598. doi: 10.1038/s41467-019-13609-0, PMID: 31811152 PMC6898681

[ref17] Downes D. J. Beagrie R. A. Gosden M. E. Telenius J. Carpenter S. J. Nussbaum L.. (2021). High-resolution targeted 3C interrogation of cis -regulatory element organization at genome-wide scale. Nat. Commun. 12:531. doi: 10.1038/s41467-020-20809-6, PMID: 33483495 PMC7822813

[ref18] Eagen K. P. Aiden E. L. Kornberg R. D. (2017). Polycomb-mediated chromatin loops revealed by a subkilobase-resolution chromatin interaction map. PNAS 114, 8764–8769. doi: 10.1073/pnas.1701291114 28765367 PMC5565414

[ref19] Eagen K. P. Hartl T. A. Kornberg R. D. (2015). Stable chromosome condensation revealed by chromosome conformation capture. Cell 163, 934–946. doi: 10.1016/j.cell.2015.10.026, PMID: 26544940 PMC4639323

[ref20] Echalier G. Perrimon N. Mohr S. E. (2018). Drosophila Cells in Culture. Netherlands: Elsevier.

[ref21] El-Sharnouby S. Fischer B. Magbanua J. P. Umans B. Flower R. Choo S. W.. (2017). Regions of very low H3K27me3 partition the drosophila genome into topological domains. PLoS One 12:e0172725. doi: 10.1371/journal.pone.0172725, PMID: 28282436 PMC5345799

[ref22] Falk M. Feodorova Y. Naumova N. Imakaev M. Lajoie B. R. Leonhardt H.. (2019). Heterochromatin drives compartmentalization of inverted and conventional nuclei. Nature 570, 395–399. doi: 10.1038/s41586-019-1275-3, PMID: 31168090 PMC7206897

[ref23] Flyamer I. M. Gassler J. Imakaev M. Brandão H. B. Ulianov S. V. Abdennur N.. (2017). Single-nucleus hi-C reveals unique chromatin reorganization at oocyte-to-zygote transition. Nature 544, 110–114. doi: 10.1038/nature21711, PMID: 28355183 PMC5639698

[ref24] Fujiwara Y. Wada K. Kabuta T. (2017). Lysosomal degradation of intracellular nucleic acids—multiple autophagic pathways. J. Biochem. 161, 145–154. doi: 10.1093/jb/mvw085, PMID: 28039390

[ref25] Gabdank I. Ramakrishnan S. Villeneuve A. M. Fire A. Z. (2016). A streamlined tethered chromosome conformation capture protocol. BMC Genomics 17:274. doi: 10.1186/s12864-016-2596-3 27036078 PMC4818521

[ref26] Gavrilov A. A. (2016). Doctor of Sc. Thesis. Spatial organization of the eukaryote genome in the context of transcription regulation. Available at: http://www.genebiology.ru/dissovet/o-dissertatcionnom-sovete/. (Accessed August 27, 2021).

[ref27] Gavrilov A. A. Golov A. K. Razin S. V. (2013a). Actual ligation frequencies in the chromosome conformation capture procedure. PLoS One 8:e60403. doi: 10.1371/journal.pone.0060403, PMID: 23555968 PMC3608588

[ref28] Gavrilov A. A. Gushchanskaya E. S. Strelkova O. Zhironkina O. Kireev I. I. Iarovaia O. V.. (2013b). Disclosure of a structural milieu for the proximity ligation reveals the elusive nature of an active chromatin hub. Nucleic Acids Res. 41, 3563–3575. doi: 10.1093/nar/gkt067 23396278 PMC3616722

[ref29] Gavrilov A. A. Razin S. V. (2008). Spatial configuration of the chicken α-globin gene domain: immature and active chromatin hubs. Nucleic Acids Res. 36, 4629–4640. doi: 10.1093/nar/gkn429, PMID: 18621783 PMC2504291

[ref30] Gavrilov A. Razin S. V. (2009). Formaldehyde fixation of cells does not greatly reduce the ability to amplify cellular DNA. Anal. Biochem. 390, 94–96. doi: 10.1016/j.ab.2009.04.018, PMID: 19376079

[ref31] Gavrilov A. Razin S. V. Cavalli G. (2015). In vivo formaldehyde cross-linking: it is time for black box analysis. Brief. Funct. Genomics 14, 163–165. doi: 10.1093/bfgp/elu037, PMID: 25241225 PMC6090872

[ref32] Gavrilov A. A. Zharikova A. A. Galitsyna A. A. Luzhin A. V. Rubanova N. M. Golov A. K.. (2020). Studying RNA-DNA interactome by red-C identifies noncoding RNAs associated with various chromatin types and reveals transcription dynamics. Nucleic Acids Res. 48, 6699–6714. doi: 10.1093/nar/gkaa457, PMID: 32479626 PMC7337940

[ref33] Golov A. K. Gavrilov A. A. Razin S. V. (2015). The role of crowding forces in juxtaposing β-globin gene domain remote regulatory elements in mouse erythroid cells. PLoS One 10:e0139855. doi: 10.1371/journal.pone.0139855, PMID: 26436546 PMC4593578

[ref34] Golov A. K. Ulianov S. V. Luzhin A. V. Kalabusheva E. P. Kantidze O. L. Flyamer I. M.. (2020). C-TALE, a new cost-effective method for targeted enrichment of hi-C/3C-seq libraries. Methods 170, 48–60. doi: 10.1016/j.ymeth.2019.06.022, PMID: 31252062

[ref35] Gridina M. Mozheiko E. Valeev E. Nazarenko L. P. Lopatkina M. E. Markova Z. G.. (2021). A cookbook for DNase hi-C. Epigenetics Chromatin 14:15. doi: 10.1186/s13072-021-00389-5, PMID: 33743768 PMC7981840

[ref36] Hagège H. Klous P. Braem C. Splinter E. Dekker J. Cathala G.. (2007). Quantitative analysis of chromosome conformation capture assays (3C-qPCR). Nat. Protoc. 2, 1722–1733. doi: 10.1038/nprot.2007.243, PMID: 17641637

[ref37] Hall L. L. Lawrence J. B. (2016). RNA as a fundamental component of interphase chromosomes: could repeats prove key? Curr. Opin. Genet. Dev. 37, 137–147. doi: 10.1016/j.gde.2016.04.005, PMID: 27218204 PMC4918761

[ref38] Hayashi K. Nakazawa M. Ishizaki Y. Obayashi A. (1985). Influence of monovalent cations on the activity of T4 DNA ligase in the presence of polyethylene glycol. Nucleic Acids Res. 13, 3261–3271. doi: 10.1093/nar/13.9.3261, PMID: 2987879 PMC341233

[ref39] Hou C. Li L. Qin Z. S. Corces V. G. (2012). Gene density, transcription, and insulators contribute to the partition of the drosophila genome into physical domains. Mol. Cell 48, 471–484. doi: 10.1016/j.molcel.2012.08.031, PMID: 23041285 PMC3496039

[ref40] Hug C. B. Grimaldi A. G. Kruse K. Vaquerizas J. M. (2017). Chromatin architecture emerges during zygotic genome activation independent of transcription. Cell 169, 216–228. doi: 10.1016/j.cell.2017.03.024 28388407

[ref41] Kalhor R. Tjong H. Jayathilaka N. Alber F. Chen L. (2012). Genome architectures revealed by tethered chromosome conformation capture and population-based modeling. Nat. Biotechnol. 30, 90–98. doi: 10.1038/nbt.2057, PMID: 22198700 PMC3782096

[ref42] Kawane K. Motani K. Nagata S. (2014). DNA degradation and its defects. Cold Spring Harb. Perspect. Biol. 6:a016394. doi: 10.1101/cshperspect.a016394, PMID: 24890510 PMC4031964

[ref43] Kim T. H. Dekker J. (2018a). ChIP. Cold Spring Harb. Protoc. 4, 314–316. doi: 10.1101/pdb.prot082610, PMID: 29610359

[ref44] Kim T. H. Dekker J. (2018b). Formaldehyde cross-linking. Cold Spring Harb. Protoc. 4, 306–310. doi: 10.1101/pdb.prot082594 29610357

[ref45] Larrivee D. C. (1979). *A Biochemical Analysis of the Drosophila Rhabdomere and its Extracellular Environment. Ph.D. Thesis*. Doctoral Dissertation. Indiana: Purdue University

[ref46] Lechardeur D. Sohn K.-J. Haardt M. Joshi P. B. Monck M. Graham R. W.. (1999). Metabolic instability of plasmid DNA in the cytosol: a potential barrier to gene transfer. Gene Ther. 6, 482–497. doi: 10.1038/sj.gt.3300867, PMID: 10476208

[ref47] Li L. Lyu X. Hou C. Takenaka N. Nguyen H. Q. Ong C.-T.. (2015). Widespread rearrangement of 3D chromatin organization underlies Polycomb-mediated stress-induced silencing. Mol. Cell 58, 216–231. doi: 10.1016/j.molcel.2015.02.023, PMID: 25818644 PMC4402144

[ref48] Li X. Zhou B. Chen L. Gou L.-T. Li H. Fu X.-D. (2017). GRID-seq reveals the global RNA-chromatin interactome. Nat. Biotechnol. 35, 940–950. doi: 10.1038/nbt.3968, PMID: 28922346 PMC5953555

[ref49] Lieberman-Aiden E. van Berkum N. L. Williams L. Imakaev M. Ragoczy T. Telling A.. (2009). Comprehensive mapping of long-range interactions reveals folding principles of the human genome. Science 326, 289–293. doi: 10.1126/science.1181369, PMID: 19815776 PMC2858594

[ref50] Lo Sardo F. (2021). “The chromosome conformation capture (3C) in Drosophila melanogaster,” in Capturing Chromosome Conformation: Methods and Protocols Methods in Molecular Biology. eds. Bodega B. Lanzuolo C. (New York, NY: Springer US), 9–17. 10.1007/978-1-0716-0664-3_2 32820396

[ref51] Louwers M. Splinter E. van Driel R. de Laat W. Stam M. (2009). Studying physical chromatin interactions in plants using chromosome conformation capture (3C). Nat. Protoc. 4, 1216–1229. doi: 10.1038/nprot.2009.113, PMID: 19644461

[ref52] Méndez J. Stillman B. (2000). Chromatin Association of Human Origin Recognition Complex, Cdc6, and Minichromosome maintenance proteins during the cell cycle: assembly of Prereplication complexes in late mitosis. Mol. Cell. Biol. 20, 8602–8612. doi: 10.1128/MCB.20.22.8602-8612.2000, PMID: 11046155 PMC102165

[ref53] Michieletto D. Gilbert N. (2019). Role of nuclear RNA in regulating chromatin structure and transcription. Curr. Opin. Cell Biol. 58, 120–125. doi: 10.1016/j.ceb.2019.03.007, PMID: 31009871 PMC6694202

[ref54] Miele A. Gheldof N. Tabuchi T. M. Dostie J. Dekker J. (2006). Mapping chromatin interactions by chromosome conformation capture. Curr. Protoc. Mol. Biol. 74, 21.11.1–21.11.20. doi: 10.1002/0471142727.mb2111s74 18265379

[ref55] Nagano T. Lubling Y. Stevens T. J. Schoenfelder S. Yaffe E. Dean W.. (2013). Single-cell hi-C reveals cell-to-cell variability in chromosome structure. Nature 502, 59–64. doi: 10.1038/nature12593, PMID: 24067610 PMC3869051

[ref56] Nagano T. Lubling Y. Várnai C. Dudley C. Leung W. Baran Y.. (2017). Cell-cycle dynamics of chromosomal organization at single-cell resolution. Nature 547, 61–67. doi: 10.1038/nature23001, PMID: 28682332 PMC5567812

[ref57] Nagano T. Lubling Y. Yaffe E. Wingett S. W. Dean W. Tanay A.. (2015a). Single-cell hi-C for genome-wide detection of chromatin interactions that occur simultaneously in a single cell. Nat. Protoc. 10, 1986–2003. doi: 10.1038/nprot.2015.127 26540590

[ref58] Nagano T. Várnai C. Schoenfelder S. Javierre B.-M. Wingett S. W. Fraser P. (2015b). Comparison of hi-C results using in-solution versus in-nucleus ligation. Genome Biol. 16:175. doi: 10.1186/s13059-015-0753-7, PMID: 26306623 PMC4580221

[ref59] Naumova N. Imakaev M. Fudenberg G. Zhan Y. Lajoie B. R. Mirny L. A.. (2013). Organization of the mitotic chromosome. Science 342, 948–953. doi: 10.1126/science.1236083, PMID: 24200812 PMC4040465

[ref60] Naumova N. Smith E. M. Zhan Y. Dekker J. (2012). Analysis of long-range chromatin interactions using chromosome conformation capture. Methods 58, 192–203. doi: 10.1016/j.ymeth.2012.07.022, PMID: 22903059 PMC3874837

[ref61] Oksuz B. A. Yang L. Abraham S. Venev S. V. Krietenstein N. Parsi K. M.. (2020). Systematic evaluation of chromosome conformation capture assays. BioRxiv 2020:12.26.424448. doi: 10.1101/2020.12.26.424448 PMC8446342 34480151

[ref62] Orlando V. Strutt H. Paro R. (1997). Analysis of chromatin structure by in vivo formaldehyde cross-linking. Methods 11, 205–214. doi: 10.1006/meth.1996.0407, PMID: 8993033

[ref63] Raae A. J. Kleppe R. K. Kleppe K. (1975). Kinetics and effect of salts and polyamines on T4 polynucleotide ligase. Eur. J. Biochem. 60, 437–443. doi: 10.1111/j.1432-1033.1975.tb21021.x, PMID: 173544

[ref64] Rao S. S. P. Huntley M. H. Durand N. C. Stamenova E. K. Bochkov I. D. Robinson J. T.. (2014). A three-dimensional map of the human genome at kilobase resolution reveals principles of chromatin looping. Cell 159, 1665–1680. doi: 10.1016/j.cell.2014.11.021, PMID: 25497547 PMC5635824

[ref65] Razin S. V. Ulianov S. V. Gavrilov A. A. (2019). 3D genomics. Mol. Biol. (Mosk) 53, 911–923. doi: 10.1134/S0026898419060156 31876272

[ref66] Rowley M. J. Nichols M. H. Lyu X. Ando-Kuri M. Rivera I. S. M. Hermetz K.. (2017). Evolutionarily conserved principles predict 3D chromatin organization. Mol. Cell 67, 837–852. doi: 10.1016/j.molcel.2017.07.022 28826674 PMC5591081

[ref67] Schalbetter S. A. Fudenberg G. Baxter J. Pollard K. S. Neale M. J. (2019). Principles of meiotic chromosome assembly revealed in *S. cerevisiae*. Nat. Commun. 10:4795. doi: 10.1038/s41467-019-12629-0, PMID: 31641121 PMC6805904

[ref68] Sexton T. Yaffe E. Kenigsberg E. Bantignies F. Leblanc B. Hoichman M.. (2012). Three-dimensional folding and functional organization principles of the drosophila genome. Cell 148, 458–472. doi: 10.1016/j.cell.2012.01.010, PMID: 22265598

[ref69] Shidlovskii Y. V. Bylino O. V. Shaposhnikov A. V. Kachaev Z. M. Lebedeva L. A. Kolesnik V. V.. (2021). Subunits of the PBAP chromatin Remodeler are capable of mediating enhancer-driven transcription in drosophila. Int. J. Mol. Sci. 22:2856. doi: 10.3390/ijms22062856, PMID: 33799739 PMC7999800

[ref70] Splinter E. de Wit E. van de Werken H. J. G. Klous P. de Laat W. (2012). Determining long-range chromatin interactions for selected genomic sites using 4C-seq technology: from fixation to computation. Methods 58, 221–230. doi: 10.1016/j.ymeth.2012.04.009, PMID: 22609568

[ref71] Splinter E. Grosveld F. de Laat W. (2004). 3C technology: analyzing the spatial organization of genomic loci in vivo. Methods Enzymol. 375, 493–507. doi: 10.1016/s0076-6879(03)75030-7, PMID: 14870685

[ref72] Sridhar B. Rivas-Astroza M. Nguyen T. C. Chen W. Yan Z. Cao X.. (2017). Systematic mapping of RNA-chromatin interactions In vivo. Curr. Biol. 27, 602–609. doi: 10.1016/j.cub.2017.01.011, PMID: 28132817 PMC5319903

[ref73] Stadhouders R. Kolovos P. Brouwer R. Zuin J. van den Heuvel A. Kockx C.. (2013). Multiplexed chromosome conformation capture sequencing for rapid genome-scale high-resolution detection of long-range chromatin interactions. Nat. Protoc. 8, 509–524. doi: 10.1038/nprot.2013.018, PMID: 23411633

[ref74] Stadler M. R. Haines J. E. Eisen M. B. (2017). Convergence of topological domain boundaries, insulators, and polytene interbands revealed by high-resolution mapping of chromatin contacts in the early Drosophila melanogaster embryo. elife 6:e29550. doi: 10.7554/eLife.29550 29148971 PMC5739541

[ref75] Stewart B. A. Atwood H. L. Renger J. J. Wang J. Wu C.-F. (1994). Improved stability of drosophila larval neuromuscular preparations in haemolymph-like physiological solutions. J. Comp. Physiol. A 175, 179–191. doi: 10.1007/BF00215114, PMID: 8071894

[ref76] Thakur J. Henikoff S. (2020). Architectural RNA in chromatin organization. Biochem. Soc. Trans. 48, 1967–1978. doi: 10.1042/BST20191226, PMID: 32897323 PMC7609026

[ref77] Tolhuis B. Palstra R.-J. Splinter E. Grosveld F. de Laat W. (2002). Looping and interaction between hypersensitive sites in the active β-globin locus. Mol. Cell 10, 1453–1465. doi: 10.1016/S1097-2765(02)00781-5, PMID: 12504019

[ref78] Ulianov S. V. Doronin S. A. Khrameeva E. E. Kos P. I. Luzhin A. V. Starikov S. S.. (2019). Nuclear lamina integrity is required for proper spatial organization of chromatin in drosophila. Nat. Commun. 10, 1–11. doi: 10.1038/s41467-019-09185-y 30862957 PMC6414625

[ref79] Ulianov S. V. Khrameeva E. E. Gavrilov A. A. Flyamer I. M. Kos P. Mikhaleva E. A.. (2016). Active chromatin and transcription play a key role in chromosome partitioning into topologically associating domains. Genome Res. 26, 70–84. doi: 10.1101/gr.196006.115, PMID: 26518482 PMC4691752

[ref80] Ulianov S. V. Zakharova V. V. Galitsyna A. A. Kos P. I. Polovnikov K. E. Flyamer I. M.. (2021). Order and stochasticity in the folding of individual drosophila genomes. Nat. Commun. 12:41. doi: 10.1038/s41467-020-20292-z, PMID: 33397980 PMC7782554

[ref81] van Berkum N. L. Dekker J. (2009). “determining spatial chromatin organization of large genomic regions using 5C technolosgy,” in Chromatin Immunoprecipitation Assays: Methods and Protocols Methods in Molecular Biology. ed. Collas P. (Totowa, NJ: Humana Press), 189–213. 10.1007/978-1-60327-414-2_13 PMC3880132 19588094

[ref82] van de Werken H. J. G. de Vree P. J. P. Splinter E. Holwerda S. J. B. Klous P. de Wit E.. (2012). “4C technology: protocols and data analysis,” in Methods in Enzymology. eds. Wu C. David Allis C. (Netherlands: Elsevier), 89–112. 10.1016/B978-0-12-391938-0.00004-5 22929766

[ref83] van der Meer J. M. Jaffe L. F. (1983). Elemental composition of the perivitelline fluid in early drosophila embryos. Dev. Biol. 95, 249–252. doi: 10.1016/0012-1606(83)90025-8, PMID: 6402395

[ref84] Vermeulen C. Allahyar A. Bouwman B. A. M. Krijger P. H. L. Verstegen M. J. A. M. Geeven G.. (2020). Multi-contact 4C: long-molecule sequencing of complex proximity ligation products to uncover local cooperative and competitive chromatin topologies. Nat. Protoc. 15, 364–397. doi: 10.1038/s41596-019-0242-7, PMID: 31932773

[ref85] Yan Z. Huang N. Wu W. Chen W. Jiang Y. Chen J.. (2019). Genome-wide colocalization of RNA-DNA interactions and fusion RNA pairs. Proc. Natl. Acad. Sci. U. S. A. 116, 3328–3337. doi: 10.1073/pnas.1819788116, PMID: 30718424 PMC6386723

[ref86] Yang W. (2011). Nucleases: diversity of structure, function and mechanism. Q. Rev. Biophys. 44, 1–93. doi: 10.1017/S0033583510000181, PMID: 20854710 PMC6320257

[ref87] Zhang Y. Li G. (2020). Advances in technologies for 3D genomics research. Sci. China Life Sci. 63, 811–824. doi: 10.1007/s11427-019-1704-2, PMID: 32394244

