# Supplementary material for: Investigation of the Basic Steps in the Chromosome Conformation Capture Procedure
Source: Front Genet. 2021 Sep 20;12:733937. doi: 10.3389/fgene.2021.733937 (PMC8488379; doi:10.3389/fgene.2021.733937)
Supplement: Supplementary file 1 [file Data_Sheet_1.pdf]

Supplementary Materials for the paper

Investigation of the basic steps in the Chromosome Conformation Capture Procedure

by Oleg V. Bylino, Airat N. Ibragimov, Anna E. Pravednikova, Yulii V. Shidlovskii

1 Analysis of other steps of the 3C procedure

1.1 Formaldehyde inactivation

Table S1. The amount of glycine required for inactivation of different amounts of FA

FA, %	Molarity, mM	Molarity by the number of FA reactive groups, mM	The amount of glycine required for equimolar inactivation of FA, mM	The amount of glycine required for inactivation of FA with slight excess to FA, mM
0.1	33.3	66.6	66.6	80
0.25	83.25	166.5	166.5	200
0.5	166.5	333	333	400
1	333	666	666	800
2	666	1332	1332	1600
3	999	1998	1998	2400
4	1332	2664	2664	3200

1.2 SDS Sequestration with Triton X-100

In the works that laid the foundation for the 3C method, both classical and more modern, it was proposed to use different options for the duration of chromatin incubation with SDS and with SDS/Triton X-100. Decker's original protocol proposed to treat nuclei briefly (for 10 min) with 0.1% SDS and then to sequester SDS with Triton X-100 at a 1:10 ratio (0.1% SDS, 1% Triton) without a prolonged incubation with the two agents prior to adding a restriction endonuclease (RE) (Dekker et al., 2002). In a later work by (Miele et al., 2006a), the step of incubation at 37 °C for 10 min was changed to incubation at 65 ° C for 10 min. This short incubation at 65 °C was similarly used in tethering ligation protocol of (Kalhor et al., 2012) and *in situ* protocol of (Rao et al., 2014) (62 °C for 5-10 min). In all these classical protocols, treatment of nuclei with SDS was followed by adding Triton X-100 and it was recommended to immediately move to the next step without incubating the nuclei with SDS/Triton X-100 (Dekker et al., 2002; Miele et al., 2006a), or to incubate with SDS/Triton X-100 only briefly (for 15 min at 37 °C) (Rao et al., 2014), or to incubate with SDS/Triton X-100 on ice for 10 min and then at 37 °C for 10 min (Kalhor et al., 2012).

Alternatively, the steps of extended incubations with SDS (for up to 1 h) and SDS/Triton X-100 (for up to 1 h) were introduced by (Tolhuis et al., 2002), and long-term incubations of nuclei with SDS and Triton X-100 for 1 h each were used even in the works of recent years (Sima et al., 2019; Golov et al., 2020; Vermeulen et al., 2020; Ulianov et al., 2021). Therefore, it is unclear whether extended chromatin treatments with SDS and Triton X-100 can affect DNA integrity.

To check this, we studied DNA preservation at various incubation regimens with SDS and Triton X-100. In addition, we studied whether pH of the 1X restriction buffer (RB) used to incubate nuclei with SDS/Triton X-100 affects the DNA integrity. It was found that prolong incubations with SDS and Triton X-100 and different pH values of the RB (pH 6.0 for the DpnII buffer and pH 7.9 for the NEB3 buffer were compared) do not impair the DNA integrity (Fig. S1A).

Thus, efficient washings of nuclei after lysis and inactivation of nucleases by heat before chromatin treatment allow both short and long processing of nuclei with SDS and Triton X-100 without DNA degradation and provide for extraction of histone proteins into a soluble fraction, as shown previously (Gavrilov, 2016).

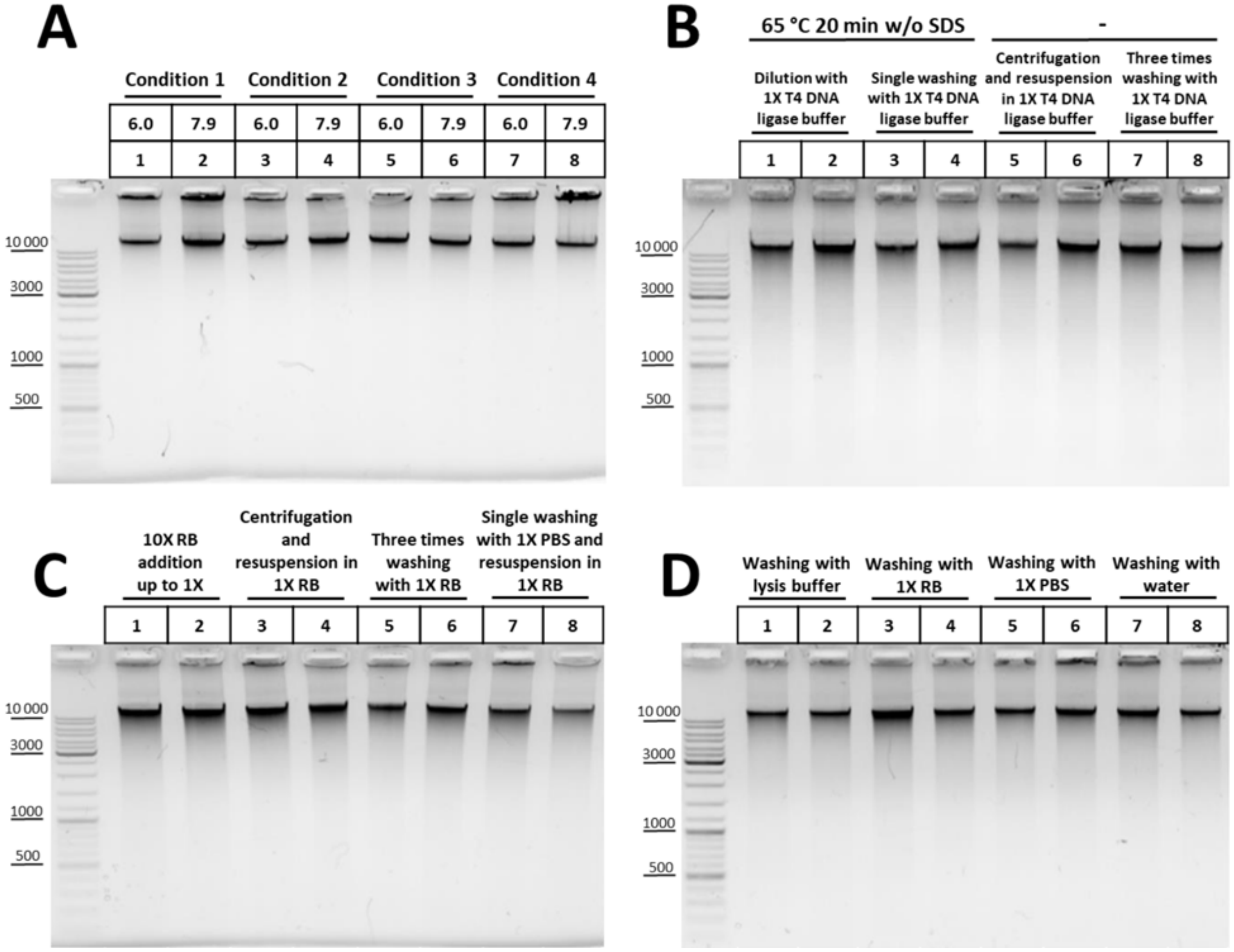

Figure S1. Preservation of the DNA integrity after SDS sequestration with Triton X-100 and after multiple washings of nuclei at various stages of the 3C procedure.

(A) Preservation of DNA integrity after SDS sequestration with Triton X-100. Cells were fixed, quenched, washed with 1X PBS as in Fig. 2A-C after quenching, then lysed with hypotonic lysis buffer #11 (see Table 2), and incubated on ice for 15 min. Nuclei were washed once with the lysis buffer and resuspended in 1X RB (DpnII buffer with pH 6.0 or NEB3 buffer with pH 7.9) or autoclaved water. Then SDS was added (0.1% final), the nuclei were incubated at different regimens in the presence of SDS, and Triton X-100 was added (1.8% final) subsequently. The incubation conditions were as follows: (i) two long sequential incubations at 37 °C combined with nuclease inactivation at 65 °C (10 min in 1X RB with SDS at 65 °C + 50 min with SDS at 37 °C + 1 h with SDS/Triton X-100 at 37 °C) (lanes 1 and 2); (ii) as described in (Miele et al., 2006b) (10 min in 1X RB with SDS at 65 °C + addition of Triton X-100 without incubation at 37 °C) (lanes 3 and 4); (iii) as described in (Rao et al., 2014) (10 min in water with SDS at 65 °C rather than 62 °C + 15 min in water with SDS/Triton X-100 at 37 °C) (lanes 5 and 6); and (iv) a combined variant: SDS treatment as described in (Miele et al., 2006b) (10 min with SDS at 65 °C) and SDS/Triton X-100 treatment as described in (Rao et al., 2014) (15 min with SDS/Triton X-100 at 37 °C), but both treatments in 1X RB rather than in water (lanes 7 and 8). Then 2 µl of a reconstructed DpnII storage buffer (10 mM Tris-HCl pH 7.4, 300 mM NaCl, 1 mM DTT, 0.1 mM EDTA, 500 µg/ml BSA, 50% glycerol) were added to a 45-µl of sample, and the nuclei were incubated at 37 °C for 1 h with agitation (imitation of the restriction reaction) and then heated at 65 °C for 20 min (imitation of RE inactivation in the *in*

situ protocol). After that, the reaction mixtures were diluted with a 1X ligation buffer to concentrations of ~10 mM NaCl, 0.01% SDS, and 0.2% Triton X-100; a T4 DNA ligase storage buffer (50 mM Tris-HCl, pH 7.5, 10 mM $MgCl_2$, 1 mM ATP, 10 mM DTT; 10 µl) was added; and the nuclei were incubated in the 1X T4 DNA ligase buffer at 16 °C for 30 min and at 22 °C for 30 min (imitation of the ligation reaction). Then Proteinase K (PrK), SDS, and EDTA were added to 0.2 mg/ml, 1%, and 30 mM, respectively, and the cross-links were reversed in hypotonic conditions at 56 °C overnight. DNA was extracted, precipitated, dissolved, treated with RNase A from bovine pancreas (bovine RNase A), and electrophoresed as in Fig. 2A-C. For lanes 5 and 6, 10X RB was added proportionally to reconstruct 1X RB before adding 2 µl of the DpnII storage buffer. In the upper row, 6.0 and 7.9 are the pH values of the restriction buffer used in the SDS/Triton X-100 treatments. Two replicates were done for each experimental condition.

(B) Washings of nuclei after the restriction reaction. Cells were fixed, quenched, washed with 1X PBS as in Fig. 2A-C after quenching, lysed, incubated in lysis buffer, and washed as in (A). The nuclei were resuspended in autoclaved water supplemented with 0.1% SDS and incubated at 65 °C for 10 min and subsequently with Triton X-100 (1.8% final) at 37 °C for 15 min. Then 10X RB (for DpnII) was added (1X final), and the nuclei were incubated at 37 °C for 1 h with agitation (imitation of the restriction reaction). Then the nuclei were incubated without SDS at 65 °C for 20 min lanes 1-4) or this step was omitted (lanes 5-8). The nuclei in 1X RB with SDS/Triton X-100 were diluted with a large volume of 1X T4 DNA ligase buffer without centrifugation (lanes 1, 2); or the nuclei were centrifuged, 1X RB with SDS/Triton X-100 was removed, and the nuclei were washed once with 1X T4 DNA ligase buffer and then resuspended in 1X T4 DNA ligase buffer (lanes 3, 4); or the nuclei were centrifuged, 1X RB with SDS/Triton X-100 was removed, and the nuclei were directly resuspended in 1X T4 DNA ligase buffer without washing (lanes 5, 6); or the nuclei were centrifuged, washed three times with 1X T4 DNA ligase buffer, and resuspended in 1X T4 DNA ligase buffer (lanes 7, 8). Then the nuclei were incubated at 16 °C for 30 min and 22 °C for 30 min with agitation (imitation of the ligation reaction) (lanes 1-8). Sample volumes were adjusted with 1X T4 DNA ligase buffer to 250 µl; PrK, SDS, and EDTA were added; cross-links were reversed as in (A); and DNA was extracted, precipitated, dissolved, treated with bovine RNase A, electrophoresed as in Fig. 2A-C.

(C) Washings of nuclei after chromatin treatment with SDS/Triton X-100. Cells were fixed, quenched, washed with 1X PBS as in Fig. 2A-C after quenching, lysed, incubated in the lysis buffer, and washed as in (A and B). The nuclei were resuspended in autoclaved water supplemented with 0.1% SDS and incubated at 65 °C for 10 min and subsequently with Triton X-100 (1.8% final) at 37 °C for 15 min. Then (i) 10X RB (DpnII buffer) was added to 1X to the nuclei in water/SDS/Triton-X100 (lanes 1, 2) without centrifugation; (ii) the nuclei were centrifuged, the supernatant containing SDS/Triton-100 was discarded, and the nuclei were resuspended in 1X RB (lanes 3, 4); (iii) the nuclei were centrifuged, the supernatant containing SDS/Triton-X100 was discarded, and the nuclei were washed three times with 1X RB and resuspended in 1X RB (lanes 5, 6); (iv) the nuclei were centrifuged, the supernatant containing SDS/Triton X-100 was discarded, and the nuclei were washed ones with 1X PBS and resuspended in 1X RB (lanes 7, 8). Then the nuclei were incubated at 37 °C for 1 h with agitation (imitation of the restriction reaction), centrifuged, washed three times with 1X T4 DNA ligase buffer as in (B), and resuspended in 1X T4 DNA ligase buffer. The nuclei were incubated at 16 °C for 30 min and 22 °C for 30 min with agitation (imitation of the ligation reaction). Sample volumes were adjusted with 1X T4 DNA ligase buffer to 250 µl; PrK, SDS, and EDTA were added; cross-links were reversed as in (A) and (B); and DNA was extracted, precipitated, dissolved,

treated with bovine RNase A, and electrophoresed as in Fig. 2A-C. Two replicates were done for each experimental condition.

(D) Washing of nuclei after cell lysis. Cells were fixed, quenched, washed with 1X PBS as in Fig. 2A-C after quenching, lysed, incubated in the lysis buffer as in (A,B,C), and washed with (i) the lysis buffer (lanes 1, 2), (ii) 1X RB (lanes 3, 4), (iii) 1X PBS (lanes 5,6), or (iv) autoclaved water (lanes 7, 8). The nuclei were resuspended in autoclaved water supplemented with 0.1% SDS and incubated at 65 °C for 10 min and subsequently with Triton X-100 (1.8% final) at 37 °C for 15 min. Then the nuclei were centrifuged, the supernatant containing SDS/Triton X-100 was discarded, and the nuclei were washed three times with 1X RB. The nuclei were incubated at 37 °C for 1 h with agitation (imitation of the restriction reaction), centrifuged, washed three times with 1X T4 DNA ligase buffer as in (B) and (C), and resuspended in 1X T4 DNA ligase buffer. The nuclei were incubated at 16 °C for 30 min and 22 °C for 30 min with agitation (imitation of the ligation reaction). Sample volumes were adjusted with 1X T4 DNA ligase buffer to 250 µl; PrK, SDS, and EDTA were added; cross-links were reversed as in (A)–(C); and DNA was extracted, precipitated, dissolved, treated with bovine RNase A, and electrophoresed as in Fig. 2A-C.

1.3 Washings of nuclei

Our preliminary experiments identified three important points when washing of nuclei often led to unexpected degradation of nuclear DNA in the 3C procedure. The points were: washing of nuclei after cell lysis, washing of nuclei after incubation with SDS/Triton X-100, and washing of nuclei after the restriction reaction prior to ligation. Therefore, we investigated how to properly wash the nuclei at these stages without impairing DNA integrity.

First, we investigated the washing of nuclei at the stage after restriction. In the in-nucleus ligation protocol, it was proposed to inactivate the RE by diluting the restriction reaction mixture with a 1X T4 DNA ligase buffer to a large volume (Nagano et al., 2013, 2015b) or first to precipitate the nuclei, then to discard most of the RE-containing supernatant, and to dilute the remainder volume with the 1X T4 DNA ligation buffer, thereby reducing the RE concentration and total ligation volume (Nagano et al., 2015a, 2017). These steps provide the mildest conditions for nucleus processing since the DNA ligation is performed in maximally intact nuclei, which have not been disturbed by heat and SDS upon RE inactivation (Nagano et al., 2015a, 2015b). Later, it was proposed to completely remove the RE-containing supernatant after the restriction reaction and to add a small volume of a 1X T4 DNA ligase buffer to the nuclear pellet in which DNA ligation was done (Sima et al., 2019) or to wash the nuclei once with 1X PBS and once with 1X T4 DNA ligase buffer (Flyamer et al., 2017). Alternatively, it was proposed for the *in situ* protocol that nuclei be washed from the RE with the 1X T4 DNA ligase buffer after inactivation of the RE without SDS at 65 °C for 20 min (Flyamer et al., 2017; Golov et al., 2020).

We decided to combine the above benefits (washing of nuclei from the RE with the 1X T4 DNA ligase buffer and not heating the nuclei without SDS at 65 °C for 20 min) in one step and investigated how these two options would affect the integrity of nuclear DNA after a thorough washing of nuclei. We checked the DNA integrity after (i) washing the nuclei with the 1X T4 DNA ligase buffer after their incubation without SDS at 65 °C for 20 min, as in the *in situ* protocol, and (ii) washing the nuclei without incubation them without SDS at 65 °C for 20 min. We observed that, in both cases, multiple washings of nuclei with the 1X T4 DNA ligase buffer did not lead to a loss of DNA

integrity (Fig. S2B). Peter Fraser's team were the first to omit RE inactivation by heating at 65 °C, which is harmful for the nuclear structure (Nagano et al., 2013, 2015a, 2015b, 2017).

Next, we studied the washing of nuclei after chromatin treatment with SDS/Triton X-100 and before the restriction digestion step. Extraction of uncross-linked proteins in the 3C procedure usually occurs in the presence of certain amount of SDS, which is thereafter sequestrated with Triton X-100. However, even sequestrated with Triton X-100, SDS is able to hamper the RE function at high concentrations (Louwers et al., 2009), only few REs can work and in these condition. For example, BamHI is able to tolerate no more than 0.15% SDS (Louwers et al., 2009). In the dilution protocol, CpG methylation-insensitive HindIII, EcoRI, BglII (6-bp cutters), DpnII, NlaIII, and Csp6I (CviQI) (4-bp cutters) are recommended for digestion of cross-linked chromatin in the presence of SDS/Triton X-100 since they can tolerate the conditions of 0.3% SDS sequestered with 1.8% Triton X-100 (Splinter et al., 2012; van de Werken et al., 2012). One more list of the REs that are able to tolerate sub-optimal buffer conditions with considerable SDS/Triton X-100 concentrations also included EcoRI, HindIII, and BglII along with XhoI, AciI, and BsrGI and was proposed by another research team (Naumova et al., 2012). However, restriction in sub-optimal buffer conditions may apparently be far from efficient. Dialyzing the lysed nuclei to reduce the SDS concentration was proposed as an option to achieve full activity of RE enzymes (Splinter et al., 2004). It was proposed in a recent works to remove the SDS/Triton X-100-containing supernatant prior to the restriction reaction (Flyamer et al., 2017; Golov et al., 2020) or to wash the nuclei once with the 1X RB after removing SDS/Triton X-100 (Flyamer et al., 2017). Thus, reducing the SDS/Triton X-100 concentration in the reaction can improve chromatin digestion and would make it possible to expand the repertoire of suitable REs. We hypothesized that a thorough washing of nuclei from SDS/Triton X-100 may be more efficient than removing the supernatant or doing a single washing. However, washing can lead to DNA degradation in this case, as was evident from our preliminary experiments (see Reversion of Cross-links and Isolation of the 3C Library). We therefore studied how washing at this stage would affect DNA integrity. We found that nuclei can be centrifuged and washed at this stage at least three times with 1X RB and even with 1X PBS and that this washing does not lead to DNA degradation in the nuclear fraction (Fig. S2C).

Thus, the SDS/Triton X-100 mixture can be efficiently washed off after chromatin treatment without disturbing DNA integrity. Ilya Flyamer was the first to propose removing SDS/Triton X-100 mixture prior to the restriction reaction and washing the nuclei with 1X RB after it (Flyamer et al., 2017).

Finally, we studied the washing of nuclei after cell lysis. As shown in Fig. 2A-C, cell lysis conditions can significantly affect the final quality of the 3C library. Hence, the stage of washing the nuclei from the remnants of the cytoplasm might apparently be very important. Several options were described in the literature for washing after lysis, including washing with the lysis buffer (Rao et al., 2014), 1X RB (Lieberman-Aiden et al., 2009; Ulianov et al., 2016), and even 1X PBS (Stadhouders et al., 2013a). We therefore assessed whether the nature of the washing solution at this stage affects the integrity of nuclear DNA. We investigated the effects of the lysis buffer, 1X RB, 1X PBS, and autoclaved water. None of the washing solutions was found to affect DNA quality (Fig. S2D).

Thus, nuclei can be washed with various washing solutions and even with water after the step of cell lysis and their washing results in no detectable DNA degradation at the subsequent stages of the procedure. Aiden's team proposed the step of washing the nuclei with a hypotonic lysis buffer (Rao et al., 2014).

To summarize, the following steps can be used in the 3C protocol based on the results of our washing-related experiments: (i) multiple washes of nuclei with a 1X T4 DNA ligase buffer after the stage of the restriction reaction (preparation for the ligation reaction); (ii) multiple washes of nuclei from the SDS/Triton X-100 mixture with 1X RB after the stage of chromatin treatment with SDS/Triton X-100 (preparation for the restriction reaction); and (iii) washing with virtually any washing solution after cell lysis.

According to our observations, washing with the 1X T4 DNA ligase buffer after the restriction step and 1X RB after chromatin treatment with SDS/Triton X-100 does not lead to DNA degradation and are possible to perform when only two conditions are met in 3C procedure. First, nuclei thoroughly washed from cytoplasm remnants with the lysis buffer (or another washing solution) (absolutely essential) and, second, ii) residual DNA endonucleases are inactivated by heat at the stage of chromatin preparation for digestion with a RE (auxiliary significance). If both steps are followed, no DNA degradation is observed in subsequent steps. The step of washing from cytoplasm remnants is the most important. The composition of the lysis buffer is also the important factor. For example, with the hypotonic lysis buffer rich in non-ionic detergents, DNA degradation does not occur even with subsequent chromatin treatment at 37 °C (Fig. 2B). This may be due to better extraction of the nucleoplasm under hypotonic conditions (Méndez and Stillman, 2000; Golov et al., 2015). After lysis in a hypotonic lysis buffer, cytoplasmic remnants can be washed off with any washing solution, including water, without impairing DNA integrity.

RCF used during centrifugation to pellet the nuclei is also of importance. It was proposed not to centrifuge nuclei at a RCF higher than 4,000–5,000g, since both 8,000 and 12,000g resulted in broken, sheared nuclei of plant cells (Louwers et al., 2009). We never used centrifugation at a RCF exceeding 5,000g in our experiments. In contrast, a RCF of 2,500g was generally sufficient to form a dense pellet of nuclei. The only step where a RCF above 500-1,000g was found to be excessive is washing the nuclei with 1X RB after their SDS/Triton X-100 treatment; i.e., the pellet becomes too dense and is poorly resuspended by pipetting.

It was recommended additionally to limit the number of washing steps since more than two washing steps easily result in a low yield and a high proportion of damaged nuclei according to observations by (Louwers et al., 2009). According to our observations, centrifugation at low RCFs does not allow the nuclei to concentrate efficiently on the bottom of the tube, thus potentially causing losses of nuclei during washes. As to damage to nuclei during washing, we showed that washing can be performed for at least three times at different stages without accompanying DNA degradation. The loss of DNA from nuclei rather depends on how sternly chromatin has been processed with heat and SDS (see Fig. 4), but not on the number of washes.

1.4 Optimization of TaqMan PCR conditions

Before conducting the main experiments, we optimized the qPCR conditions to achieve a maximum efficiency in detecting the ligation products. The experimental design of primers and probes for testing the *RpII* locus is shown in Fig. S2A. The results of amplification used to estimate the amount of the uncut DpnII site in the *RpII* locus are shown in Fig. S2B. The presence of the DpnII restriction site at this position of the *RpII* locus in our S2 cell line was verified by sequencing (Fig. S2C).

To increase the sensitivity of the assay and to prevent nonspecific amplification and amplification of primer dimers, which is often observed in SYBR green qPCR (Ruiz-Villalba et al., 2017), we chose a TaqMan probe-based detection system. The primer and TaqMan probe concentrations (Fig. S2D), the PCR mode (three or two steps), and the reaction volume were optimized to achieve the maximum sensitivity of TaqMan PCR. The optimal primer concentration was determined to be 0.3 pM/µl; the concentration of the TaqMan probes was chosen to be 0.3 pM/µl since this concentration provided a good balance between costs and qPCR performance (Fig. S2D). A two-step PCR mode was chosen since it was slightly more efficient than the classical three-step mode. Primer annealing and extension are carried out at the same temperature in this mode. These conditions increased the efficiency of TaqMan PCR by 2.5 Ct on average (Table S2). A halving of the reaction volume to 5 µl increased the qPCR efficiency to 4.5 Ct. However, reactions in a 5-µl volume were not always successful on 96-well plates, and we therefore chose a reaction volume of 10 µl.

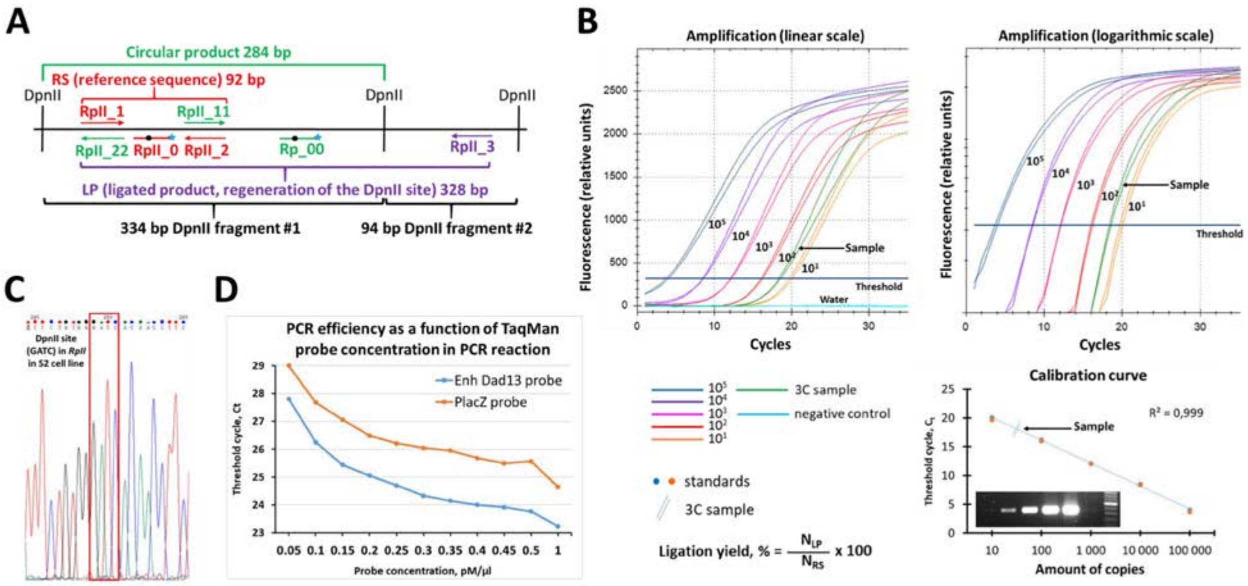

Figure S2. Demonstration of the experimental system for quantifying the ligation frequencies in the *RpII* locus.

(A) The experimental design of primers and probes to estimate the ligation efficiency (regeneration of the DpnII site) and circularization of the ligation product in the *RpII* locus. The frequency of the intact (uncut and religated) DpnII site was estimated before and after ligation using PCR-stop analysis with a combination of the RpII_1, RpII_2, and RpII_3 primers and RpII_0 probe against a calibration curve, which was constructed using the PCR product obtained with the RpII_1 and RpII_3 primers. The frequency of regeneration of the original DpnII site was calculated as a difference between the percentage of the intact (uncut) restriction site in samples before and after ligation and was designated as ligation yield. The amount of the circularized product was estimated using the RpII_11, RpII_22 primers and Rp_00 probe. (B) An example experiment with primers used to amplify the uncut DpnII site. Fluorescence growth curves (linear and logarithmic scales) are at the top. Green curves show the results for the 3C template; blue curves, for the negative control (water or 50 ng of S2 cell genomic DNA digested with DpnII and randomly ligated in solution); and colored curves of different colors, for the DNA standards. A standard curve and the electrophoresis picture of

the amplified standards are presented at the bottom right. The equation used to calculate the ligation yield (regeneration of the original restriction site, target ligation) is at the bottom left. N_{RS} is the amount of the reference sequence and N_{LP} is the amount of the ligated (intact, uncut) product. (C) Chromatogram of sequencing the DpnII site of the *RpII* locus in the S2 cell line. (D) Ct as a function of TaqMan probe concentration in the PCR mixture. The relationship was studied for two probes using 10 ng of *Drosophila* genomic DNA template of fly line with the reporter cassette LexAop-hsp70-lacZ^{Dad} inserted in the endogenous *Dad* promoter region about 5 kb upstream of the mapped *Dad* enhancer (Shidlovskii et al., 2021). The Enh Dad13 probe with the Enh Dad13 and Enh Dad13_test primers and the PlacZ probe with the PlacZ and PlacZ_test primers were used, respectively, to estimate efficiency of amplification (see Table S8).

Table S2. Comparison of the TaqMan real-time PCR conditions

Parameter	Basic conditions	Maximum efficiency	Maximum reliability (reproducibility)
Probe concentration, pM/µl	0.16	0.5-1	0.3
Primer concentration, pM/µl	0.4	0.1-0.2	0.3
Reaction volume, µl	20	5 µl	10
Number of PCR steps	3 (94° 10 sec, 60° 25 sec, 72° 35 sec)	2 (94° 10 sec, 60° 1 min)	2 (94° 10 sec, 60° 1 min)
Gain in PCR, Ct	0	4.5	2.5

1.5 Reversion of Cross-links and Isolation of the 3C Library

In this part, we discuss the roles of EDTA, ionic strength, dilution, temperature and the composition of the extraction buffer (EB) in isolation of an intact 3C library.

1.5.1 The roles of EDTA, ionic strength, and dilution in maintaining DNA integrity in the 3C procedure

According to our observations, the critical stages of the 3C procedure that are important for maintaining nuclear DNA integrity are cell lysis, chromatin treatment with heat in the presence of SDS/Triton X-100, and the transition from chromatin digestion to DNA ligation. To get insights into the causes of DNA degradation in the 3C protocol, we investigated in detail exactly at what steps and in what conditions DNA degradation occur (Table S3). To prevent DNA degradation, we tried to use EDTA during reversion of cross-links.

Table S3. Influence of performing or omitting 3C protocol steps on DNA integrity

#	Step of protocol[0]	Performing or omitting the step							
1	Cell lysis (isotonic, #2 in Table 2)	+*	+	+	+	+	+	+	+[1]
2	Washing of nuclei #1 (1X PBS)	–**	+	+	+	+	–	–	+[5]
3	Heat treatment of nuclei at 65 °C[2]	+	–	–	+	+	+	+	+[6]
4	Washing of nuclei #2 (1X PBS)	–	–	–	–	+	–	–	–
5	Resuspension of nuclear pellet in 1X RB	–	–	+	+	+	+	+	+
6	Restriction digestion (3h at 37 °C)	–	–	–	–	–	+	+	+
7	Washing of nuclei #3 (1X T4 DNA ligase buffer)	–	–	–	–	–	–	+	+
8	Dilution of nuclei in 1X RB with large volume of<br><br>1X T4 DNA ligase buffer	–	–	–	–	–	+	–	–
9	Ligation reaction (at 16 °C O/N)	–	–	–	–	–	+	+	+
10	Addition of EB[3] and incubation at 56 °C O/N	+	+	+	+	+	+	+	+
	Result[4] (#1-8)	1	2	3	4	5	6	7	8

*- Step is performed, ** - step is omitted, O/N - overnight

[0] - Steps of 3C procedure that are critical for DNA integrity including washings between steps

[1] - Hypotonic lysis buffer (#11, Table 2)

[2] - 10 min at 65 °C with 0.1% SDS, + 1 h at 37 °C with 0.1% SDS/1.8% Triton X-100

[3] - Contains 0.2 mg/ml PrK, 1% SDS, and different amounts of EDTA

[4] - The following results were obtained:

1. DNA degradation in all range of EDTA concentrations in EB (0-50 mM)
2. DNA intact at EDTA concentration above 35 mM in EB

3. DNA intact at EDTA concentration above 30 mM in EB, but the result is unstable
4. DNA intact at EDTA concentration above 25 mM in EB
5. Degraded DNA without RNA at 30 mM EDTA in EB
6. DNA degradation at 30 mM EDTA in EB
7. Intact DNA at 30 mM EDTA in EB or without EDTA
8. Intact DNA at 30 mM EDTA in EB or without EDTA

[5] - Washing can be performed with 1X PBS or a hypotonic buffer

[6] - Heat treatment of chromatin in water with SDS at 65 °C/37 °C for 10 min

First, when step #2 was not done after lysis in isotonic conditions, then DNA degradation was observed over the entire range of EDTA concentrations in EB (0-50 mM) despite heating the nuclei at 65 °C (step #3) (Fig. S3A, Table S3, column 1) to inactivate nucleases (Louwers et al., 2009). When step #2 was done, but step #3 was omitted intact DNA was obtained at high EDTA concentrations in EB and the result was unstable at lower EDTA concentrations (Fig. S3B,C; Table S3, column 2 and 3, respectively). When both steps #2 and #3 were combined, the result was stable and DNA remained intact over a wider EDTA concentration range (Fig. S3D; Table S3, column 4).

These findings suggest that the washing of nuclei and their heating at 65 °C both contribute to maintaining DNA integrity. However, the washing of nuclei plays a leading role and the step of chromatin heating at 65 °C plays an auxiliary role. At the same time, EDTA concentrations above 25 mM in EB were still required for preserving DNA integrity during reversion of cross-links. When EDTA was absent from EB, then DNA degradation occurred regardless of the type of lysis buffer (Fig. S3E) (the nuclei were not washed with 1X PBS after cell lysis), the concentration of the detergents in the lysis buffer (Fig. S3F), and the duration of chromatin heating at 65 °C (Fig. S3G).

We assumed that residual metal-dependent nucleases (Lechardeur et al., 1999; Yang, 2011; Kawane et al., 2014; Fujiwara et al., 2017) might be responsible for DNA degradation and that, starting from a certain EDTA concentration, conditions are created in which all divalent cations are bound and DNA remains intact during reversion of cross-links. It was also shown that treatment of nuclei with a hypotonic, but not isotonic buffer leads to an efficient release of nucleoplasmic proteins (Méndez and Stillman, 2000; Golov et al., 2015). Hence, we assumed that nucleases pass into solution probably at the step of prolonged incubation to reverse cross-links in hypotonic conditions (250 µl of the hypotonic 1X T4 DNA ligase buffer) and digestion of the nuclei with PrK in above cases 1-4 (Table S3), but not at early steps of the protocol since all previous steps were performed in isotonic conditions. We chose an EDTA concentration of 30 mM in EB to prevent DNA degradation during reversion of cross-links.

Second, since we detected a release of DNA from nuclei after heating chromatin at 65 °C (see Nucleoplasm Release and Chromatin Treatment with Heat), we tried to wash the released DNA off the nuclei with 1X PBS (Table S3, column 5). Although, steps #2 and #3 were carried out, RNA was unexpectedly not detected in isolated DNA samples, while usually present in 3C libraries after reversion of cross-links (Fig. S3H). Traces of DNA degradation were observed in some of the samples (Fig. S3H). When the samples were treated with bovine RNase A, DNA was completely degraded (Fig. S3I and Treatment of the 3C library with RNases in supplementary materials). We concluded that, after isotonic lysis and steps #2 and 3, also done in isotonic conditions, a washing of the nuclei with an isotonic buffer (1X PBS) results in loss of DNA integrity, RNA decay, and

subsequent DNA degradation at the stages of cross-links reversion and treatment with bovine RNase A. We assume that DNA degradation similarly does not occur in isotonic conditions at the initial stages in this case, but occurs as nucleases are released in hypotonic conditions during reversal of cross-links.

Third, we observed that when steps #2 was omitted and step #3 done after lysis in isotonic conditions, then dilution of the restriction reaction mixture with a large volume of the 1X T4 DNA ligase buffer after RE inactivation to allow subsequent DNA ligation (step #8) prevents DNA degradation in the absence and in the presence of EDTA in EB (Fig. S3 J,K; Table S3, column 6). Conversely, a washing of the nuclei with the 1X T4 DNA ligase buffer after the restriction reaction (step #7) provokes DNA degradation both in the presence and in the absence of EDTA in EB (Fig. S3 L,M; Table S3, column 7). We hypothesize that, in the latter case, nucleases passed into solution probably after a sequential fast washing with the 1X T4 DNA ligase buffer, as the nuclei were finally suspended in a small volume (45 µl) of the same buffer for the DNA ligation reaction. DNA degradation probably occurred during incubation of the ligation reaction, but not at the stage of cross-links reversion, where 30 mM EDTA was added to EB. Resuspension of nuclei in a large volume of the 1X T4 DNA ligase buffer leads to a similar nuclease release during ligation, but DNA degradation is prevented even in the absence of EDTA as a result of high dilution (~1.5 ml).

We concluded that, when the washing of nuclei was not done properly, dilution of the restriction reaction mixture with a large volume of the 1X T4 DNA ligase buffer prior to DNA ligation may be a way to preserve the integrity of the 3C library. This step is commonly used in the dilution ligation protocol (Dekker et al., 2002; Tolhuis et al., 2002; Lieberman-Aiden et al., 2009; Comet et al., 2011; Stadhouders et al., 2013a; Ulianov et al., 2016; Vermeulen et al., 2020) and can be introduced in any protocol, if necessary.

An isotonic lysis buffer was used for cell lysis in the above-mentioned experiments. When we changed to a hypotonic buffer and combined steps #2 and #3, we observed that step #7 no longer led to DNA degradation (Table S3, column 8) both in the presence (Fig. S1 A-D) and in the absence of EDTA in EB (Table S5, see also Fig. S3F). In addition, the hypotonic lysis buffer was the only buffer that ensured DNA integrity in all experimental conditions (Fig. 2A-C); allowed chromatin treatment at 37 °C instead of 65 °C, as is more favorable for ligation (Fig. 5 C,D); and made it possible to wash the nuclei with 1X RB from DNA released from nuclei after step #3 (Fig. S1 C,D).

Thus, lysis with a hypotonic buffer appears to be more efficient. The use of a hypotonic lysis buffer in combination with a step of washing of nuclei ensures preservation of DNA integrity throughout the protocol, efficient nuclease removal and avoids harsh chromatin treatment at 65 °C in favor of 37 °C.

It was previously reported that a failure of cell lysis hampers the digestion efficiency, possibly by limiting access of the RE to the DNA and it was proposed to titrate SDS and Triton X-100 to compensate for poor cell lysis (Splinter et al., 2012). Our results suggest that insufficient cell lysis in an isotonic buffer can lead to degradation of nuclear DNA during subsequent steps of the 3C procedure and can only be overcome with EDTA. Hence, if lysis of the remaining non-lysed cells occurs at the stage of treating the nuclei with SDS and Triton X-100 as suggested in (Splinter et al., 2012), then nucleases released from non-lysed cells will disrupt the integrity of nuclear DNA. This may lead to a loss of the desired interaction profile or decrease the interaction frequency. Therefore,

titration of SDS and Triton X-100 cannot compensate for poor cell lysis since nucleases released from non-lysed cells will cause chromatin degradation.

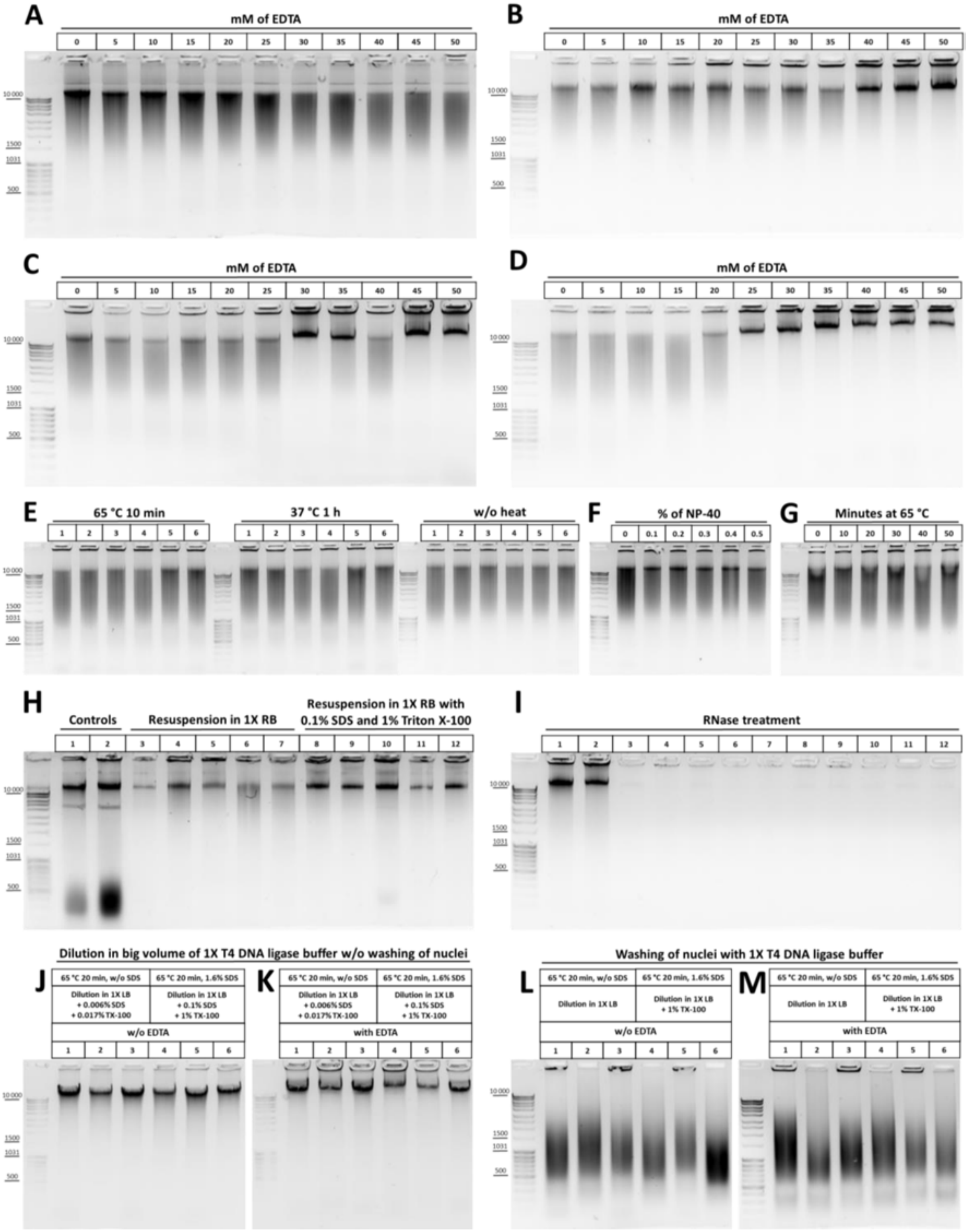

Figure S3. Influence of different steps of the 3C protocol on the DNA integrity (see also Table S3).

(A) DNA integrity after steps #1 and 3 (step #2 was omitted). Cells were fixed, quenched, washed with 1X PBS as in Fig. 2A-C after quenching, lysed with isotonic lysis buffer #2 (see Table 2), and incubated on ice for 15 min. The nuclei were centrifuged and resuspended without washing in 1X RB for DpnII, SDS was added to 0.1%, the nuclei were heated at 65 °C for 10 min + 37 °C for 50 min, Triton X-100 was added to 1.8%, and the nuclei were incubated at 37 °C for 1 h. Then the nuclei were centrifuged, and EB containing different amounts of EDTA was added to the nuclear pellet as in Fig. 2A-C. Cross-links were reversed at 56 °C overnight. DNA was extracted, precipitated as in Fig. 2A-C, dissolved in 25 µl of 10 mM Tris-HCl (pH 8.0), and electrophoresed as in Fig. 5A-D. One of the three replicate experiments is shown.

(B) DNA integrity after steps #1 and 2 (step #3 was omitted). The experiment was done as in (A). After washing with 1X PBS, the nuclei were centrifuged, the step of heating chromatin in the presence of SDS/Triton X-100 was omitted, and EB containing different amounts of EDTA was added to the nuclear pellet.

(C) DNA integrity after steps #1, 2, and 5 (stepы #3 and 4 were omitted). The experiment was done as in (B) except that the nuclear pellet was resuspended in 1X RB before adding EB.

(D) DNA integrity after steps #1, 2, 3, and 5 (step #4 was omitted). The experiment was done as in (C) except that the nuclei were heated in the presence of SDS and Triton X-100 as in (A).

(E) Effect of EDTA in EB on the DNA integrity when various lysis buffers were used. The experiment was done as in Fig. 2A-C except that the nuclei were not washed with 1X PBS after cell lysis and EB did not contain EDTA.

(F) Effect of EDTA in EB on the DNA integrity when different amounts of non-ionic detergents were used. The experiment was done as in (D) except that cells were lysed in isotonic lysis buffer #2 containing different amounts of NP-40 and that EB did not contain EDTA.

(G) Effect of EDTA in EB on the DNA integrity when the duration of chromatin treatment at 65 °C was varied. The experiment was done as in (D) except that EB did not contain EDTA.

(H, I) DNA integrity after steps #1, 2, 3, 4, and 5. The experiment was done as in (A) except that the nuclei were washed two times with 1X PBS: once after incubation in the lysis buffer and once after heating chromatin in the presence of SDS/Triton X-100, respectively. After the second washing with 1X PBS, the nuclei were resuspended in 1X RB supplemented with 0.1% SDS + 1% Triton X-100 to reconstruct the conditions of restriction digestion in the presence of SDS/Triton X-100, and EB (additionally containing 30 mM EDTA) was added as in Fig. 2A-C. Cross-links were reversed as in (A). Then DNA was extracted, precipitated as in Fig. 2A-C, dissolved in 25 µl of 10 mM Tris-HCl (pH 8.0), and electrophoresed as in Fig. 5A-D. In (I), dissolved DNA from (H) was treated with bovine RNase A as in Fig. 2A-C and electrophoresed as in Fig. 5A-D. In control samples, EB was added immediately after heating the nuclei with SDS/Triton X-100 without washing with 1X PBS. Control samples were prepared in duplicate, and five replicates were done for each experimental condition.

(J-M). Effect of dilution of nuclei with a large volume of 1X T4 ligase buffer or repeated washing of nuclei with 1X ligase buffer. (J, K) DNA integrity after steps #1, 3, 5, 6, and 8 (steps #2, 4, and 7 were omitted). (L, M) DNA integrity after steps #1, 3, 5, 6, and 7 (steps #2, 4, and 8 were omitted).

The experiment was done as in (A) including the stages of heating chromatin in the presence of SDS and Triton X-100. After that nuclei were centrifuged, 9/10 of the supernatant was discarded, and the nuclei were resuspended in 1X RB for DpnII (final NaCl concentration ~90 mM). The DpnII storage buffer (2 µl) was added, and the samples were incubated at 37 ° C for 3 h with agitation (imitation of the restriction reaction). Then the samples were subdivided into two groups. In the first group (Lanes 1, 2, 3 of J-M), the nuclei were incubated at 65 °C for 20 min without adding SDS (imitation of RE inactivation in the *in situ* protocol) (Rao et al., 2014). In the second group (Lanes 4, 5, 6 of J-M), the nuclei were incubated at 65 °C for 20 min in the presence of 1.6% SDS (imitation of RE inactivation in the dilution protocol) (Dekker et al., 2002; Tolhuis et al., 2002; Lieberman-Aiden et al., 2009; Comet et al., 2011; Stadhouders et al., 2013b; Ulianov et al., 2016; Vermeulen et al., 2020). Then the nuclei were diluted with 1X T4 DNA ligase buffer to NaCl, SDS, and Triton X-100 concentrations of 5.89 mM, 0.006%, and 0.117% (~1.5 ml), respectively (J, K, Lanes 1, 2, 3) or 1X T4 DNA ligase buffer to NaCl and SDS concentrations of 5.89 mM and 0.1% (~1.5 ml), respectively, and Triton X-100 concentration was adjusted to 1% as in the dilution protocol (J, K, lanes 4, 5, 6). Alternatively to dilution, the nuclei were washed twice with 1X T4 DNA ligase buffer (100 µl) (Flyamer et al., 2017; Golov et al., 2020) (L, M) and resuspended in 1X T4 DNA ligase buffer (L, M, lanes 1, 2, 3) or 1X T4 DNA ligase buffer supplemented with 1% Triton X-100 as in the *in situ* protocol (L, M, Lanes 1, 2, 3) (Rao et al., 2014). Then 2 µl (20 µl in the case of dilution) of T4 DNA ligase storage buffer and ATP (to 1 mM) were added, and the samples were incubated at 16 °C overnight with agitation (imitation of the ligation reaction). Volumes of the undiluted samples were adjusted with 1X T4 DNA ligase buffer to 250 µl; PrK and SDS were added to the samples as in Fig. S1A except that 30 mM EDTA was added only in experiments (K) and (M) and not in (J) and (L), respectively; cross-links were reversed at 56 °C overnight; and DNA was extracted, precipitated, dissolved, treated with bovine RNase A, and electrophoresed as in Fig. 2A-C. Three replicates were done for each experimental condition.

1.5.2 The roles of the temperature and composition of the extraction buffer in maintaining DNA integrity in the 3C procedure

Methods based on DNA fixation with formaldehyde (FA) require reversion of cross-links between FA and amino groups of DNA bases and proteins to obtain protein-free DNA (Hoffman et al., 2015). In ChIP or 3C protocols, the reversion of cross-links is usually performed at 65 °C overnight and is combined with treatment with PrK, an enzyme from the free-living mesophilic fungus *Engyodontium album*. There are indications in the literature that a decrease in incubation temperature (to 50 °C) during treatment with PrK increases the purity of extracted DNA (Qamar et al., 2017). At the same time, it is widely believed that PrK is most active in a temperature range of 55-65 °C since these temperatures are thought to promote unfolding of substrate proteins. However, to our surprise, we and others did not find any reference to the temperature at which PrK exhibits maximum performance with respect to proteins. In contrast, the highest PrK activity was observed in a range from 25 to 40 °C (Bajorath et al., 1988), which roughly corresponds to the temperatures at which fungi of the genus *Engyodontium* grow (Bajorath et al., 1988). In Ca^{2+}-free buffer conditions (in the presence of 20 mM EDTA), higher temperatures induced a rapid drop of PrK activity and the stability of PrK increased when the incubation temperature was decreased (Bajorath et al., 1988). In line with these concepts, it is of interest to investigate reversion of cross-links in the 3C procedure at temperatures that are below 65 °C and allow PrK to retain its activity longer.

Another important issue is the composition of the DNA EB, in which digestion of proteins with PrK occurs. Since PrK is active over a wide range of pH (7.5-12.0) (Ebeling et al., 1974), we chose pH 8.0 as the pH most often used for DNA buffers. The concentration of SDS, another essential component of DNA isolation buffers, was chosen to be 1% since this SDS concentration was shown to cause no decrease in PrK activity for at least 2 hours at 37 °C at pH 8.0, but to stimulate PrK activity by several times (Hilz et al., 1975). However, when PrK was added to a concentration of 0.2 mg/ml in the same conditions, SDS caused a dramatic drop in PrK activity up to its complete loss within 2 hours at 50 °C (Samal et al., 1991). This implies that a temperature lower than 50 °C is recommended for a maximum efficiency and preservation of PrK activity. Nevertheless, we decided that two hours at 50 °C or at 56 °C should be enough to digest most of the protein when PrK is added to a high concentration of 0.2 mg/ml.

In order to study how long it takes to reverse the cross-links, the rate of cross-link reversal in protein–DNA complexes resulting from FA fixation was calculated based on the literature data available for 4, 23, 37, and 47 °C (Kennedy-Darling and Smith, 2014). The time and rate were calculated accordingly for 50, 56 and 65 °C (Fig. S4E and Table S4). Then we took advantage of our previous finding (see Cell fixation, FA inactivation and nucleus storage) that a commercial column DNA isolation kit does not isolate cross-linked DNA, but does isolate it after cross-link reversal, thus providing an indicator of reversion. As expected, cross-linked DNA (0.5-2% FA) was not isolated with the column kit after 10-min incubation at 56 °C (the time and temperature recommended for genomic DNA isolation by the manufacturer) (Fig. S4A), but was readily isolated with phenol/chlorophorm (Ph/Chl) extraction (Fig. S4D). After overnight incubation at 56 °C, isolation of cross-linked DNA was successful both with the column and with Ph/Chl extraction (Fig. S4B,C). This indicates that reversion of cross-links was complete. We observed additionally that a high FA concentration (2%) leads to a low DNA yield even a loss of DNA after Ph/Chl extraction (Fig. S4B,D). This can be a consequence of cell over-fixation occurring at the given FA concentration within 10 min.

Table S4. Reaction rate, time, and temperature required for a complete release of DNA from DNA-protein complexes

Temperature, °C	Reaction rate,<br>% protein-free DNA/h	Time, h
4	0.3	333.33
23	1.10	90.90
37	2.20	45.45
47	4.40	22.73
50	5.23	19.12
56	7.56	13.23
65	13.13	7.62

Next, we investigated the reversion of cross-links in the presence of PrK at 50 °C. We found that incubation at 50 °C for 19 h was sufficient to obtain intact DNA (Fig. S4F). The same result was obtained after incubation at 56 °C for 13.5 h (not shown). As expected, DNA of better spectrophotometric purity was obtained after extraction with Ph/Chl, but not with a column-based DNA extraction kit. The DNAs isolated at 50 °C and at 65 °C with Ph/Chl had the same 260/280 and 260/230 ratios.

Thus, these results suggest that temperatures milder (50, 56 °C) than standard 65 °C can be efficiently used in combination with PrK treatment to reverse the cross-linked DNA in the 3C procedure and that DNA of better purity is obtained after extraction with Ph/Chl, but not with a column sorption technique.

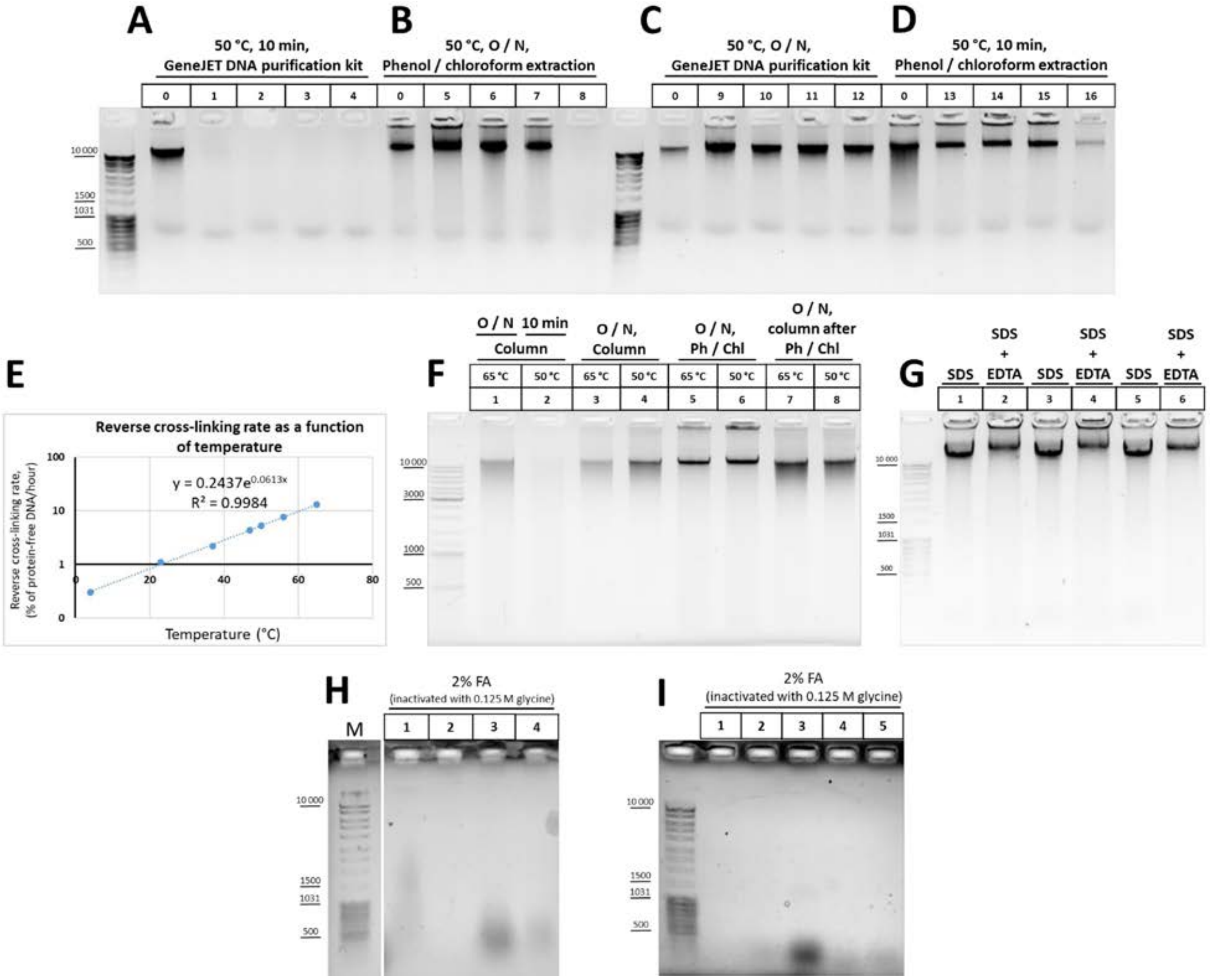

Figure S4. Reversion of cross-links at different temperature and a composition of the extraction buffer.

(A-D) Cells were harvested, washed with 1X PBS, left unfixed or fixed with FA at different FA concentrations in 1X PBS at room temperature for 10 min, quenched with equimolar glycine for 5 min at RT, and washed with 1X PBS. Then EB was added as in Fig. 2A-C, and the cells were incubated to reverse the cross-links as indicated at the top. DNA was extracted, precipitated, dissolved, treated with bovine RNase A, and electrophoresed as in Fig. 2A-C. Lane 0, unfixed cells (control); lanes 1, 5, 9, 13, 0.5% FA; lanes 2, 6, 10, 14, 1% FA; lanes 3, 7, 11, 15, 1.5% FA; lanes 4, 8, 12, 16, 2% FA. One of the three replicate experiments is shown.

(E) Temperature dependence of the cross-link reversion rate. The cross-link reversion rate as a function of temperature was fit to an exponential. For details, see Table S4.

(F) Cells were fixed, quenched, washed with 1X PBS after quenching as in Fig. 2A-C. Then the cells were processed as follows:

(1) The cells were resuspended in 1X PBS, mixed 1:1 with the lysis buffer of a GeneJET Genomic DNA purification kit (Thermo, #K0721), incubated in the presence of PrK at 65 °C for 19 h, cooled, and treated with RNase A at room temperature for 10 min. DNA was isolated using a standard column-based protocol (plus control) and electrophoresed as in Fig. 2A-C). One of the three replicate experiments is shown.

(2) The cells were treated as in (1) except that the nuclei were incubated at 50 °C for 10 min (minus control).

(3) The cells were resuspended in 1X T4 DNA ligase buffer supplemented with SDS, EDTA, and PrK at concentrations as in Fig. S1A; incubated at 65 °C for 19 h; cooled; treated with RNase A at room temperature for 10 min; and diluted 1:1 with the lysis buffer of a GeneJET Genomic DNA purification kit. DNA was isolated using a standard column-based protocol and electrophoresed as in Fig. 2A-C.

(4) The cells were treated as in (3) except that DNA was processed at 50 °C for 19 h.

(5) The cells were treated as in (3) except that DNA was extracted with Ph/Chl, precipitated, and dissolved as in Fig. 2A-C.

(6) The cells were treated as in (5) except that DNA was processed at 50 °C for 19 h.

(7) The cells were treated as in (3) except that DNA was extracted with Ph/Chl, mixed 1:1 with the lysis buffer of a GeneJET Genomic DNA purification kit, and isolated using a standard column-based protocol.

(8) The cells were treated as in (7) except that DNA was processed at 50 °C for 19 h.

One of the three replicate experiments is shown.

(G) Cells were fixed, chromatin was treated, and nuclei were diluted as described in Fig. S3 J, K. After incubation in the presence of 20 µl of T4 DNA ligase storage buffer and 1 mM ATP at 16 °C overnight (imitation of the ligation reaction), SDS, EDTA, and NaCl were added as indicated in Table S5 and cross-links were reversed in the presence of PrK at 56 °C overnight. DNA was extracted with Ph/Chl, precipitated, treated with RNase A, and electrophoresed as in Fig. 2A-C.

Lanes 1, 2, 5.89 mM NaCl; lanes 3, 4, 150 mM NaCl; Lanes 5, 6, 500 mM NaCl. Representative examples of the DNA samples extracted in the presence of SDS only (lanes 1, 3, 5) or in the presence of both SDS and EDTA (lanes 2, 4, 6) are shown.

(H) DNA from cells fixed with 2% FA and inactivated with 0.125 M glycine cannot be isolated using column kit as well as extracted with Ph/Chl after storing at −20 °C. Cells were harvested, washed with 1X PBS, fixed with 2% FA in 1X PBS at RT of 10 min, quenched with 0.125 mM glycine for 5 min and washed twice with 1X ice-cold PBS and were placed at −20 °C O/N. After that DNA was isolated on column of GeneJET Genomic DNA purification kit, according to the manufacturer's protocol and processed w/o or with RNase A (lanes 1,2, respectively). Alternatively, EB was added and the cells were incubated to reverse cross-links as in Fig. 2A-C. Then DNA was extracted with Ph/Chl, precipitated, dissolved, processed w/o or with RNase A (lanes 3,4, respectively) and subjected to electrophoresis as in Fig. 2A-C.

(I) DNA from cells fixed with 2% FA and inactivated with 0.125 M glycine after storing at −20 °C cannot be isolated using a protocol for formalin-fixed tissues. Cells were treated as in (H) and were placed at −20 °C for a couple of weeks and DNA was isolated as follows: lane 1 – as for lane 2 in (H) (control); lane 2 – cells were processed with 1% SDS only and w/o heating according to a protocol by (Campos and Gilbert, 2012) and DNA was extracted with Ph/Chl, precipitated, dissolved, processed w/o RNase A and subjected to electrophoresis as in (H); lane 3 – as for lane 2 except that cells were additionally heated at 100 °C for 40 min; lane 4 – cells were processed with 1% SDS and 0.1N NaOH w/o heating as in lane 2; lane 5 – as in lane 4 except that cells were additionally heated at 100 °C for 40 min.

High NaCl concentrations promote the extraction of chromatin-associated proteins, including histones (von Holt et al., 1989; Shechter et al., 2007). This might be important upon reversion of cross-links and protein digestion with PrK. Therefore, we investigated the effect of the ionic strength and total composition of EB on the extraction of reversed cross-linked DNA. We compared the extraction of reversed cross-linked DNA under hypotonic (5.89 mM of NaCl), isotonic (150 mM NaCl), and hypertonic (500 mM NaCl) conditions in the presence of (i) SDS, (ii) EDTA, (iii) both EDTA and SDS (Fig. S4G), and (iv) without either agent (Table S5).

Unexpectedly, we found that some of the samples were not extracted under hypertonic conditions in the presence of SDS alone or both SDS and EDTA (Table S5). Extraction was lacking possibly because DNA solubility decreased and DNA accumulated in the interphase upon Ph/Chl extraction as a result of the so-called thickening effect of surfactant (SDS) solutions in the presence of an electrolyte (an increase in viscosity due to an increased size of micelles) (O'Lenick, 2018). Alternatively, DNA precipitation might occur in 1% SDS in the presence of 0.5 M NaCl (~2.9%) (Marko and Butler, 1951). In contrast, DNA was extracted stably in the absence of SDS, or in the absence of both SDS and EDTA, or in the presence of EDTA alone. Therefore, we do not recommend that reversion of cross-links be performed in hypertonic conditions in the presence of SDS because of the instability of the extraction process. Some of the samples were similarly not extracted in hypotonic conditions in the absence of both SDS and EDTA. However, the addition of SDS, EDTA, or both agents made it possible to successfully extract DNA of all samples. In isotonic conditions, the presence of SDS, or EDTA, or both agents did not affect DNA extraction and DNA was successfully isolated from all samples.

Table S5. The effects of SDS, EDTA, and ionic strength on the success of 3C library isolation

EB components	Type of the extraction buffer (EB)											
	1 (3/4)*	2 (4/4)	3 (4/4)	4 (4/4)	5 (4/4)	6 (4/4)	7 (4/4)	8 (4/4)	9 (4/4)	10 (4/4)	11 (3/4)	12 (3/4)
PrK	+	+	+	+	+	+	+	+	+	+	+	+
NaCl, 5.89 mM	+	+					+	+				
NaCl, 150 mM			+	+					+	+		
NaCl, 500 mM					+	+					+	+
SDS, 1%							+	+	+	+	+	+
EDTA, 30 mM		+		+		+		+		+		+

* the number of successful extractions in the total number of extractions

Thus, reversion of cross-links is successful in isotonic conditions without SDS and/or EDTA and isotonic conditions are recommended to use when a separate EB is added to samples, for example, in the case of extracting DNA from homogenized tissues. When reversion of cross-links occurs in hypotonic conditions of 1X T4 DNA ligase buffer in the 3C procedure, the addition of EDTA and/or SDS to EB is highly desirable for stable DNA extraction.

Altogether, these results suggest that the conditions of cell lysis and the step of washing nuclei at the initial stage of the protocol have the strongest effect on DNA preservation at subsequent stages. The EB composition (EDTA, SDS, and NaCl concentrations) appear to be important for preserving DNA integrity during reversion of cross-links and ensuring successful DNA extraction with Ph/Chl (see Discussion). The addition of EDTA to EB is required to protect DNA integrity when cell lysis is performed in isotonic conditions. Dilution of nuclei with a large volume of 1X T4 DNA ligase buffer for DNA ligation may provide an additional means to maintain DNA integrity if lysis was isotonic. SDS or EDTA is necessary to use during hypotonic cross-link reversal for more efficient DNA extraction, while their addition is optional in isotonic conditions. The reversal of cross-links can be performed at both standard 65 °C and a lower temperature (50 or 56 °C).

1.6 Treatment of the 3C library with RNases

Purification of a 3C library from RNA impurities might be an important step in preparation of the 3C library for analysis. Some guides indicate that very small amounts of RNA are allowed in the 3C library (van Berkum and Dekker, 2009) because PCR might be inhibited otherwise. We did not observe such inhibition in our experiments probably because RNA is unstable in an alkaline PCR buffer upon heating and is rapidly degraded in the PCR mixture. However, the inhibition cannot be completely ruled out since there are data in the literature that PCR is inhibited by residual RNA present in the sample (Yuen et al., 2001). In addition, RNA will give a false idea of the DNA amount in the 3C library if the DNA amount is estimated spectrophotometrically.

We found that RNase A from bovine pancreas (bovine RNase A) (Thermo, #EN0531), which is the most common RNase preparation, might cause DNA degradation at 37 °C regardless of whether DNA was dissolved in water or in a Tris-HCl buffer. DNA was also degraded when bovine RNase was added to the saved upper aqueous phase after sample extraction with Ph/Chl. The finding prompted us to investigate the properties of other RNases.

First, we tested RNase T1 from the mold fungus *Aspergillus oryzae,* which is another common RNase. Genomic DNA preparations contained some amount of RNA to monitor RNase activity (Fig. S5A). We observed that RNase T1 activity is not enough to completely remove rRNA bands in comparison with bovine RNase A (Fig. S5B). We concluded that RNase T1 is much inferior to bovine RNase A.

Next, we compared several available RNases in one experiment (Table S6). To eliminate residual DNase activity, the RNase preparations in 10 mM Tris-HCl were heated at 99 °C for 10 min. Activity at 37 °C was compared between the preparations tested before and after heat treatment (HT). We found that all RNases digested RNA at 37 °C both before and after HT (Fig. S5C). Before HT, RNase If (a recombinant fusion of RNase I from *E. coli* and the maltose-binding protein) showed the lowest RNase activity among all RNases. After HT, the same amount of RNase If unexpectedly digested RNA much more efficiently (ribosomal bands became undetectable) (Fig. S5C, lane 5). RNase T1 showed poor results both before and after HT (ribosomal bands were observed in both cases) (Fig. S5C, lanes 2,3). RNase I and recombinant RNase A (rRNase A) demonstrated the highest RNase activity. Either enzyme efficiently removed ribosomal RNA before and after HT (Fig. S5C, lanes 4,6). Bovine RNase A tested before HT degraded DNA at 37 °C within 30 min (Fig. S5C, Lane 1), completely in line with preliminary observations. However, HT eliminated this DNase activity.

We performed the same experiment, but heated aliquots of RNase preparations in their own storage buffers at 99 °C for 10 min. Then we checked their activities at RT for 30 min without and with HT and compared the results obtained at RT and 37 °C. It was found that all RNases digested RNA at RT both before and after HT (Fig. S5D). RNase T1 activity was poor and did not change after HT (Fig. S5D, Lanes 2, 3). RNase If tested before HT unexpectedly showed greater activity at RT than at 37 °C (compare Fig. S5 C and D, Lanes 5). After HT, RNase If activity at RT was slightly reduced (ribosomal bands remained detectable) (Fig. S5C, Lane 5). RNase I and rRNase A showed consistently high activity both before and after HT (Fig. S5C, Lanes 4, 6). Bovine RNase A began to degrade genomic DNA after HT, but there were no traces of DNA degradation without HT (Fig. S5D, Lane 1).

Table S6. Comparison of different RNases

	Activity towards RNA				Activity towards DNA			
	37 °C		RT		37 °C		RT	
RNase	w/o HT	with HT	w/o HT	with HT	w/o HT	with HT	w/o HT	with HT
RNase A bovine	+++	+++	+++	+++	++	-	-	++
RNase T1	++	++	++	++	-	-	-	-
RNase I	+++	+++	+++	+++	-	-	-	-
RNase If	+	+++	+++	++	-	-	-	-
rRNase A	+++	+++	+++	+++	-	-	-	-

We concluded that RNase T1 is not recommended to use for removing RNA from genomic DNA preparations because of its low activity at both 37 ° C and RT; heating did not influence its activity. RNase If may be used without prior heating to digest samples at RT instead of 37 °C for its maximum activity. Heating affected the activity of RNase If in different ways: when heated in Tris-HCl, its activity increased; when heated in its own storage buffer, its activity slightly decreased. RNase I and rRNase A proved to be the best both in terms of activity towards RNA and safety for DNA. They produce stable results at both 37 °C and RT and can be used without prior heating.

Bovine RNase A demonstrated a variety of results. Highly efficient towards RNA as it is, the enzyme degraded genomic DNA at 37 °C as well, while this regimen is standardly used to remove RNA from DNA preparations. Preliminary heating in a Tris buffer was required to eliminate this residual DNase activity and to make the enzyme safe for DNA at 37 °C. Surprisingly, there was no DNA degradation by bovine RNase A at RT, but DNA degradation became detectable after heating the enzyme in its own buffer. Thus, heating in its own buffer stimulated DNase activity of bovine RNase A.

Since bovine RNase A is most commonly used in laboratories to remove RNA impurities and some 3C methodical articles state directly that contaminating RNA should be degraded with RNase A treatment at 37 °C (Belton et al., 2012), we examined in more detail the conditions in which bovine RNase A is efficient without being harmful for DNA integrity. We added new conditions: DNA treatment in 0.5X bovine RNase A storage buffer (1X: 50 mM Tris-HCl, pH 7.4, 50% glycerol) at RT and at 37 °C before and after HT of the enzyme preparation at 99 °C for 10 min. We assumed that the reaction could proceed differently in a viscous medium created by 25% glycerol. A few

repetitions of the above experiments were conducted as well to make sure that our results are correct. The results of all experiments are summarized in Table S7. An analysis of Table S6 showed that treatment at RT without prior inactivation of residual DNase activity by heating is the most convenient and the only stable regimen to use with bovine RNase A.

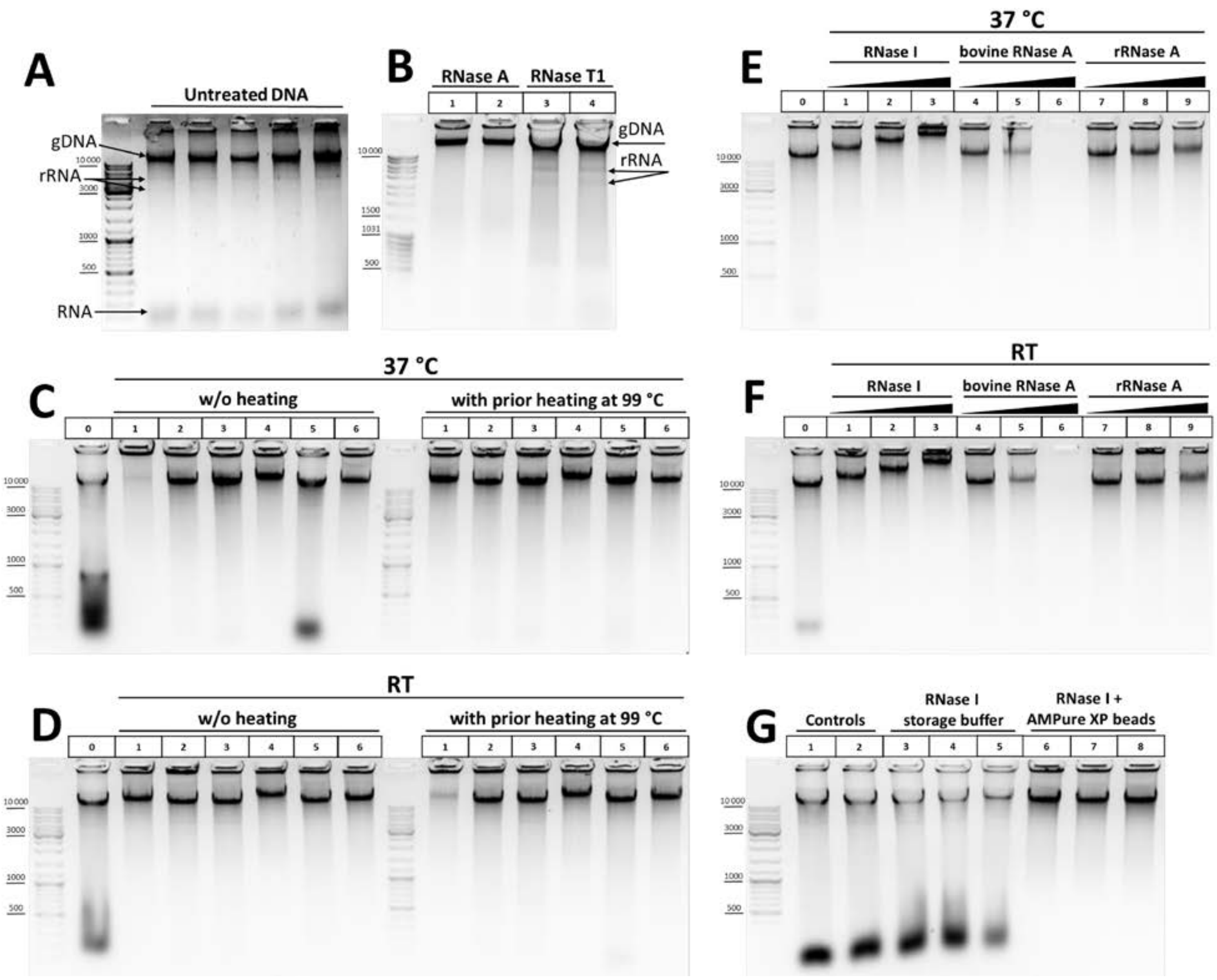

Figure S5. Purification of the 3C library from RNA impurities with RNases.

(A) Genomic DNA was extracted and dissolved as in Fig. 5E in five replicates except that DNA was not treated with RNases and not purified on AMPure XP beads to preserve low-molecular-weight RNA. A 0.2-µl aliquot of a DNA preparation was used for electrophoresis.

(B) Genomic DNA (1 µl) from (A) was treated with 5 µg of bovine RNase A (Thermo, #EN0531, conc. 10 mg/ml) at RT for 30 min (lanes 1, 2) or 500 U of RNase T1 (Thermo, #EN0542, conc. 1000 U/µL) at 37 °C for 30 min (lanes 3, 4) in mQ water (lanes 1, 3) or 10 mM Tris-HCl (pH 8.0) (lanes 2, 4). One of the three replicate experiments is shown.

(C) Aliquots of commercially available RNase preparations were preheated in 10 mM Tris-HCl (pH 8.0) at 99 °C for 10 min or used without preheating (as indicated at the top) and then were mixed with 1 µl of genomic DNA in a volume of 10 µl and incubated in 10 mM Tris-HCl (pH 8.0) at 37 °C

for 30 min. The amount of an RNase preparation in the reaction was 1 µl. Lane 0, genomic DNA untreated with RNase (control); lane 1, bovine RNase A (Thermo, # EN0531, conc. 10 mg/ml); lane 2, RNase T1 (Thermo, #EN0541, conc. 1000 U/µL); lane 3, RNase T1 different lot (Thermo, #EN0542, conc. 1000 U / µL); lane 4 - RNase I (Thermo, #N0601, conc. 10 U/µL); lane 5, RNase If (NEB, #M0243S, conc. 50,000 U/ml); lane 6, recombinant RNase A (Thermo, #AM2269, conc. 1 mg/ml). One of the three replicate experiments is shown.

(D) The experiment was done as in (C) except that aliquots of RNases were preheated in their own storage buffers and were incubated with the genomic DNA at RT. Designations of the lanes are as in (C).

(E) The experiment was done as in (C) except that RNases were not preheated. Genomic DNA was treated with 0.5 µl (Lanes 1, 4, 7), 2.5 µl (Lanes 2, 5, 8), and 5 µl (Lanes 3, 6, 9) of the enzymes as indicated at the top. Designations of the lanes and concentrations of the enzymes are as in (C).

(F) The experiment was done as in (E) except that reactions were carried out at RT.

(G) Genomic DNA was incubated in a reconstructed RNase I storage buffer (lanes 3-5) or treated with increasing concentrations of RNase I as in (E) and then purified on AMPure XP magnetic beads. Lane 1, DNA in 1X TAE buffer (control #1); lane 2, DNA in 50 mM Tris-HCl (pH 8.0) (control #2); lanes 3-5, DNA mixed with 0.5, 2.5, and 5 µl of RNase I storage buffer (50 mM Tris-HCl, pH 8.0, 100 mM NaCl, 0.01% Triton X-100, 50% glycerol), respectively; lanes 6-8, DNA treated with 0.5, 2.5, and 5 µl of RNase I and then purified on AMPure XP magnetic beads. One of the three replicate experiments is shown.

Table S7. The effect of bovine RNase A on the genomic DNA integrity

Conditions	Experiment 1	Experiment 2	Experiment 3	Conclusion
w/o heating, reaction in 10 mM Tris-HCl at 37 °C	+	–	–	Unstable result
w/o heating, reaction in 10 mM Tris-HCl at RT	–	–	–	–
w/o heating, reaction in 0.5X bovine RNase A storage buffer at 37 °C (new condition)	+	–	–	Unstable result
w/o heating, reaction in 0.5X bovine RNase A storage buffer at RT (new condition)	Not determined	–	–	–

with heating in 10 mM Tris-HCl, reaction in 10 mM Tris-HCl at 37 °C	–	+	+	+
with heating in 10 mM Tris-HCl, reaction in 10 mM Tris-HCl at RT	Not determined	+	+	+
with heating in 1X bovine RNase A storage buffer, reaction in 0.5X bovine RNase A storage buffer at RT	Not determined	–	–	–
with heating in 1X bovine RNase A storage buffer, reaction in 10 mM Tris-HCl at RT	+	Not determined	+	+
with heating in 1X bovine RNase A storage buffer, reaction in 0.5X bovine RNase A storage buffer at 37 °C	–	–	–	–
with heating in 1X bovine RNase A storage buffer, reaction in 0.5X bovine RNase A storage buffer at RT	Not determined	–	–	–

(+), DNA degradation; (–), no DNA degradation.

Next, we studied in more detail the properties of rRNase A and RNase I as the enzymes that are most active and safest for genomic DNA and compared their properties with the properties of bovine RNase A at 37 °C and RT. It was found that increasing concentrations of bovine RNase A and, to a lesser extent, rRNase A caused DNA degradation (Fig. S5E). This effect was more pronounced with bovine RNase A than with rRNase A and was independent of the incubation temperature (Fig. S5E,F). The findings indicate that DNA integrity depends more on the enzyme concentration in the reaction, but not on the reaction temperature, in the case of treatment with bovine RNaseA and, to a lesser extent, rRNase A (Fig. S5C-F). At higher enzyme concentrations, bovine RNase A degraded DNA even at RT (Fig. S5F).

Importantly, there was no such an effect in the case of RNase I. Conversely, insufficient addition of RNase I (up to 0.4 U/µl) resulted in residual RNA remaining in the sample, while an increase in RNase I concentration up to 1 U/µl led to efficient removal of all RNA (not shown). However, an increase in RNase I concentration was accompanied by an increase in DNA retardation in gel. This may be indicative of RNase I binding to genomic DNA. The RNase I storage buffer contained 100 mM NaCl, which might interact with DNA to reduce its mobility in gel. To exclude the effect of the ionic strength, DNA mobility was studied in the presence of the same amount of the RNase I storage buffer. The storage buffer was found to exert a minor, if any, effect on the DNA mobility, although the mobility of low-molecular-weight RNA changed with the increasing amount of the storage buffer (Fig. S5G, lanes 1-5). In addition, we checked whether RNase I binding to DNA is possible to prevent by purifying DNA on magnetic beads. Indeed, DNA retardation in gel became undetectable

after purification on magnetic beads (Fig. S5G, lanes 6-8). We concluded that RNase I binding to genomic DNA was responsible for significant DNA retardation in gel. DNA degradation after RNase I treatment was not observed in DNA samples stored on ice, at –20 °C, or at –70 °C for at least one month (not shown).

Taken together, these data suggest that RNase I is the enzyme of choice for efficient removal of RNA impurities from 3C library preparations, Treatment with RNase I can be carried out at RT or 37 °C. A fallback option is to use rRNase A at a low concentration at 37 °C or RT. Bovine RNase A is not recommended, but is still possible to use at low concentrations at RT.

1.7 Purification of the 3C library on magnetic beads

An additional purification step may be required and highly desirable to perform prior to finally analyze the 3C library for several reasons. First, we observed in preliminary experiments that, when EB containing of 30 mM EDTA is used to reverse cross-links after extraction with Ph/Chl and ethanol precipitation, a considerable amount of EDTA remains in the precipitate even after multiple washings with 70% ethanol. This EDTA amount totally inhibits PCR after dissolving the DNA pellet. Thus, it is necessary to purify DNA from impurities once again after Ph/Chl extraction. Second, we found that bovine RNase A degrades DNA when added to the saved upper aqueous phase after extraction with Ph/Chl. Therefore, if it is necessary to treat the 3C library with bovine RNase A, this treatment should be carried out after Ph/Chl extraction and DNA precipitation with ethanol and DNA should be purified once again to remove the traces of bovine RNase A.

Solid-phase reversible immobilization (SPRI) technology based on paramagnetic Beckman Coulter AMPure XP beads, Illumina Sample Purification Beads (SPB), and Nimagen AmpliClean beads can be used for the purpose. These beads are routinely used to make size-selection and to purify PCR products and Hi-C libraries (Rao et al., 2014; Belaghzal et al., 2017; Canela et al., 2017).

Before starting experiments with AMPure XP beads, we worked with Nimagen AmpliClean beads. Our work was unsuccessful since AmpliClean beads isolated DNA for about 1 month from the moment a new package was opened, and absorption/elution became inefficient afterwards. Starting to use AMPure XP beads, we found that a ligation pattern observed when a 3C library was purified on beads (Fig. S4A) differed from that observed with ligation samples purified by other means (Fig. 6E). Since the restriction control gave the expected DNA pattern after RE inactivation (Fig. S4A Lane 2), we assumed that DNA degradation might occur at some stages after RE inactivation, e.g., upon T4 DNA ligase inactivation at 65 °C for 10 min. Alternatively, DNA ligation after purification on beads might be inhibited to an extent. We therefore studied the DNA pattern at all stages after RE inactivation and DNA purification on beads. Standard ethanol precipitation was used as an alternative to purification on beads. It was found that the pattern changed immediately after elution from beads (Fig. S4B) or after ethanol precipitation (Fig. S4C) and remained unchanged at subsequent steps despite T4 DNA ligase activity. We hypothesized that DNA could be degraded when heated in water at 55 ° C for 15 min upon elution from beads and, the same way, when heated in water at 75 ° C for 5 min upon dissolution after ethanol precipitation.

In order to assess whether DNA hydrolysis occurs upon heating at 55 °C for 15 min in our conditions, we compared DNA integrity for DNA samples dissolved in pure water, NaCl solutions,

and Tris-HCl buffers of different concentrations (Fig. S4D). DNA degradation was found occur only upon heating in water.

Thus, heating of DNA samples in pure water upon elution from AMPure XP beads results in DNA degradation and degraded DNA is no longer ligated properly with T4 DNA ligase, probably because non-cohesive ends form via hydrolysis in water.

Next, we investigated the conditions in which DNA can safely be eluted from beads. We observed that digested DNA degraded upon elution from beads in water at 55 °C was not successfully ligated at subsequent steps (Fig. S4E). However, elution with 10 and 25 mM NaCl or 10 and 50 mM Tris-HCl prevented DNA degradation upon heating at 55 °C (Fig. S4E). When the elution temperature and elution duration were decreased to RT and 5 min, respectively, DNA remained intact even in water (Fig. S4F).

We concluded that elution from beads with water at elevated temperatures, which has been recommended in some protocols to enhance the elution of high-molecular-weight DNA fragments and to improve the recovery, provokes DNA degradation in our conditions. Heat-mediated hydrolysis of DNA in pure water was observed in our and other studies (Fattorini et al., 2018). Since water is released upon the formation of phosphodiester bonds, equilibrium of the reaction is shifted towards decomposition of RNA or DNA polymers in an aqueous solution. Obviously, heating, which increases the movement of molecules, will only spur this process. Hence, when eluting a 3C library from beads, it is better to avoid heating the beads in water to preserve DNA integrity. If water is necessary to use for elution, then elution should be done at RT. Alternatively, DNA can be eluted with a Tris buffer or a NaCl solution at an elevated temperature to hasten the process or to increase the DNA yield. It is especially convenient to elute with NaCl since the resulting DNA, e.g., plasmid DNA, oligo, or dsRNA, can be directly added to cells or taken for microinjection in this case, avoiding Tris cytotoxicity.

We determined additionally whether AMPure XP beads isolate cross-linked DNA. It was found that both cross-linked DNA and DNA obtained after reversion of cross-links were successfully isolated on the beads (Fig. S4G). The beads are similar in this property to Ph/Chl extraction and compare favorably with a column DNA isolation kit, which did not isolate cross-linked DNA (Fig. S2A-D). The method and can be used in combination with Ph/Chl extraction when isolation of cross-linked DNA is required.

Further, we compared purification on magnetic beads with a column-based purification approach. It was found that a Zymo Research DNA purification column system is inferior to AMPure XP magnetic beads; the beads showed better isolation of high-molecular-weight DNA fragments after DNA ligation (>10-12 kb) (Fig. S4H). Moreover, DNA restriction and ligation samples purified on the beads were more similar to samples examined before isolation (Fig. 5E) than Zymo kit-purified samples (Fig. S4H). We concluded that purification on magnetic beads is superior to the column-based approach.

Finally, we checked whether it is possible to keep DNA on AMPure XP beads overnight upon DNA isolation and examined how long DNA can be kept on ice between steps of the 3C procedure. We found no significant traces of DNA degradation, provided that DNA was stored on beads under 75% ethanol at –20 °C or RT overnight (Fig. S4I, Lanes 1,3,5, compare with Fig. 6E). We observed additionally that there was no DNA degradation when genomic DNA was kept on ice for at least two weeks (Fig. S4I, Lanes 2, 4, 6). We concluded that DNA samples can be stored on ice without

freezing –20 °C between the steps of DNA elution from beads and an analysis of the 3C library. In addition, at least short-term (overnight) storage of DNA on beads is also possible at any temperature before elution.

Thus, AMPure XP magnetic beads can be efficiently used for additional purification of 3C libraries in combination with elution from the beads with a Tris buffer or at least 10 mM NaCl. Purification on beads is superior to column-based purification, and DNA purified on beads can be stored on ice until further analysis for at least several weeks.

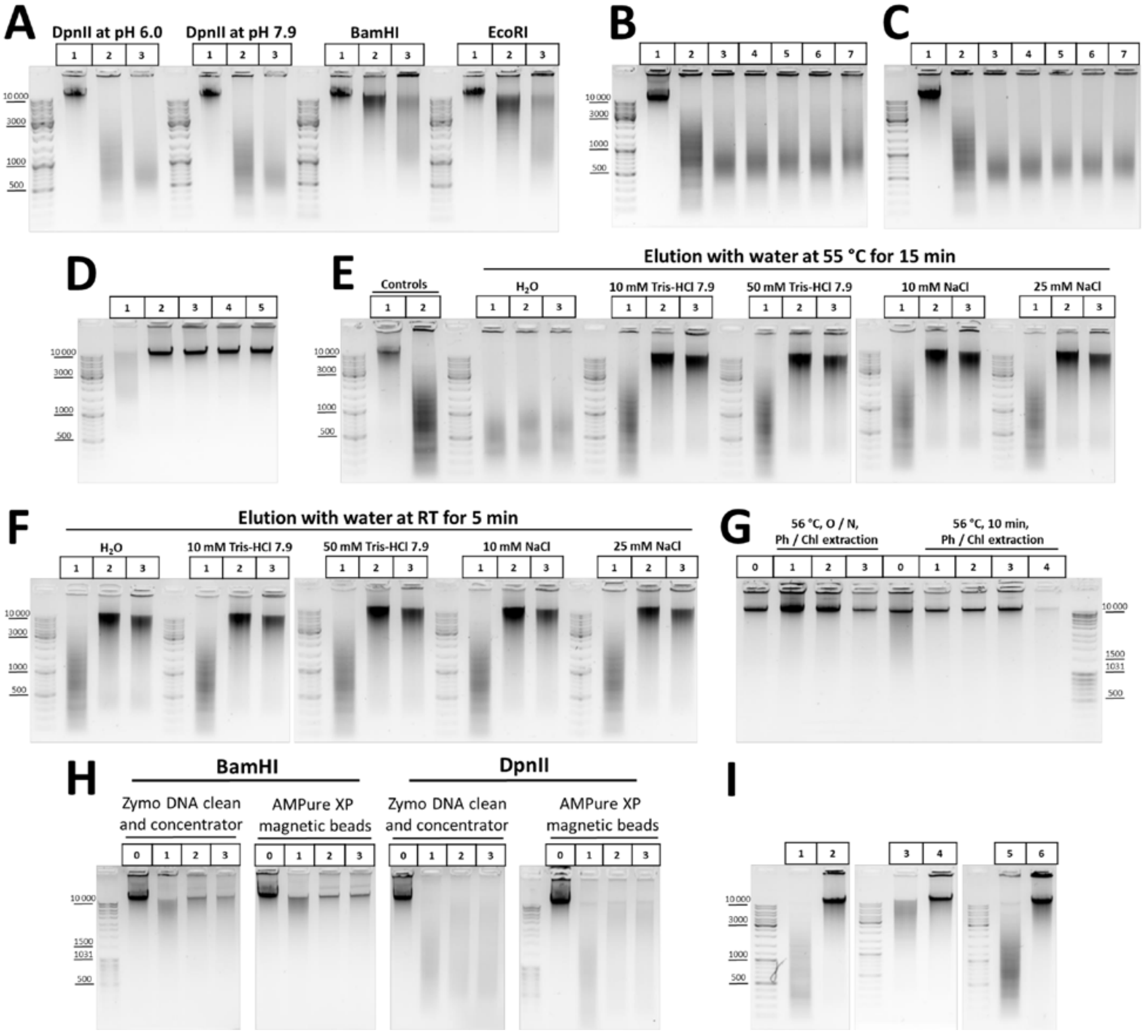

Figure S6. (A) Patterns of genomic DNA digested with 4-bp and 6-bp cutters and ligated in solution after its purification on AMPure XP magnetic beads. Genomic DNA was isolated, treated with RNase, precipitated with ethanol, and dissolved as in Fig. 5E. The experiment was done as in Fig. 5E

except that the beads were incubated in mQ water at 55 °C for 15 min to elute DNA after DNA ligation. Lane 1, non-digested DNA; lane 2, digested DNA; lane 3, DNA ligated in solution after purification on AMPure XP beads. One of the three replicate experiments is shown.

(B, C) Elution or dissolution of DNA by heating in water affects the DNA pattern. (B) Genomic DNA was prepared as in (A). The experiment was done as in Fig. 5E except that DNA was purified on AMPure XP beads after heat inactivation of RE. Lane 1, non-digested DNA; lane 2, digested DNA after heat inactivation of RE at 65 °C for 20 min; lane 3, digested DNA was purified on beads (elution with mQ water at 55 °C for 15 min); lane 4, 10X T4 DNA ligase buffer was added to digested DNA and beads-purified DNA to a 1X final concentration, and the samples were incubated in 1X ligase buffer at 22 °C for 1 h; lane 5, the same as in lane 4, but DNA was incubated at 65 °C for 10 min (imitation of T4 DNA ligase inactivation) after incubation in 1X ligation buffer; lane 6, the same as in lane 4, but DNA was incubated in 1X ligation buffer in the presence of 5 U of T4 DNA ligase and 1 mM ATP; lane 7, the same as in lane 6, but the sample was incubated at 65 °C for 10 min after DNA ligation to inactivate T4 DNA ligase. (C) The same as in (B), but, instead of using AMPure XP beads to purify DNA after heat inactivation of RE, DNA was precipitated and washed with ethanol as in Fig. 2A-C and then incubated in mQ water at 75 °C for 5 min to completely dissolve the dried DNA pellet (lane 3). Then 10X T4 DNA ligase buffer was added to DNA digested and precipitated with ethanol to make a 1X final concentration, and DNA was incubated in 1X ligase buffer at 22 °C for 1 h (lane 4). Designations of the other lanes are as in (B). One of the three replicate experiments is shown.

(D) Integrity of genomic DNA in various buffers. Genomic DNA was prepared as in (A). Then DNA was dissolved as follows: lane 1, in water (water was filtered through a 0.22-µm filter and autoclaved); lane 2, in 10 mM Tris-HCl (pH 7.9) (made with water as in lane 1 and filtered through a 0.22-µm filter after adjusting pH); lane 3, in 50 mM Tris-HCl (pH 7.9) (treated the same way as in lane 2); lane 4, in 10 mM NaCl (treated the same way as in lane 1); lane 5, in 25 mM NaCl (treated the same way as in lane 1). After dissolution, all samples were heated at 55 °C for 15 min, and DNA integrity was checked by electrophoresis. One of the three replicate experiments is shown.

(E, F) Conditions of safe DNA elution from AMPure XP beads. (E) Genomic DNA was prepared as in (A) (lane 1, controls). The experiment was done as in Fig. 5E. After digestion, RE was heat-inactivated at 65 °C for 20 min and DNA was electrophoresed (lane 2, controls) or purified on AMPure XP beads (lane 1). Elution from the beads was done at 55 °C for 15 min; the buffers are specified at the top. Then 10X ligation buffer was added to the samples to a 1X final concentration. DNA was incubated in the presence of 5 U of T4 DNA ligase and 1 mM ATP at 22 °C for 1 h (lane 2) or treated as in lane 2 and then incubated at 65 °C min for 10 min to inactivate T4 DNA ligase (lane 3). (F) The same as (E), but elution from the beads was done with water at RT for 5 min. One of the three replicate experiments is shown.

(G) Isolation of DNA on AMPure XP beads after cross-linking and reversion of cross-links. The experiment was done as in Fig. S4A-D. Cells were fixed with different PFA concentrations or left unfixed. After adding EB, cells were incubated as indicated at the top. DNA was purified on beads after treatment with bovine RNase A. Elution from the beads was done with water at RT for 5 min. Lane 0, DNA from unfixed cells (control); lane 1, 0.5% PFA; lane 2, 1% PFA; lane 3, 1.5% PFA; lane 4, 2% PFA. One of the three replicate experiments is shown.

(H) Comparison of DNA purification on magnetic beads and on a column. Genomic DNA was prepared as in (A). The experiment was done as in Fig. 5E except that DNA was purified using either

a Zymo DNA Clean & Concentrator-25 kit (# D4033) or AMPure XP beads after restriction digestion and then electrophoresed. Alternatively, digested DNA was precipitated and washed with ethanol as in Fig. 2A-C, dissolved in 10 µl of 10 mM Tris-HCl, ligated in the presence of different ATP concentrations, and purified using a Zymo kit or AMPure XP beads as indicated at the top. Elution from the beads was done with water at RT for 5 min. Lane 1, non-digested DNA; lane 2, digested DNA; lane 3, DNA ligated in the presence of 1 mM ATP; lane 4, DNA ligated in the presence of 5.5 mM ATP. One of the three replicate experiments is shown.

(I) DNA storage on AMPure XP beads and on ice. Lane 1, DpnII-digested DNA was kept on beads under 75% ethanol at RT overnight before elution from the beads; lane 2, uncut DNA was kept on ice for two days; lane 3, the same as in lane 1, but DNA was digested with EcoRI; lane 4, the same as in lane 2, but DNA was kept on ice for a week; lane 5, the same as in lane 1, but DNA was kept under 75% ethanol at −20 °C overnight; lane 6, uncut DNA was kept on ice for two weeks.

2 Protocol of 3C library preparation from Drosophila S2 cells (Sg4 clone)

This protocol is suitable for 12 million (approximately 10 mg) *Drosophila* S2 cells. This amount of cells is required for one replicate. Several replicates are generally recommended to perform. Count the cells before beginning the procedure. This protocol suggests the use of DpnII and one fixing agent (formaldehyde, FA).

I. Cell fixation, FA inactivation, and storage of nuclei

1. Transfer the cells from a cell culture flask to a 50-ml tube. Centrifuge at 1 kg for 5 min. Remove the medium.

2. Resuspend the pellet in 1 ml of 1X PBS, and transfer the suspension to a 1.5-ml tube. Centrifuge at 2.5 kg for 1 min (or 5 kg for 30 sec). Discard the supernatant.

3. Weigh the cell pellet on an accurate balance.

4. Resuspend the pellet in 1 ml of 1X PBS.

5. One experimental replicate requires 10 mg of cells. According to the number of replicates processed, calculate the corresponding volume of the cell suspension, and transfer it to a new tube (1.5-ml for 1 replicate, 15- or 50-ml tube for several ones).

6. Centrifuge the suspension at 2.5 kg for 1 min. Discard the supernatant.

7. Resuspend the pellet in 0.5 ml of 1X PBS (per 10 mg of cells). Increase the volume proportionally to the number of replicates.

8. Filter the suspension through a 40-µm Cell Strainer Nylon (Corning Falcon, cat. no. 352340) two or three times to remove cell aggregates. Do not centrifuge after filtration. Adjust to the initial volume with 1X PBS.

9. Add the equal volume of 1% FA in 1X PBS (for example, for 5 pooled replicates, mix 2.5 ml of 1% FA in 1X PBS with 2.5 ml of 1X PBS containing 50 mg of cells). Incubate at RT for 10 min (rotate at 30 rpm).

The final FA concentration is usually 0.5-1%. Note that a 1% FA solution prepared from PFA powder provides a higher fixation strength than a similar solution prepared from a 37% ready-to-use commercial solution. 0.5% FA made from PFA and 1% FA made from a 37% commercial solution show comparable fixation efficiencies.

10. Add 166.5 µl of 2 M glycine per 1 ml of 0.5% FA to quench the reaction (0.333 M glycine final; keep this Gly/FA proportion with other FA concentrations), mix thoroughly, keep at RT for 5 min (rotate at 30 rpm). Glycine is possible to add in a slight excess (see Table S1), but not more than 200 µl per 1 ml of 0.5% FA (400 mM glycine final).

11. Add equal volume of ice-cold 1X PBS. This helps to obtain a more compact pellet after centrifugation. Centrifuge at 2.5 kg at 4°C for 3 min (or 5 kg at 4°C for 1 min). Remove the supernatant.

12. Wash the cells one or two times with an equal volume of ice-cold 1X PBS. Centrifuge at 2.5 kg for 2 min (or 5 kg for 1 min) between washings. The cell pellet can be resuspended by slight vortexing between washings. Remove the supernatant completely.

Optional stopping point. Fixed cells could be flash frozen in liquid nitrogen at this stage and stored at −80°C. However, we do not recommend storing fixed material at this point. Proceed to cell lysis immediately.

13. Split the replicates at this step (resuspend the pellet in ice-cold 1X PBS, split to several tubes, centrifuge as in the previous step, and discard the supernatant).

II. Cell lysis

14. Resuspend the cell pellet of each replicate in 250 µl of ice-cold Lysis Buffer (50 mM Tris–HCl pH 8.0, 10 mM NaCl, 0.5% NP-40, 1% Triton X-100, protease inhibitors), incubate for 10-15 min in ice.

15. Centrifuge at 5 kg at 4°C for 1 min, carefully remove the supernatant completely.

16. Wash the pellet with 250-500 µl of ice-cold Lysis Buffer once. Centrifuge at 5 kg at 4°C for 1 min, carefully remove the supernatant completely.

Optional stopping point. At this stage the nuclei can be left in the washing buffer to stay overnight in ice (this does not lead to DNA degradation). If this option is chosen, sediment the nuclei at 7.5 kg after overnight incubation on ice and wash once with 250-500 µl of ice-cold Lysis Buffer once again.

The nuclear pellet could be flash frozen now in liquid nitrogen and stored at −80°C, if required. However, we recommend proceeding to the next step immediately.

III. Nucleoplasm release and chromatin treatment with heat

17. Resuspend the nuclear pellet in 250 µl of 0.1-0.3% SDS in water. Incubate at 65°C for 5 min (alternatively, incubate at 37°C for 10 min-1 h) (shake at 900 rpm to prevent sedimentation of the nuclei).

18. Add 25 µl of 20% Triton-X100 (1.8% final). Incubate for 15 min at 37°C (alternatively, incubate at 37°C for 1 h) (shake at 900 rpm).

19. Centrifuge at 5 kg at 4°C for 1 min.

20. Wash the nuclei three times with 100-200 µl of ice-cold 1X restriction buffer (RB) for DpnII. Centrifuge at no more than 400 g at 4°C for 1 min between washings. Resuspend the pellet by pipetting or vortexing between washings. During the final washing, resuspend the nuclei in 100-200 µl of 1X RB, centrifuge at low speed, and remove the supernatant so that 50 µl of the supernatant remains in the tube with the nuclei. Gently resuspend the nuclei by pipetting if necessarily.

Optional stopping point. At this stage the nuclei can be left in 1X RB to stay overnight in ice (this does not lead to DNA degradation).

21. Take 1/10 of the volume as a Chromatin Integrity Control (Control #1). Add to the control sample 215 µl of 1X T4 DNA Ligase Buffer (buffer without ATP is recommended) and keep the control on ice or freeze at −20°C. However, we recommend processing the control immediately and proceed to step 28.

IV. Digestion of DNA in nuclei

22. Add 2 µl of DpnII (100 U) (NEB). Incubate at 37°C for at least 3 h (overnight incubation is recommended) (shake at 1,100 rpm to prevent sedimentation of the nuclei).

23. Centrifuge at 7.5 kg at 4°C for 1 min.

24. Wash the pellet three times with 100 µl of 1X T4 DNA Ligase Buffer (a buffer without ATP is recommended). Centrifuge at 7.5 kg at 4°C for 1 min between washings. Discard the supernatant.

V. Ligation of DNA in nuclei

25. Resuspend the nuclei in 45 µl of 1X T4 DNA Ligase Buffer. Take 1/10 of the volume as a Digestion Control (Control #2). Add to the the control sample 215 µl of 1X T4 DNA Ligase Buffer and keep the control on ice or freeze at −20 °C. However, we recommend processing the control immediately and proceed to step 28.

26. Add 1 µl (10 Weiss U) of highly concentrated T4 DNA ligase (Sibenzyme) (0.25 Weiss U/µl final concentration), 0.4 µl of 100 mM ATP (1 mM final) and 0.2 µl of 20% Triton X-100 (0.1% final) (prevents adhesion of nuclei on the walls of the tube during shaking). Incubate at 16°C or at RT overnight (shake at 1,400 rpm to prevent sedimentation of the nuclei). The T4 DNA ligase concentration at this stage should be 0.025 - 0.25 Weiss U/µl to maintain efficient ligation.

VI. Reversion of cross-links and isolation of a 3C library

27. Add to the ligation sample 179 µl of 1X T4 DNA Ligase Buffer.

28. Process the controls collected previously from this step on. Add 2.5 µl of Proteinase K (200 µg/ml final), 12.5 µl of 20% SDS (1% final), and 15 µl of 0.5M EDTA (30 mM final) to the control samples #1,2 and to the ligation sample (the use of premix of SDS, EDTA and Proteinase K in 1X T4 DNA Ligase Buffer is recommended). Incubate at 56°C overnight (or in a regimen indicated in the Table S4), cool to RT.

29. Add an equal volume of phenol/chloroform (1:1, pH 8.0), mix intensely by vortexing. Centrifuge at a maximum speed at RT for 5 min. Collect the aqueous phase and add an equal volume of chloroform. Centrifuge at a maximum speed at RT for 3 min. Collect the aqueous phase (approximately 250 µl).

30. Add 27,5 µl of 3M NaOAc and 5 µl of glycogen (100 µg/ml final). Add 750 µl of 96% EtOH, incubate at −80°C for 0.5 h. Centrifuge at a maximum speed at 4°C for 20 min. Dissolve the pellet in 20 µl (25 µl for control samples) of 10 mM Tris-HCl pH 8.0.

At this stage, routinely check the quality of the samples by electrophoresis using 1 µl of the sample after ligation and 10 µl of control samples. If the electrophoretic pattern resembles Figure 6C, proceed to the next step.

VII. Treatment of the 3C library with RNase

31. Add 1 µl (10 U) of RNase I (Thermo). Alternatively, 0.5-1 µl (0.5-1 µg) of rRNase A or 0.5 µl (5 µg) of bovine RNase A (Thermo) could be used. Incubate for 30 min at RT.

After RNase treatment you can check the quality of the samples once again by electrophoresis using 1 µl of the sample after ligation and 10 µl of control samples (optional). If the electrophoretic pattern resembles Figure 6C, proceed to the next step.

VIII. Purification of the 3C library on magnetic beads and DNA analysis

32. Purify DNA using magnetic beads (Beckman Coulter AMPure XP beads) according to the manufacturer’s protocol with the following modifications: add a 1.5X volume of a bead suspension to the 3C library sample and Chromatin Integrity Control, and a 2X volume to the Chromatin Digestion Control, wash the bead pellet three times with 1 ml of 75% EtOH with vortexing, elute with 20 µl (for 3C library sample) - 25 µl (for control samples) of 10 mM Tris-HCl pH 8.0 at RT. If magnetic beads are not available, do not use EDTA in DNA extraction buffer during reversion of cross-links because it inhibits PCR after DNA precipitation with EtOH (use SDS only).

33. Measure DNA concentration using Qubit (dsDNA HS kit).

34. Analyze the control samples and the 3C library by electrophoresis now. Load 200 ng of DNA (300-400 ng for Digestion Control) per lane. The pattern should be as in Figure 6C. The Chromatin Integrity Control lane should contain only high-MW DNA (more than 10-12 kbp). DNA of the Chromatin Digestion Control should be shorter than 3 kbp (intensity peak at 1 kbp). Ligated DNA of the 3C library should be about 10 kbp.

3 Materials and Methods

Drosophila cells

Cultured S2 *Drosophila melanogaster* embryonic cells (Sg4 clone) were kindly provided by Dr. N.E. Vorobyeva and were maintained in the SFX-Insect medium (HyClone) in the presence of penicillin/streptomycin (HyClone).

Preparation of fixing agent

4% aqueous FA solution was obtained from dry paraformaldehyde (PFA) (MP Biomedicals #150146) as follows: 0.8g of dry PFA was dissolved in a fume hood in 20 ml of water, 4 drops of 1N NaOH from a 200-µl sampler were added to obtain a solution with pH 7.0 (3 drops would give a solution with pH 6.5), and the solution was incubated at 80 °C until PFA completely dissolved (about 2 min). The solution is stored at +4 °C. Strongly diluted aqueous FA solutions are stable at + 4 °C and do not lose their activity when stored at +4 °C for at least several months. Alternatively, a 37% solution can be prepared from dry PFA as follows: 0.37 g of PFA is mixed with 1 ml of water in a 1.5-ml tube and 14 µl of 1N NaOH is added. The solution is incubated at 99 °C until PFA dissolved and stabilized at pH 7.0 (1-3 min). A fresh 37% solution is added in a necessary proportion to the buffer or medium for fixation. For stabilization at RT, 6-13% methanol can be added to prevent polymerization and precipitation of polymers. A 37% solution can be frozen at –20 °C without adding methanol. After thawing, the solution needs to be heated at 65 °C for 20 min to dissolve precipitated polymers and return the pH to 7.0.

Design of primers and probes

TaqMan probes and anchor and tester primers (Table S8) were designed against the ends of ligated fragments within 100 bp from the DpnII cutting site so that the anchor primer and probe hybridize to opposite strands of the anchor fragment (Splinter et al., 2006). The TaqMan probes were labeled at the 5' end with FAM and one T from 10 to 16 nucleotides of each probe was labeled with BHQ1.

qPCR

qPCR was performed in 4 replicates with 3C-library DNA, 0.3 µM of each primer, 0.3 µM TaqMan target probe, 1 U of Hot Start Taq DNA pol (Sibenzyme), 0.2 mM dNTP, 2 mM $MgCl_2$ in 10 µl volumes on a CFX 96 touch machine (Bio-Rad) with the following program: initial denaturation at 94°C for 1 min followed by 45 cycles of 94°C for 10 s and 60°C for 1 min. Regeneration and circularization of the DpnII site were calculated against a calibration curve, which was constructed using 10-fold dilutions of the PCR product obtained with the primers RpII_1_Forward and RpII_3_Reverse#2 (Table S8). The PCR product was purified from gel. The results were calculated in MS Excel 2019 using the exponential function equation or the trend function.

DNA electrophoresis of 3C libraries

After restriction digestion with DpnII, chromatin digested efficiently appears as a smear that starts from an about 6-kb marker and has a density center around 1-1.5 kb in agarose gel electrophoresis pictures. 3C libraries obtained after DNA ligation form smears that migrate a little below or above a

10-kb marker. A more concentrated smear at the top indicates more efficient ligation. The longer the DNA smear, the poorer is the ligation efficiency.

DNA electrophoresis of 3C libraries was done in freshly prepared 1X TAE (of the following 50X composition: Tris 24.2g, EDTA 1.46g, ~5.7 ml glacial acetic acid (titrate until pH drops to 8.6)) and 1.25% agarose gels (TopVision Agarose, Thermo #R0492) prestained with ethidium bromide in the 1X TAE buffer in Helikon SE-2 electrophoresis camera at 100V (3.7V/cm) for 45-50 min. The width of a comb, number of teeth in the comb, and the length of each tooth were 1.0 mm, 18, and 0.4 mm, respectively. DNA resolution was monitored using a MassRuller High range DNA Ladder (Thermo #SM0393, 1500-10 000 bp) mixed 1:1 with a MassRuler Low range DNA Ladder (Thermo #SM0383, 80-1031 bp) or using a GeneRuler DNA Ladder Mix (Thermo #SM0331, 100-10 000 bp, bright bands are 500, 1000, 3000 bp). For electrophoresis, 1 µl of a 3C library was typically mixed with 15 µl of 10 mM Tris-HCl (pH 8.0) and no more than 0.4-0.5 µl of Purple Gel loading dye 6X (NEB #B7024S) or TriTrack DNA loading dye (Thermo #R1161). The same manipulation was done for 0.3-0.4 µl of GeneRuler DNA Ladder Mix DNA marker.

Plasmid DNA preparation

Plasmid pUC19 (Addgene #50005) and pU6-BbsI-chiRNA (Addgene #45946) DNAs were isolated from *E. coli* DH5α strain using a GeneJET Plasmid Miniprep Kit (Thermo #K0503) according to the manufacturer's instructions.

Table S8. List of primers and TaqMan probes

Abbreviation	Destination	Sequence
Enh Dad13	Anchor primer at *Dad* Enhancer 13	5'- TTCCCCCTTACCAATCGTTCC
Enh Dad13_test	Tester primer next to *Dad* Enhancer 13	5'- CCCGCCCTCTTTCACCAA
PlacZ	Anchor primer at $P_{lexAop\text{-}hsp70}$-lacZ promoter	5'- CAAAGTGAACACGTCGCTAAGC
PlacZ_test	Tester primer next to $P_{lexAop\text{-}hsp70}$-*lacZ* promoter	5'- CAGACCAATGCCTCCCAGAC
Enh Dad13 probe	probe at *Dad* Enhancer 13	5'- (FAM)CCCACCATCG(T-BHQ1)CCGTCTCTTTCTCGCTG-P
PlacZ probe	probe at $P_{lexAop\text{-}hsp70}$ promoter	5'- (FAM)TAACCAGCAACCAAG(T-BHQ1)AAATCAACTGCAACT-P

RpII_1_Forward	DpnII site regeneration, normalization in *RpII*	5'- GGGGCGACCAGAAGAAGGC
RpII_2_Reverse#1	DpnII site regeneration, normalization in *RpII*	5'- CCGCAAATGGGAAAGAGTAGAGG
RpII_3_Reverse#2	DpnII site regeneration, normalization in *RpII*	5'- GCGATGGCAGAAGGAGCAAT
RpII_0 probe	DpnII site regeneration, normalization in *RpII*	5'- (FAM)TCAAGCGAT(T-BHQ1)CAACACCTGGGAGACACCG-P
RpII_22_Reverse	Circularization measurement	5'- GCCTTCTTCTGGTCGCCCC
RpII_11_Forward	Circularization measurement	5'- CCTCTACTCTTTCCCATTTGCGG
RpII_00 probe	Circularization measurement	5'- (FAM)TCAGGAGTT(T-BHQ1)CAGCGGGACACAACATACC-P

4 References

Bajorath, J., Hinrichs, W., and Saenger, W. (1988). The enzymatic activity of proteinase K is controlled by calcium. *European Journal of Biochemistry* 176, 441–447. doi:https://doi.org/10.1111/j.1432-1033.1988.tb14301.x.

Belaghzal, H., Dekker, J., and Gibcus, J. H. (2017). Hi-C 2.0: An optimized Hi-C procedure for high-resolution genome-wide mapping of chromosome conformation. *Methods* 123, 56–65. doi:10.1016/j.ymeth.2017.04.004.

Belton, J.-M., McCord, R. P., Gibcus, J., Naumova, N., Zhan, Y., and Dekker, J. (2012). Hi-C: A comprehensive technique to capture the conformation of genomes. *Methods* 58. doi:10.1016/j.ymeth.2012.05.001.

Campos, P. F., and Gilbert, T. M. P. (2012). DNA extraction from formalin-fixed material. *Methods Mol Biol* 840, 81–85. doi:10.1007/978-1-61779-516-9_11.

Canela, A., Maman, Y., Jung, S., Wong, N., Callen, E., Day, A., et al. (2017). Genome Organization Drives Chromosome Fragility. *Cell* 170, 507-521.e18. doi:10.1016/j.cell.2017.06.034.

Comet, I., Schuettengruber, B., Sexton, T., and Cavalli, G. (2011). A chromatin insulator driving three-dimensional Polycomb response element (PRE) contacts and Polycomb association with the chromatin fiber. *PNAS* 108, 2294–2299. doi:10.1073/pnas.1002059108.

Dekker, J., Rippe, K., Dekker, M., and Kleckner, N. (2002). Capturing chromosome conformation. *Science* 295, 1306–1311. doi:10.1126/science.1067799.

Ebeling, W., Hennrich, N., Klockow, M., Metz, H., Orth, H. D., and Lang, H. (1974). Proteinase K from Tritirachium album Limber. *European Journal of Biochemistry* 47, 91–97. doi:https://doi.org/10.1111/j.1432-1033.1974.tb03671.x.

Fattorini, P., Marrubini, G., Bonin, S., Bertoglio, B., Grignani, P., Recchia, E., et al. (2018). Prolonged DNA hydrolysis in water: A study on DNA stability. *Data in Brief* 20, 1237–1243. doi:10.1016/j.dib.2018.08.120.

Flyamer, I. M., Gassler, J., Imakaev, M., Brandão, H. B., Ulianov, S. V., Abdennur, N., et al. (2017). Single-nucleus Hi-C reveals unique chromatin reorganization at oocyte-to-zygote transition. *Nature* 544, 110–114. doi:10.1038/nature21711.

Fujiwara, Y., Wada, K., and Kabuta, T. (2017). Lysosomal degradation of intracellular nucleic acids—multiple autophagic pathways. *The Journal of Biochemistry* 161, 145–154. doi:10.1093/jb/mvw085.

Gavrilov, A. A. (2016). Doctor of Sc. Thesis. Spatial organization of the eukaryote genome in the context of transcription regulation. Available at: http://www.genebiology.ru/dissovet/o-dissertatcionnom-sovete/.

Golov, A. K., Gavrilov, A. A., and Razin, S. V. (2015). The Role of Crowding Forces in Juxtaposing β-Globin Gene Domain Remote Regulatory Elements in Mouse Erythroid Cells. *PLOS ONE* 10, e0139855. doi:10.1371/journal.pone.0139855.

Golov, A. K., Ulianov, S. V., Luzhin, A. V., Kalabusheva, E. P., Kantidze, O. L., Flyamer, I. M., et al. (2020). C-TALE, a new cost-effective method for targeted enrichment of Hi-C/3C-seq libraries. *Methods* 170, 48–60. doi:10.1016/j.ymeth.2019.06.022.

Hilz, H., Wiegers, U., and Adamietz, P. (1975). Stimulation of Proteinase K Action by Denaturing Agents: Application to the Isolation of Nucleic Acids and the Degradation of 'Masked' Proteins. *European Journal of Biochemistry* 56, 103–108. doi:https://doi.org/10.1111/j.1432-1033.1975.tb02211.x.

Hoffman, E. A., Frey, B. L., Smith, L. M., and Auble, D. T. (2015). Formaldehyde crosslinking: a tool for the study of chromatin complexes. *J. Biol. Chem.* 290, 26404–26411. doi:10.1074/jbc.R115.651679.

Kalhor, R., Tjong, H., Jayathilaka, N., Alber, F., and Chen, L. (2012). Genome architectures revealed by tethered chromosome conformation capture and population-based modeling. *Nature Biotechnology* 30, 90–98. doi:10.1038/nbt.2057.

Kawane, K., Motani, K., and Nagata, S. (2014). DNA Degradation and Its Defects. *Cold Spring Harb Perspect Biol* 6. doi:10.1101/cshperspect.a016394.

Kennedy-Darling, J., and Smith, L. M. (2014). Measuring the Formaldehyde Protein–DNA Cross-Link Reversal Rate. *Anal. Chem.* 86, 5678–5681. doi:10.1021/ac501354y.

Lechardeur, D., Sohn, K.-J., Haardt, M., Joshi, P. B., Monck, M., Graham, R. W., et al. (1999). Metabolic instability of plasmid DNA in the cytosol: a potential barrier to gene transfer. *Gene Ther* 6, 482–497. doi:10.1038/sj.gt.3300867.

Lieberman-Aiden, E., van Berkum, N. L., Williams, L., Imakaev, M., Ragoczy, T., Telling, A., et al. (2009). Comprehensive mapping of long-range interactions reveals folding principles of the human genome. *Science* 326, 289–293. doi:10.1126/science.1181369.

Louwers, M., Splinter, E., van Driel, R., de Laat, W., and Stam, M. (2009). Studying physical chromatin interactions in plants using Chromosome Conformation Capture (3C). *Nat Protoc* 4, 1216–1229. doi:10.1038/nprot.2009.113.

Marko, A. M., and Butler, G. C. (1951). The Isolation of Sodium Desoxyribonucleate with Sodium Dodecyl Sulfate. *J. Biol. Chem.* 190, 165–176.

Méndez, J., and Stillman, B. (2000). Chromatin Association of Human Origin Recognition Complex, Cdc6, and Minichromosome Maintenance Proteins during the Cell Cycle: Assembly of Prereplication Complexes in Late Mitosis. *Molecular and Cellular Biology* 20, 8602–8612. doi:10.1128/MCB.20.22.8602-8612.2000.

Miele, A., Gheldof, N., Tabuchi, T. M., Dostie, J., and Dekker, J. (2006a). Mapping Chromatin Interactions by Chromosome Conformation Capture. *Current Protocols in Molecular Biology* 74, 21.11.1-21.11.20. doi:10.1002/0471142727.mb2111s74.

Miele, A., Gheldof, N., Tabuchi, T. M., Dostie, J., and Dekker, J. (2006b). Mapping chromatin interactions by chromosome conformation capture. *Curr Protoc Mol Biol* Chapter 21, Unit 21.11. doi:10.1002/0471142727.mb2111s74.

Nagano, T., Lubling, Y., Stevens, T. J., Schoenfelder, S., Yaffe, E., Dean, W., et al. (2013). Single-cell Hi-C reveals cell-to-cell variability in chromosome structure. *Nature* 502, 59–64. doi:10.1038/nature12593.

Nagano, T., Lubling, Y., Várnai, C., Dudley, C., Leung, W., Baran, Y., et al. (2017). Cell-cycle dynamics of chromosomal organization at single-cell resolution. *Nature* 547, 61–67. doi:10.1038/nature23001.

Nagano, T., Lubling, Y., Yaffe, E., Wingett, S. W., Dean, W., Tanay, A., et al. (2015a). Single-cell Hi-C for genome-wide detection of chromatin interactions that occur simultaneously in a single cell. *Nature Protocols* 10, 1986–2003. doi:10.1038/nprot.2015.127.

Nagano, T., Várnai, C., Schoenfelder, S., Javierre, B.-M., Wingett, S. W., and Fraser, P. (2015b). Comparison of Hi-C results using in-solution versus in-nucleus ligation. *Genome Biology* 16, 175. doi:10.1186/s13059-015-0753-7.

Naumova, N., Smith, E. M., Zhan, Y., and Dekker, J. (2012). Analysis of long-range chromatin interactions using Chromosome Conformation Capture. *Methods* 58, 192–203. doi:10.1016/j.ymeth.2012.07.022.

O'Lenick, A. (2018). Understanding Salt Curves. *Science for formulators*. doi:10.1111/j.0142-.

Qamar, W., Khan, M. R., and Arafah, A. (2017). Optimization of conditions to extract high quality DNA for PCR analysis from whole blood using SDS-proteinase K method. *Saudi J Biol Sci* 24, 1465–1469. doi:10.1016/j.sjbs.2016.09.016.

Rao, S. S. P., Huntley, M. H., Durand, N. C., Stamenova, E. K., Bochkov, I. D., Robinson, J. T., et al. (2014). A three-dimensional map of the human genome at kilobase resolution reveals principles of chromatin looping. *Cell* 159, 1665–1680. doi:10.1016/j.cell.2014.11.021.

Ruiz-Villalba, A., van Pelt-Verkuil, E., Gunst, Q. D., Ruijter, J. M., and van den Hoff, M. J. (2017). Amplification of nonspecific products in quantitative polymerase chain reactions (qPCR). *Biomol Detect Quantif* 14, 7–18. doi:10.1016/j.bdq.2017.10.001.

Samal, B. B., Karan, B., Parker, C., and Stabinsky, Y. (1991). Isolation and thermal stability studies of two novel serine proteinases from the fungus Tritirachium album Limber. *Enzyme Microb Technol* 13, 66–70. doi:10.1016/0141-0229(91)90190-l.

Shechter, D., Dormann, H., Allis, C., and Hake, S. (2007). Extraction, purification and analysis of histones. *Nature Protocols* 2, 1445–1457. doi:10.1038/nprot.2007.202.

Shidlovskii, Y. V., Bylino, O. V., Shaposhnikov, A. V., Kachaev, Z. M., Lebedeva, L. A., Kolesnik, V. V., et al. (2021). Subunits of the PBAP Chromatin Remodeler Are Capable of Mediating Enhancer-Driven Transcription in Drosophila. *Int J Mol Sci* 22. doi:10.3390/ijms22062856.

Sima, J., Chakraborty, A., Dileep, V., Michalski, M., Klein, K. N., Holcomb, N. P., et al. (2019). Identifying cis Elements for Spatiotemporal Control of Mammalian DNA Replication. *Cell* 176, 816-830.e18. doi:10.1016/j.cell.2018.11.036.

Splinter, E., de Wit, E., van de Werken, H. J. G., Klous, P., and de Laat, W. (2012). Determining long-range chromatin interactions for selected genomic sites using 4C-seq technology: from fixation to computation. *Methods* 58, 221–230. doi:10.1016/j.ymeth.2012.04.009.

Splinter, E., Grosveld, F., and de Laat, W. (2004). 3C technology: analyzing the spatial organization of genomic loci in vivo. *Methods in Enzymology* 375, 493–507. doi:10.1016/s0076-6879(03)75030-7.

Splinter, E., Heath, H., Kooren, J., Palstra, R.-J., Klous, P., Grosveld, F., et al. (2006). CTCF mediates long-range chromatin looping and local histone modification in the beta-globin locus. *Genes Dev* 20, 2349–2354. doi:10.1101/gad.399506.

Stadhouders, R., Kolovos, P., Brouwer, R., Zuin, J., van den Heuvel, A., Kockx, C., et al. (2013a). Multiplexed chromosome conformation capture sequencing for rapid genome-scale high-resolution detection of long-range chromatin interactions. *Nat Protoc* 8, 509–524. doi:10.1038/nprot.2013.018.

Stadhouders, R., Kolovos, P., Brouwer, R., Zuin, J., van den Heuvel, A., Kockx, C., et al. (2013b). Multiplexed chromosome conformation capture sequencing for rapid genome-scale high-resolution detection of long-range chromatin interactions. *Nature Protocols* 8, 509–524. doi:10.1038/nprot.2013.018.

Tolhuis, B., Palstra, R.-J., Splinter, E., Grosveld, F., and de Laat, W. (2002). Looping and Interaction between Hypersensitive Sites in the Active β-globin Locus. *Molecular Cell* 10, 1453–1465. doi:10.1016/S1097-2765(02)00781-5.

Ulianov, S. V., Khrameeva, E. E., Gavrilov, A. A., Flyamer, I. M., Kos, P., Mikhaleva, E. A., et al. (2016). Active chromatin and transcription play a key role in chromosome partitioning into topologically associating domains. *Genome Res.* 26, 70–84. doi:10.1101/gr.196006.115.

Ulianov, S. V., Zakharova, V. V., Galitsyna, A. A., Kos, P. I., Polovnikov, K. E., Flyamer, I. M., et al. (2021). Order and stochasticity in the folding of individual Drosophila genomes. *Nature Communications* 12, 41. doi:10.1038/s41467-020-20292-z.

van Berkum, N. L., and Dekker, J. (2009). “Determining Spatial Chromatin Organization of Large Genomic Regions Using 5C Technology,” in *Chromatin Immunoprecipitation Assays: Methods and Protocols* Methods in Molecular Biology., ed. P. Collas (Totowa, NJ: Humana Press), 189–213. doi:10.1007/978-1-60327-414-2_13.

van de Werken, H. J. G., de Vree, P. J. P., Splinter, E., Holwerda, S. J. B., Klous, P., de Wit, E., et al. (2012). “4C Technology: Protocols and Data Analysis,” in *Methods in Enzymology* (Elsevier), 89–112. doi:10.1016/B978-0-12-391938-0.00004-5.

Vermeulen, C., Allahyar, A., Bouwman, B. A. M., Krijger, P. H. L., Verstegen, M. J. A. M., Geeven, G., et al. (2020). Multi-contact 4C: long-molecule sequencing of complex proximity ligation products to uncover local cooperative and competitive chromatin topologies. *Nature Protocols* 15, 364–397. doi:10.1038/s41596-019-0242-7.

von Holt, C., Brandt, W. F., Greyling, H. J., Lindsey, G. G., Retief, J. D., de A. Rodrigues, J., et al. (1989). “[23] Isolation and characterization of histones,” in *Methods in Enzymology* (Elsevier), 431–523. doi:10.1016/0076-6879(89)70061-6.

Yang, W. (2011). Nucleases: diversity of structure, function and mechanism. *Q Rev Biophys* 44, 1–93. doi:10.1017/S0033583510000181.

Yuen, P. S. T., Brooks, K. M., and Li, Y. (2001). RNA: a method to specifically inhibit PCR amplification of known members of a multigene family by degenerate primers. *Nucleic Acids Res* 29, e31.
